# The genome sequence of the grape phylloxera provides insights into the evolution, adaptation, and invasion routes of an iconic pest

**DOI:** 10.1186/s12915-020-00820-5

**Published:** 2020-07-23

**Authors:** Claude Rispe, Fabrice Legeai, Paul D. Nabity, Rosa Fernández, Arinder K. Arora, Patrice Baa-Puyoulet, Celeste R. Banfill, Leticia Bao, Miquel Barberà, Maryem Bouallègue, Anthony Bretaudeau, Jennifer A. Brisson, Federica Calevro, Pierre Capy, Olivier Catrice, Thomas Chertemps, Carole Couture, Laurent Delière, Angela E. Douglas, Keith Dufault-Thompson, Paula Escuer, Honglin Feng, Astrid Forneck, Toni Gabaldón, Roderic Guigó, Frédérique Hilliou, Silvia Hinojosa-Alvarez, Yi-min Hsiao, Sylvie Hudaverdian, Emmanuelle Jacquin-Joly, Edward B. James, Spencer Johnston, Benjamin Joubard, Gaëlle Le Goff, Gaël Le Trionnaire, Pablo Librado, Shanlin Liu, Eric Lombaert, Hsiao-ling Lu, Martine Maïbèche, Mohamed Makni, Marina Marcet-Houben, David Martínez-Torres, Camille Meslin, Nicolas Montagné, Nancy A. Moran, Daciana Papura, Nicolas Parisot, Yvan Rahbé, Mélanie Ribeiro Lopes, Aida Ripoll-Cladellas, Stéphanie Robin, Céline Roques, Pascale Roux, Julio Rozas, Alejandro Sánchez-Gracia, Jose F. Sánchez-Herrero, Didac Santesmasses, Iris Scatoni, Rémy-Félix Serre, Ming Tang, Wenhua Tian, Paul A. Umina, Manuella van Munster, Carole Vincent-Monégat, Joshua Wemmer, Alex C. C. Wilson, Ying Zhang, Chaoyang Zhao, Jing Zhao, Serena Zhao, Xin Zhou, François Delmotte, Denis Tagu

**Affiliations:** 1 BIOEPAR, INRAE, Oniris, Nantes, France; 2 BIPAA, IGEPP, Agrocampus Ouest, INRAE, Université de Rennes 1, 35650 Le Rheu, France; 3 Department of Botany and Plant Sciences, University of California, Riverside, USA; 4 Bioinformatics and Genomics Unit, Centre for Genomic Regulation (CRG), Barcelona Institute of Science and Technology, Dr. Aiguader, 88, 08003 Barcelona, Spain; 5 Present address: Institute of Evolutionary Biology (CSIC-UPF), Passeig marítim de la Barceloneta 37-49, 08003 Barcelona, Spain; 6 Department of Entomology, Cornell University, Ithaca, NY 14853 USA; 7 Univ Lyon, INSA-Lyon, INRAE, BF2I, UMR0203, F-69621, Villeurbanne, France; 8 Department of Biology, University of Miami, Coral Gables, FL 33146 USA; 9 Facultad de Agronomía, Montevideo, Uruguay; 10 Institut de Biologia Integrativa de Sistemes, Parc Cientific Universitat de Valencia, C/ Catedrático José Beltrán n° 2, 46980 Paterna, València Spain; 11 Université de Tunis El Manar, Faculté des Sciences de Tunis, LR01ES05 Biochimie et Biotechnologie, 2092 Tunis, Tunisia; 12 Department Biol, Univ Rochester, Rochester, NY 14627 USA; 13 Laboratoire Evolution, Génomes, Comportement, Ecologie CNRS, Univ. Paris-Sud, IRD, Université Paris-Saclay, Gif-sur-Yvette, France; 14 LIPM, Université de Toulouse, INRAE, CNRS, Castanet-Tolosan, France; 15 Sorbonne Université, UPEC, Université Paris 7, INRAE, CNRS, IRD, Institute of Ecology and Environmental Sciences, Paris, France; 16 SAVE, INRAE, Bordeaux Sciences Agro, Villenave d’Ornon, France; 17 Department of Molecular Biology and Genetics, Cornell University, Ithaca, NY 14853 USA; 18 Department of Cell and Molecular Biology, College of the Environment and Life Sciences, University of Rhode Island, Kingston, RI USA; 19 Departament de Genètica, Microbiologia i Estadística and Institut de Recerca de la Biodiversitat (IRBio), Universitat de Barcelona, 08028 Barcelona, Spain; 20 Department of Biology, University of Miami, Coral Gables, USA; 21 Current affiliation: Boyce Thompson Institute for Plant Research, Cornell University, Ithaca, USA; 22 Universität für Bodenkultur (BOKU), Vienna, Austria; 23 Universitat Pompeu Fabra, 08003 Barcelona, Spain; 24 Institució Catalana de Recerca i Estudis Avançats (ICREA), Pg. Lluís Companys 23, 08010 Barcelona, Spain; 25 Centre for Genomic Regulation (CRG), The Barcelona Institute of Science and Technology, Barcelona, Spain; 26 Universitat Pompeu Fabra (UPF), Barcelona, Spain; 27 Université Côte d’Azur, INRAE, CNRS, Institut Sophia Agrobiotech, Sophia-Antipolis, France; 28 Institute of Biotechnology and Department of Entomology, College of Bioresources and Agriculture, National Taiwan University, Taipei, Taiwan; 29 Present affiliation: Bone and Joint Research Center, Chang Gung Memorial Hospital, Taoyuan, Taiwan; 30 IGEPP, Agrocampus Ouest, INRAE, Université de Rennes 1, 35650 Le Rheu, France; 31 INRAE, Institute of Ecology and Environmental Sciences, Versailles, France; 32 Department of Entomology, Texas A&M University, College Station, TX 77843 USA; 33 Université Côte d’Azur, INRAE, CNRS, Institut Sophia Agrobiotech, Sophia-Antipolis, France; 34 Laboratoire d’Anthropobiologie Moléculaire et d’Imagerie de Synthèse, CNRS UMR 5288, Université de Toulouse, Université Paul Sabatier, Toulouse, France; 35 China National GeneBank-Shenzhen, BGI-Shenzhen, Shenzhen, 518083 Guangdong Province People’s Republic of China; 36 BGI-Shenzhen, Shenzhen, 518083 Guangdong Province People’s Republic of China; 37 Department of Entomology, College of Plant Protection, China Agricultural University, Beijing, 100193 People’s Republic of China; 38 Université Côte d’Azur, INRAE, CNRS, ISA, Sophia Antipolis, France; 39 Department of Post-Modern Agriculture, MingDao University, Changhua, Taiwan; 40 Institut de Biologia Integrativa de Sistemes, Parc Cientific Universitat de Valencia, C/ Catedrático José Beltrán n° 2, 46980 Paterna, València Spain; 41 Sorbonne Université, Institute of Ecology and Environmental Sciences, Paris, France; 42 Department of Integrative Biology, University of Texas at Austin, Austin, USA; 43 Univ Lyon, INRAE, INSA-Lyon, CNRS, UCBL, UMR5240 MAP, F-69622 Villeurbanne, France; 44 BIPAA IGEPP, Agrocampus Ouest, INRAE, Université de Rennes 1, 35650 Le Rheu, France; 45 Plateforme Génomique GeT-PlaGe, Centre INRAE de Toulouse Midi-Pyrénées, 24 Chemin de Borde Rouge, Auzeville, CS 52627, 31326 Castanet-Tolosan Cedex, France; 46 Division of Genetics, Department of Medicine, Brigham and Women’s Hospital, Harvard Medical School, Boston, MA 02115 USA; 47 Facultad de Agronoía, Montevideo, Uruguay; 48 School of BioSciences, The University of Melbourne, Parkville, VIC Australia; 49 BGPI, Université Montpellier, CIRAD, INRAE, Montpellier SupAgro, Montpellier, France

**Keywords:** Arthropod genomes, *Daktulosphaira vitifoliae*, Gene duplications, Host plant interactions, Effectors, Biological invasions

## Abstract

**Background:**

Although native to North America, the invasion of the aphid-like grape phylloxera *Daktulosphaira vitifoliae* across the globe altered the course of grape cultivation. For the past 150 years, viticulture relied on grafting-resistant North American *Vitis* species as rootstocks, thereby limiting genetic stocks tolerant to other stressors such as pathogens and climate change. Limited understanding of the insect genetics resulted in successive outbreaks across the globe when rootstocks failed. Here we report the 294-Mb genome of *D. vitifoliae* as a basic tool to understand host plant manipulation, nutritional endosymbiosis, and enhance global viticulture.

**Results:**

Using a combination of genome, RNA, and population resequencing, we found grape phylloxera showed high duplication rates since its common ancestor with aphids, but similarity in most metabolic genes, despite lacking obligate nutritional symbioses and feeding from parenchyma. Similarly, no enrichment occurred in development genes in relation to viviparity. However, phylloxera evolved > 2700 unique genes that resemble putative effectors and are active during feeding. Population sequencing revealed the global invasion began from the upper Mississippi River in North America, spread to Europe and from there to the rest of the world.

**Conclusions:**

The grape phylloxera genome reveals genetic architecture relative to the evolution of nutritional endosymbiosis, viviparity, and herbivory. The extraordinary expansion in effector genes also suggests novel adaptations to plant feeding and how insects induce complex plant phenotypes, for instance galls. Finally, our understanding of the origin of this invasive species and its genome provide genetics resources to alleviate rootstock bottlenecks restricting the advancement of viticulture.

## Introduction

Biological invasions can affect ecosystems and severely impact human societies and economies by threatening global food production when the invader is a pest or pathogen [1]. How invading species become so successful in their new environments remains enigmatic, and although numerous hypotheses are supported by various organisms [2], deciphering the genetics underlying invaders provides deep insight into population or genotype-specific success [3]. Few biological invasions have wreaked as much havoc on a cultivated plant species as the grape phylloxera, *Daktulosphaira vitifoliae* (Fitch), did on the European grape, *Vitis vinifera* [4, 5]. The accidental introduction of *D. vitifoliae* in the 1860s from its native range in North America to France precipitated the start of a “phylloxeric plague” that rapidly spreads across Europe and later to other grape-growing regions of the world [6, 7], wiping out many vineyards. But it took several years to identify *D. vitifoliae* as the causative agent, largely through a fruitful collaboration between C. V. Riley (USA) and J.-E. Planchon (France) [8]. Yet, in the 150 years since the invasion began, little is known about how *D. vitifoliae* spread or what enables its success across *Vitis* species.

**Fig. 1:**
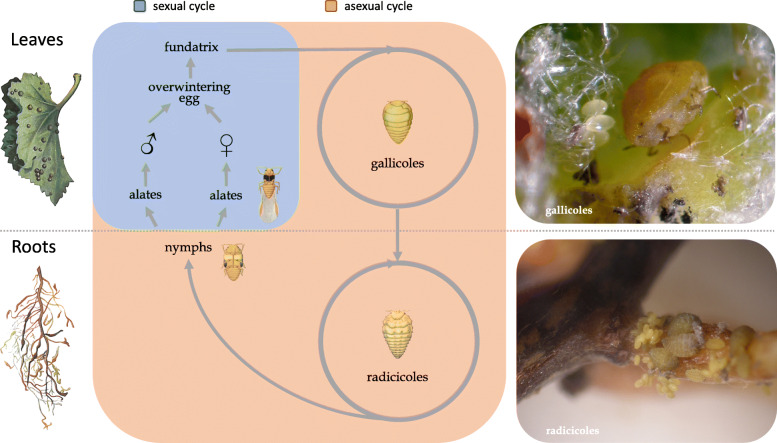
The life cycle of grape phylloxera, alternating between a sexual phase and an asexual phase. Asexual females can feed either on leaves where they form galls (gallicoles) or on roots (radicicoles) of *Vitis* species. Eggs resulting from sexual reproduction hatch in the spring to produce a mobile stage (fundatrix) that initiates a colony on leaves. Gallicoles and radicicoles then undergo several asexual generations during a season. First instars of gallicoles and radicicoles are also mobile forms (crawlers) that allow the establishment of new feeding sites on active growing shoot tips or roots. Gallicoles first instars can migrate to roots, giving rise to radicicoles. Under inducing conditions, radicicoles can give rise to nymphal instars which emerge from the ground and molt to the alate adult stage achieving long-range dispersal. Alates, although morphologically identical, either engender exclusively sexual females or males. After mating, each sexual female lays a unique overwintering egg. Radicicoles can alternatively overwinter as first instar hibernants, implying a possible permanent looping of asexual cycles on roots. Gallicoles are the typical form found on native *Vitis* sp. in North America while radicicoles are most common on the cultivated varieties of *Vitis vinifera* throughout the world. Drawings included in the figure were taken from [9]

*D. vitifoliae* is a minuscule cyclically parthenogenetic insect, alternating sexual and asexual reproduction, like aphids, a related group (Fig. 1). But unlike aphids, which are viviparous in asexual stages, feed on phloem sap, and are associated with the endosymbiont *Buchnera* [10], phylloxera is oviparous at all stages, feeds on parenchymatous cells, and does not have a known obligatory bacterial endosymbiont. A further peculiarity of grape phylloxera compared to other species of its group, Phylloxeroidea, is that this insect feeds either underground on roots or on leaves (Fig. 1). Leaf-galling forms are predominant on native American *Vitis* species whereas root galling is the predominant feeding mode in cultivated varieties of *V. vinifera* worldwide. Indeed, symptoms on leaves of cultivated vines are barely observed, suggesting rarity of sexuality [11]. Root feeding is lethal on cultivated grapevine as it creates wounds that are vulnerable to entry of soil-borne fungal and bacterial pathogens [12].

Viticulture in Europe was rescued by the discovery that many *Vitis* species of American origin exhibit tolerance or resistance to *D. vitifoliae* and could be used as rootstock for grafting *V. vinifera* cultivars, thereby retaining desirable characteristics of the fruit [8]. This grafting solution exploits the coevolutionary relationship between parasite and host in native populations that resulted in the coexistence of these species. This approach has proven a successful management strategy worldwide. However, past rootstock failures [12] and the use of non-grafted vines in some regions of the world (Australia, Chile, China, and occasionally in the USA) demand constant surveillance for phylloxera infestation to prevent invasions. Ultimately, the overall success of grafting as a control strategy precipitated decreased research on phylloxera biology. Thus, many aspects of *D. vitifoliae* ecology, evolution, and population genetics, including knowledge of how its genetic architecture enables or is constrained by interactions with its host plants, remained unknown.

Genome sequencing of the grape phylloxera—with annotation performed with the help of the International Aphid Genomics Consortium—has allowed us to address evolutionary processes shaping the biology of this organism at different time-scales. First, our comparative analyses allowed us to evaluate ancient evolutionary events dating back to the common ancestor between phylloxera and aphids or earlier. Grape phylloxera is related to aphids, a group with which it shares important evolutionary innovations (such as cyclical parthenogenesis, the alternation of sexual and asexual reproduction) but differs in other traits (strict oviparity, lack of obligate endosymbionts). We expected the genome sequence to exhibit evidence of these differences, in terms of gene repertoires and metabolic pathways. Also, given that aphids retain an exceptional level of gene duplication [13, 14], we examined if this observation extended to phylloxera, or even to a common ancestor of the Sternorrhyncha, the group of plant-feeding insects that includes phylloxera and aphid. We then analyzed patterns of gene expansion along the phylogenetic tree to better understand how plant feeding alters the evolution of herbivore genomes. Second, we addressed more recent evolutionary processes that influenced the genomes of each lineage (e.g., aphids versus phylloxera). Nutritional constraints, resulting from a strict diet of plant sap, are expected to have affected the genomes of both aphids and phylloxera, with expected common points and differences linked to the shared or unique traits between these groups. To address this question, we compared genome repertoires, which pointed to rapid changes possibly shaped by intense evolutionary pressure in the context of host plant specialization and manipulation. Third, our work addresses a very recent biological event, the invasion of Europe, and other grape-producing regions by phylloxera. With the intention to trace back the geographical routes of this invasion, we performed genome-wide sequencing of phylloxera samples from both the native (North America) and introduced populations (Europe and New World vineyards).

Our study, supported by a highly complete genome and an uncommon community effort on curated annotation, revealed that:

- Phylloxera (like aphids) has a high number of coding genes compared to other arthropods, with both an increased level of gene duplication mapped to the common ancestor of aphids and phylloxera and high rates of recent duplications
- An extraordinarily large expansion of a novel gene family is comprised of putative effectors; we expect that they represent a key component of the interactions and adaptation between this insect species and its host plants
- Phylloxera populations of the upper Mississippi River basin, feeding on the wild species *Vitis riparia*, are likely to be the principal source of the invasion to Europe. Subsequent invasions of South America and western Australia were the result of secondary introductions, from European sources

## Results and discussion

### Genome features

The haploid genome size of the *D. vitifoliae* Pcf7 strain was estimated by flow cytometry at 294 Mb by two independent measures (± SD = 1 Mb with *Drosophila melanogaster* and ± SD = 5 Mb with alfalfa as references, respectively). The final assembly (v3.1) summed to 282.7 Mb, a total close to that estimated by flow cytometry. The genome assembly comprised 10,492 scaffolds with a median size of 1077 bp and an N50 of 342 kb. A BUSCO analysis based on insect conserved genes indicated the presence of 94.2% of these as complete genes (Table 1). A total of 24,581 genes (OGS 3.0) were automatically predicted. Extensive manual annotation (see below) led to gene corrections of more than 15% of the inspected genes as well as new gene detection (see the “Effectors” section), such that the final gene catalog contained 25,814 predicted genes and 25,825 transcripts (OGS 3.2). The genomic GC content was low for an arthropod (27.2%) but comparable to that of other aphid genomes (e.g., 27.8% for *A. pisum*, 30.1% for *M. persicae* [13, 14]). The recovered mitochondrial genome had gene content and order typical of insect and aphid mitochondrial genomes with 13 protein-coding genes, 22 tRNA genes, and 2 rRNA genes (Additional File 1: Fig.S1): the *D. vitifoliae* mitochondrial scaffold was smaller (15,568 bp) than the mitochondrial genomes from the pea aphid, *Acyrthosiphon pisum* (16,971 bp), and the fruit fly, *Drosophila melanogaster* (19,517 bp), and had similar GC content to both other species (15.5% vs 15.2% and 17.8%, respectively) [13].

**Table 1:** Assembly parameters and genome features of the grape phylloxera genome, version V3.1

Parameters	Numbers
**Assembly**	Version 3.1
**Contigs**	
Total assembly size	282,671,353
Number of contigs	17,162
Contig N50 length (bp)	74,750
Longest contig (bp)	718,286
Shortest contig (bp)	83
Number of contigs > 10 kb	4914
Mean (median) contig size, in bp	16,107 (1635)
**Scaffolds**	
Number of scaffolds	10,492
Longest scaffold (bp)	2,080,308
Shortest scaffold (bp)	141
Number of scaffolds > 1 Mb	19
Mean (median) scaffold size, in bp	26,942 (1077)
N50 scaffold length (bb)	341,590
**Genomic features (OGS 3.2)**	
Mean transcripts length (bp)	4653
Mean CDS length (bp)	1053
Mean exon length (bp)	244
Mean exon number per gene	5.4
Gene count	25,825
**BUSCO analysis (genome v3.1)**	
Complete BUSCO	1563/1658 (94.2%)
Complete and single-copy BUSCOs	1531/1658 (92.3%)
Complete and duplicated BUSCOs	32/1658 (1.9%)
Fragmented BUSCOs	26/1658 (1.6%)
Missing BUSCOs	69/1658 (4.2%)

#### Horizontal gene transfer from bacteria and fungi into the phylloxera genome

Genomes of Aphididae and Adelgidae species were previously shown to contain genes underlying carotenoid biosynthesis as the result of a horizontal transfer event from a fungus [15]. Homologs of these genes were recently found to be present in nine Phylloxeridae species [16], including the grape phylloxera. Confirming these results, our searches of the phylloxera genome revealed that the carotenoid biosynthetic gene cluster is present, as a single copy, and containing the fused phytoene synthase/lycopene cyclase that is characteristic of aphids and of some fungi (Additional File 1: Table S1) [17, 18]. Based on BLASTp searches (*e* value cutoff = 0.01) of published genomes using query sequences from *A. pisum*, homologs of these genes appear to be absent from sequenced genomes of the Psyllidae and Aleyrodidae. Phytoene desaturase is present in adelgids based on PCR amplification and Sanger sequencing [15], but genome sequences of adelgids are not available for further screening. Based on this distribution, it is likely that these fungal genes were transferred to an ancestor of all Aphidomorpha (Aphididae, Adelgidae, Phylloxeridae) in one event and underwent subsequent duplications in lineages of Aphididae.

The *A. pisum* genome also contains genes of bacterial origin (*ldcA*, *rlpA*, and *amiD*) that are highly expressed in the bacteriocytes housing the obligate bacterial endosymbiont *Buchnera aphidicola*, but that were acquired from bacterial sources other than the symbionts [17, 18]. None of these genes could be found in the phylloxera genome. Because Phylloxeridae lack obligate bacterial symbionts, the absence of these genes is consistent with the hypothesis that they were acquired by ancestral aphids in the context of adaptation for the obligate symbiosis. The absence of these genes could reflect loss in Phylloxeridae or acquisition in Aphididae after divergence from Phylloxeridae (Additional File 1: Table S1).

#### Repetitive DNA

In addition, 317,612 TE copies were identified; these constitute 119 Mb, or 42.2% of the draft sequence (Additional File 1: Fig.S2) [19], slightly above the 38% found in *A. pisum* [13] and the maximum for known hemipteran genomes. These sequences were classified according to their structural and coding features into 1996 TE families. LINE elements (26.5%) and Class Terminal Inverted Repeats (TIR, 13%) were the most prevalent in class I and II, respectively. LTR and TIR orders were dominated by *Gypsy* and *hAT* elements, respectively (Additional File 1: Table S2), as also found in *A. glycines* and *B. tabaci* [14, 20]. Comparisons of these copies within each order of TE and within the clusters defined by REPET show that average identities were generally below 95% (Additional File 1: Fig.S2), suggesting that most superfamilies correspond to ancient invasions. However, a few clusters, corresponding to *Gypsy*, *Bel/pao*, *Tc1-mariner*, and *hAT* elements, showed high degrees of identity (> 95%), suggesting recent expansions of these elements.

### Annotation of protein-coding genes

To improve the quality of gene prediction and to elucidate key biological processes in grape phylloxera, the IAGC fostered a community effort of manual curation, leading to the expert annotation of 4815 genes, or approximately 18.6% of the final gene set (OGS 3.2). All annotation steps and transcription data are stored in AphidBase [21]. This allowed us to perform a phylogenomic study of the phylloxera gene content and specific analyses of functional groups as detailed below.

#### Evolution of gene content and duplication rates

**Fig. 2:**
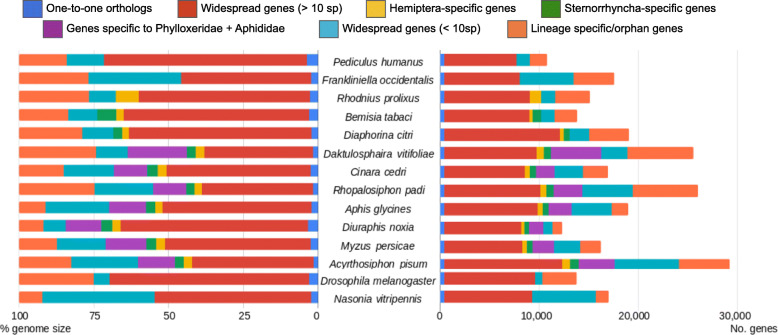
Comparative gene content across insects, with emphasis on Hemiptera. Total number of genes (right) or percentage of genome (left) are indicated

**Fig. 3:**
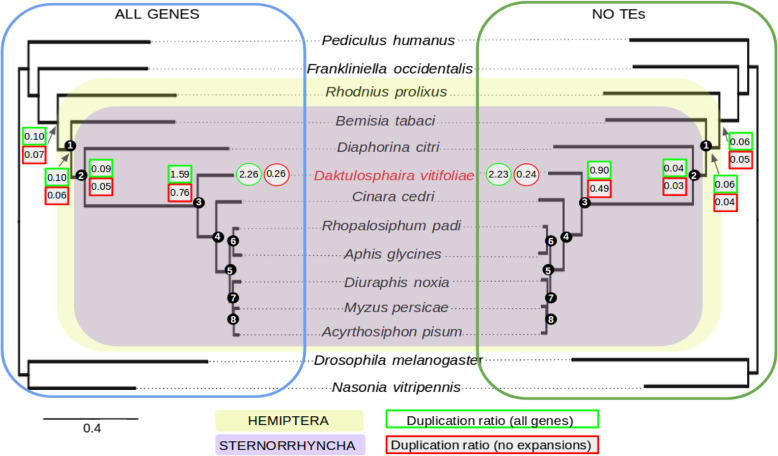
Species tree based on one-to-one orthologs inferred in our data set. The grape phylloxera is indicated in red. All nodes were highly supported in all analyses (> 0.95 SH-like support). Duplication ratios considering all genes and excluding proteins encoded by transposable elements (TE) are plotted in the three most basal nodes of Sternorrhyncha and Hemiptera. The two ratios provided per node, as well as for the phylloxera terminal, correspond to the values resulting from the inclusion (green) or exclusion (red) of gene expansions. Some nodes mentioned in the “Results and discussion” section (marked as 1, 2, and 3) are highlighted

**Fig. 4:**
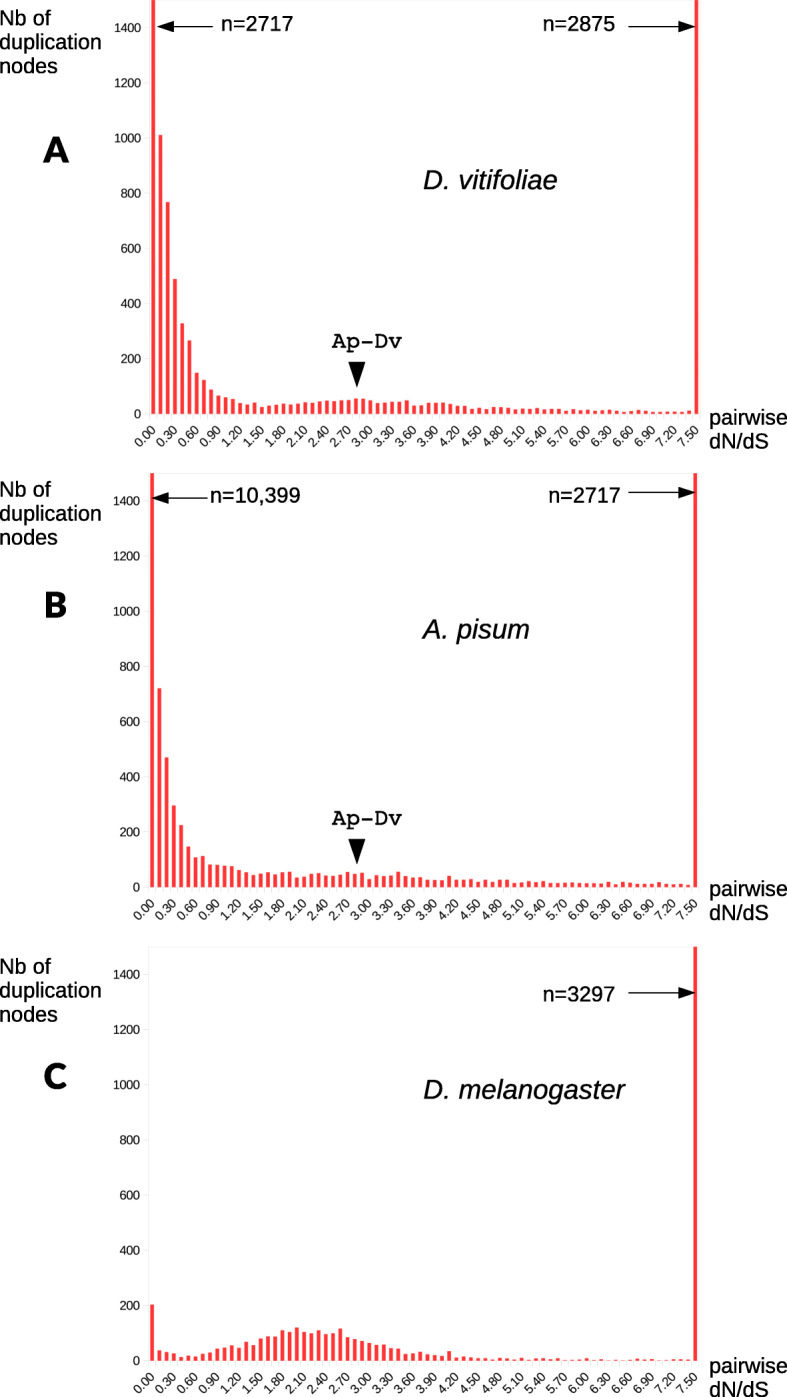
Distribution of synonymous distances among paralogs for grape vine phylloxera (panel **a**, *D. vitifoliae),* pea aphid (panel **b**, *A. pisum*), and fruit fly (panel **c**, *D. melanogaster*). Paralogs were identified as RBH pairs, with an iterative approach allowing to cover both recent duplications (terminal nodes in gene families) and more ancient duplications (internal nodes). For readability, the *y*-axis (number of dS classes) is truncated to 1500 (numbers above that threshold are indicated on the figures). For both *A. pisum* and *D. vitifoliae*, an arrowhead indicates the median dS between orthologs (RBH genes between the two species), dS = 2.83: this metric, a proxy of the age of separation between the two species allows to distinguish duplications that are more recent (left of the arrow, lower dS values) *or more ancient (right of the arrowhead, higher dS) than the speciation event

A comparison of gene content across 14 taxa, including phylloxera, other hemipteran species and several outgroup insect species revealed many widespread genes (red bars in Fig. 2). Lineage-specific and/or orphan genes also were often abundant, particularly in phylloxera and some aphid species (*A. pisum* and *R. padi*) but not all*.* Furthermore, a relatively large number of genes were specific to the Phylloxeridae + Aphididae clade (purple bars, Fig. 2). A total of 6623 genes from the phylloxera genome (25.9% of the total) were phylloxera-specific (i.e., did not have any homologs in the phylogenetic context of our study). These were enriched in GO terms related to sensory perception of taste, protein metabolism, microtubule-based processes, ribosome biogenesis, and G-protein coupled receptor signaling pathway, among others (Additional File 1: Fig.S3). Enriched GO terms in the phylloxera genome, excluding TEs, related to host cell surface receptor binding, hydrogen ion transmembrane transporter activity, odorant binding, and olfactory receptor activity, which suggests that some of the phylloxera-specific gene expansions are involved in sensory perception (Additional File 1: Fig.S4). Among phylloxera-specific genes, 1115 had hits with InterProScan databases, indicating that they may have homologs outside the phylogenetic context of this study. This still leaves 5508 genes in the phylloxera genome with strictly no hit. These results are in line with those found for other aphids. For instance, 4530 genes were inferred as species-specific and/or orphan in *Aphis glycines*, which represents a 23.3% of its genes [22]. We analyzed gene gain and loss patterns across Sternorrhyncha, the hemipteran suborder containing phylloxera; the Sternorrhyncha is defined by its characteristic mouthpart position, adapted for plant sap feeding. Rates and patterns of gene gain and loss varied widely among taxa. The highest level of net gene gain and loss was found for *Diaphorina citri*, with ca. 6500 genes lost in comparison with phylloxera (4442 excluding TEs) (Additional File 1: Fig.S5). The lowest values were obtained for the aphid species *A. pisum*, *M. persicae*, *A. glycines*, and *R. padi*. Interestingly, gene gain and loss were lower at more basal nodes (N1 to N8) than at the tips of the phylogeny (Fig. 3). Our phylome approach for Sternorrhyncha species and outgroups showed a high duplication rate at the base of Phylloxeridae, Adelgidae, and Aphididae (i.e., at node C, where this metric ranged between 0.49 and 1.59 depending on the inclusion of TEs and gene expansions) (Additional File 1: Fig.S6). This, along with our analysis of duplication ages (Fig. 4), indicates an excess of old duplicates predating the diversification of Aphidomorpha. In addition, for relatively recent duplications (nodes for which dS < 1), we found many more duplication events in phylloxera (*n* = 6005 nodes) than in *D. melanogaster* (*n* = 440) (Fig. 3). We found in particular 2717 pairs of paralogs with dS < 0.1, which is 13 times the number found in the *D. melanogaster* genome. An even stronger burst of recent duplications was found for *A. pisum* (10,399 nodes with dS < 0.1, a 51-fold increase compared to *D. melanogaster*) (see [13]). For *A. pisum*, a recent study based on a chromosomal-level assembly showed that duplications in this lineage were dominated by small-scale events, with no signs of larger-scale events [23]. With the goal of understanding the putative role of gene duplicates in the generation of new adaptations in pest species, we explored GO enrichment in the genes duplicated at nodes preceding the diversification of phylloxera (Sternorrhyncha, Psyllidae + Aphidomorpha, and Aphidomorpha). While almost no enrichment was detected in genes duplicated at the nodes respectively preceding Sternorrhyncha and the clade comprising Psyllidae plus Aphidomorpha, genes duplicated at Aphidomorpha were enriched in several functions, including regulation of transcription, protein modification (phosphorylation, protein binding, etc.), neurogenesis, oogenesis, and sensory perception (Additional File 1: Table S3). On top of this, an important part of the recent phylloxera expansions was constituted by lineage-specific genes (most of them, with no GO assigned), which we characterized as putative effectors, as developed below. Altogether, these results suggest that a burst of duplication at the origin of Aphidomorpha, but also more recent species-specific bursts of duplicates, both affecting diverse biological functions, could have contributed to feeding-related adaptations in these lineages.

#### Metabolism and immunity genes

**Fig. 5:**
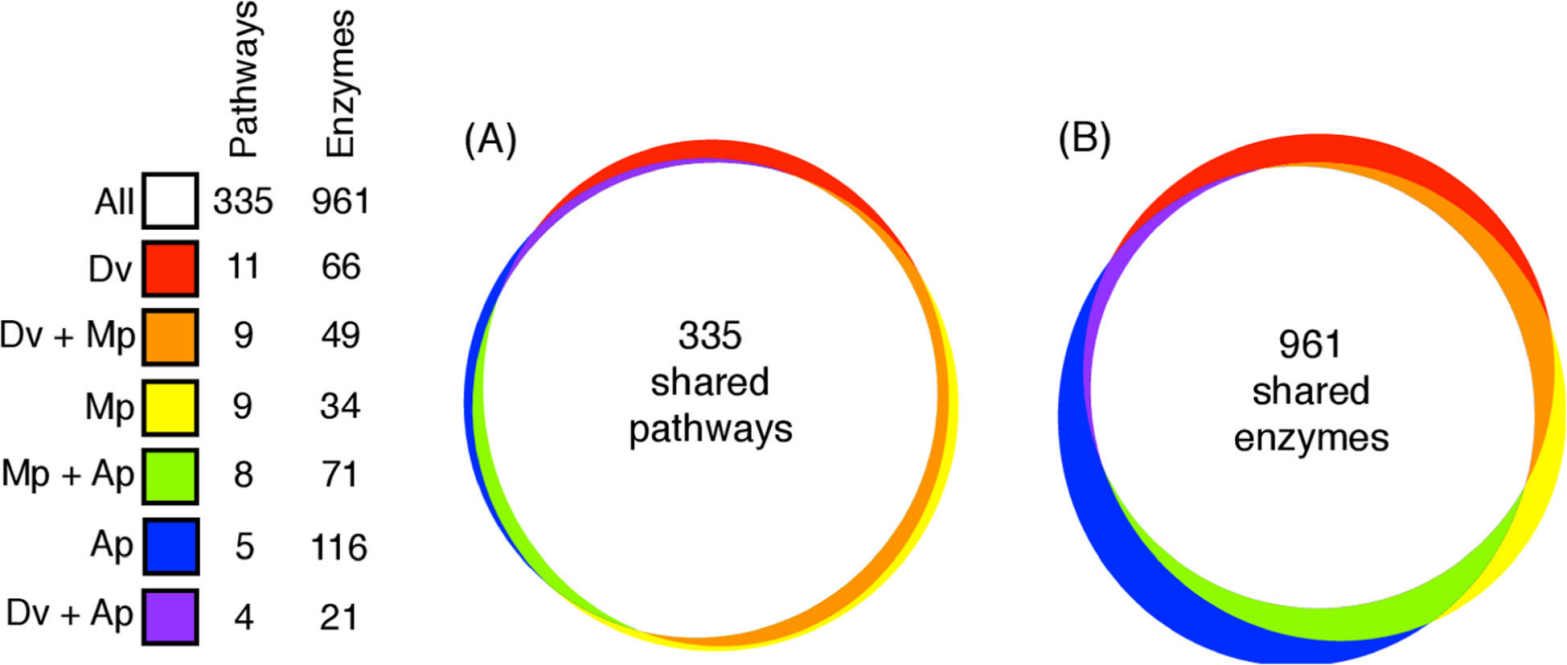
Comparison of the number of pathways and enzymes that are shared among grape phylloxera, *M. persicae* (Mp) and *A. pisum* (Ap). All = all three taxa

**Fig. 6:**
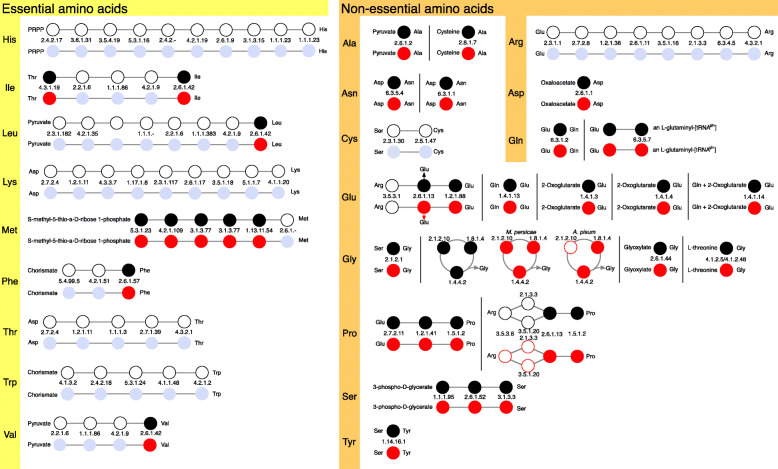
A comparison of amino acid biosynthesis pathways between *D. vitifoliae* (upper or left pathway for each amino acid, with enzymes depicted by black circles) and the aphids *A. pisum* and *M. persicae* (lower or right pathway for each amino acid, with present enzymes depicted by red circles). The presence of an enzyme in a pathway is shown by a filled circle, and the absence by an empty circle. In aphids, the endosymbiotic bacteria *Buchnera aphidicola* is involved in amino acid metabolism: *Buchnera*-produced enzymes are depicted by a filled blue circle. Pathways read from left to right. Where known, enzymes are identified by their EC number

Metabolic pathways were reconstructed combining the CycADS database and a so-called gap-filling procedure (see the “Material and method” section). Gap filling allowed improving annotation by detecting 29 putative additional functions, associated with 39 genes (Additional File 1: Table S4). For example, this includes a candidate gene for phosphopantothenoylcysteine synthetase (DV3025962.1, EC: 6.3.2.5), an enzyme of the coenzyme A biosynthesis pathway, and a candidate gene for nicotinamidase (DV3000063, EC: 3.5.1.19), an enzyme involved in nicotinamide metabolism (Additional File 1: Fig.S7). Thus, the DakviCyc database contains a metabolic network reconstruction of the phylloxera genome. Metabolism was found to be largely conserved between grape phylloxera and the aphids *M. persicae* and *A. pisum* (Fig. 5), as 335 pathways were present in all three species, while we found 11, 9, and 5 unique pathways for *D. vitifoliae*, *M. persicae*, and *A. pisum*, respectively. But 22 pathways were missing in phylloxera compared to the two aphid species (Fig. 5, Additional File 1: Table S5). Finally, the urea cycle (Additional File 1: Fig.S8) was absent from all three species [13, 24]. We identified 1097 different EC numbers with at least one protein in the phylloxera genome (Fig. 5, Additional File 1: Table S5). Of these, 961 appear to be core enzyme functions shared with both *M. persicae* and *A. pisum*. Only 66 were found to be unique to phylloxera, while 34 were found in *M. persicae* and 116 in *A. pisum*. In addition, 221 enzyme functions were found to be missing in grape phylloxera, including 71 shared by *M. persicae* and *A. pisum*. All genes required for amino acid metabolism and found in phylloxera were present in *M. persicae* and *A. pisum* (Fig. 6). Broken metabolic pathways in the two species of aphids are frequently completed by genes encoded by *Buchnera*, the aphid’s primary endosymbiont. However, phylloxera does not have symbionts [25, 26] which would imply that phylloxera cannot synthesize amino acids such as cysteine or arginine (Fig. 6). The bacterium *Pantoea agglomerans* is occasionally found in phylloxera [27], but is not an obligate symbiont, so it probably does not provide missing essential amino acids to this insect. This inability is probably compensated by the specific feeding mode of phylloxerids (modified parenchymal cells which contain essential amino acids) [28–30].

Concerning immunity genes, all genes of the TOLL pathway were found, though some had low similarity to *D. melanogaster* homologs (Additional File 1: Table S6). By contrast, and as previously observed for *A. pisum* and other aphids and the psyllid *D. citri*, several key genes of the IMD pathway present in *D. melanogaster* or other arthropods were missing in phylloxera: *Imd*, *CYLD*, *Fadd*, and *Tab2* (Additional File 1: Table S7, Additional File 1: Fig.S9). Genes encoding *PGRPs* and other antimicrobial peptides were also absent, whereas the JNK pathway, which is connected to IMD in *D. melanogaster*, was complete. Some differences, which are difficult to explain, exist between phylloxera and aphids: *Dredd* and *Kay* are present in phylloxera but not in *A. pisum*, while *Tab2*, absent in the phylloxera genome, is present in *A. pisum*. We also found that only one known transcription factor (TF) regulating the IMD pathway was present in the phylloxera genome instead of the three (*Dif*, *Dorsal*, and *Relish*) in other insects. This phylloxera immune TF matches to the three present in other insect species, and it was not possible to establish clear relationships of orthology between these genes. The lack of an intact IMD pathway in psyllids and aphids has been suggested to relate to their obligate symbiotic associations [31, 32]; however, this hypothesis does not explain the apparent lack of intact IMD pathway in phylloxera, which lacks obligate symbionts. We however note the possibility of divergent genes that would represent a functional pathway as recently shown fo*r R. prolixus* [33].

#### Functional groups that are similar between phylloxera and aphids

**Fig. 7:**
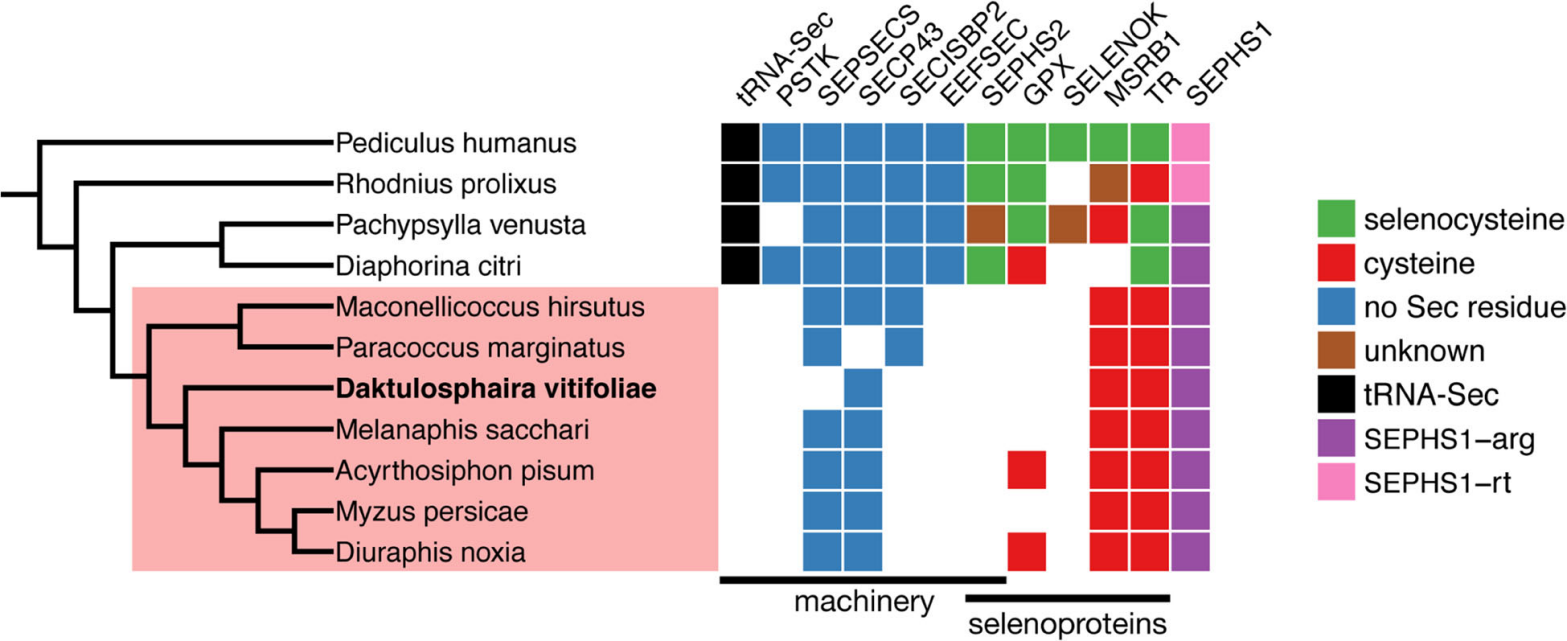
Selenoproteins in Paraneoptera. Species tree annotated with the prediction of selenoproteins and the Sec machinery. The species highlighted in red (Aphidomorpha and Coccoidea) lack selenoproteins and several genes required for their synthesis (the Sec machinery). Each column corresponds to a gene family. Selenoprotein families are colored based on the codon found at the Sec position: selenocysteine in green; cysteine in red; brown indicates incomplete results where the codon at the Sec position was not known. Sec machinery genes are colored in black (tRNA-Sec) or blue (proteins). SEPHS1 is a paralog of SEPHS2, which was found here to have either an arginine codon (SEPHS-arg) or a non-Sec UGA readthrough codon (SEPHS1-rt) at the Sec position

Manual annotation combined with phylogenetic and evolutionary analyses indicates that genes in several functional groups have not changed drastically between Phylloxeridae and Aphididae. This is the case of selenoproteins, which are proteins that include a selenocysteine amino acid residue, this requiring a specific machinery. Although most animals have selenoproteins, several insects including *A. pisum* lack them [13]. We find that grape phylloxera also lacks both selenoproteins and the Sec machinery. Selenoproteins known to be present in other Paraneoptera species were found only as cysteine-containing homologs (MSRB1 and TR) or not found at all (SPS2, GPXx, and SelenoK). Most of the essential factors for selenoprotein synthesis of the Sec machinery, (tRNA-Sec, PSTKpstk, SEPSECSSecS, SECISBP2SBP2, and EEFSECeEFSec and SEPHS2) were absent. Analyzing additional genomes of Paraneoptera allowed to map the selenoprotein extinction event in the common ancestor of scale insects, phylloxera, and aphids (Fig. 7). The gene set underlying structural components of the cuticle is also highly conserved between phylloxera and aphids. A total of 94 unique cuticular proteins (including 11 RR-1 and 61 RR-2) were found in the phylloxera genome. These numbers were similar in aphids, although *A. pisum* showed a larger expansion of the RR-2 protein subfamily (Additional File 1: Table S8). Most RR-1 proteins from phylloxera seem to display 1-to-1 or 1-to-2 orthology relationships with their *A. pisum* and *M. persicae* homologs (Additional File 1: Fig.S10). This reduced complexity signals the absence of specific duplication trends for this protein subfamily (in contrast with the RR-2 subfamily). Concerning the RR-2 subfamily, the main trend was the presence of three clades of high diversification within aphid species, and therefore absent from the phylloxera clade (labeled Post-Dv diversification clusters A, B, and C in Additional File 1: Fig.S11), while a few cases of RR-2 genes from phylloxera phylogenetically close and localized in tandem suggest recent duplications. We found that phylloxera retains standard sets of chitin-metabolizing genes (chitin synthase, chitinases, chitin-binding, chitin deacetylase genes). A single chitin synthase has been detected in all aphid species, and also in phylloxera, a situation correlated with the absence of peritrophic membrane in aphid guts. Lastly, we did not see major differences in the gene complement of the “development” function, even though phylloxera lacks viviparity, a major developmental difference from aphids [34]. This suggests that viviparity in aphids evolved through sub- or neo-functionalization of genes that existed in the common ancestor of the two groups. The developmental gene catalog of phylloxera is nearly complete, with 97 genes annotated (Additional File 1: Table S9). Most of the missing genes were also absent in Aphididae, e.g., *bicoid*, *gurken*, or *oskar*. We found fewer gene duplications in the phylloxera genome than in the *A. pisum* genome (e.g., for *piwi*). Annotations and analyses on microRNAs (Additional File 1: Fig.S12, Additional File 1: Table S10), DNA methylation genes, aquaporins (Additional File 1: Fig.S13), the circadian clock machinery (Additional File 1: Table S11, Fig.S14, Fig.S15), and odorant and gustatory receptors or ligand proteins and detoxification proteins (Additional File 1: Table S12, Table S13, Table S14, Table S15, Fig.S16, Fig.S17, Fig.S18, Fig.S19, Fig.S20) [17, 18, 35–43] are described in the supplementary information document, along with the corresponding methods and results (Additional File 1: Supplementary Methods and Results) [44–76].

#### Extraordinary large expansion of candidate effector genes

**Fig. 8:**
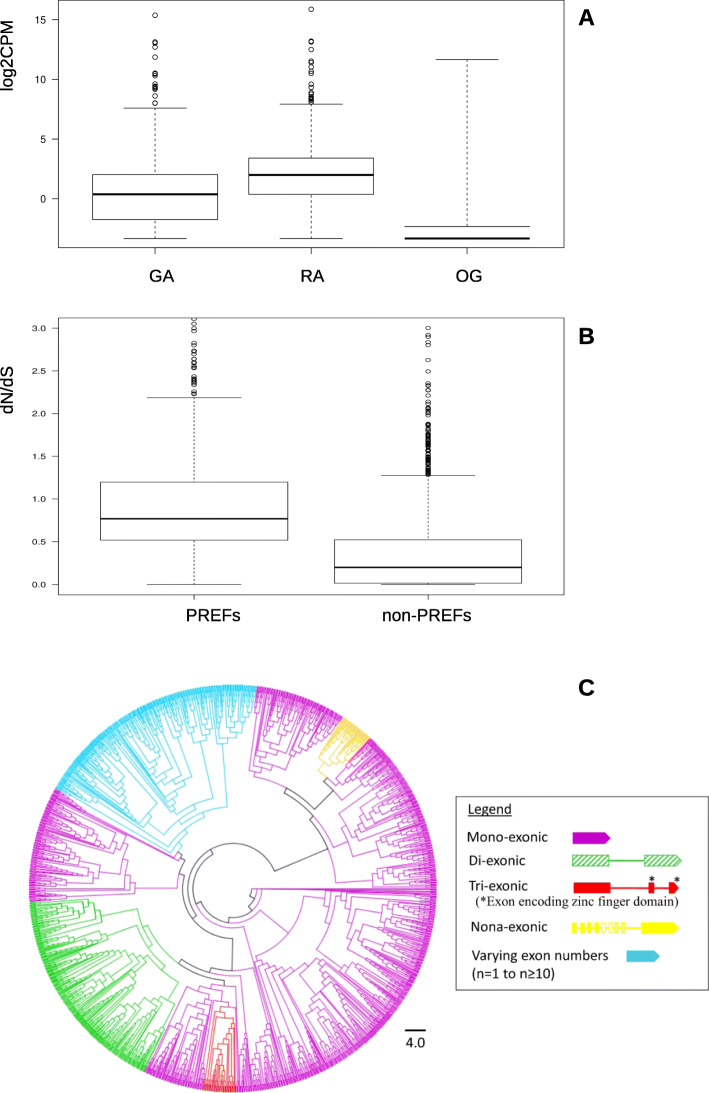
Expression profiles, evolutionary rates, and phylogenetic study of predicted effector genes (PREFs). **a** Expression levels (log2 of counts per million) of PREFs in three life stages: gallicole adult (GA), radicole adult (RA), and egg (OG). **b** dN/dS ratio for PREFs relative to all other coding genes (non-PREFs). **c** Phylogenetic analysis of the largest cluster of effector genes. Exon (box) and intron (line) structure varied as indicated by color with the exception in one clade (blue) where related genes showed variable numbers of exons. Phylogenetic clade colors (left) correspond to gene structure colors shown in legend (right)

We identified over 2700 genes with effector attributes, indicating that a large repertoire of genes underlies nutrition, growth, and defense-related processes during interactions with *Vitis* species. Of these, 419 had domains with known function (Additional File 1: Table S16), yet most genes did not show clear homology to genes in any other organisms (> 86% were no-hit). The three most numerous domains belong to the RING-type zinc finger, ankyrin repeat, and EF-hand domains, which function generally to respectively modulate the ubiquitin-proteasome pathway [77], mediate protein-protein interactions [78], or bind to calcium, e.g., calmodulins, to regulate the cellular calcium signaling pathway [79]. Notably, all pathways play important roles in a vast array of cellular processes and impact nearly every aspect of cellular life including stress response, growth, and development [78, 80]. The largest four groups contained the majority of genes (80% or 2165 of 2741 genes, Additional File 1: Fig.S21), but this was driven by the largest single cluster of 1551 genes (Fig. 8C). This species-specific expansion likely reflects the influence of host specialization as observed for other insect effector genes [81, 82]. Phylogenetic study of this cluster combined with the analysis of exon-intron architecture revealed that most genes lack introns, a feature of genes that function in rapid turnover [83]. Interestingly, some subclades (i) evolved additional (up to and ≥ 10) exons specific to gene clades, (ii) duplicated existing exons to form motif repeats, or (iii) lost exons (Fig. 8c). While the gain of novel exons contributes to the development of new gene functions, exon duplications to form motif repeats help establish stable structures that play versatile roles in many biological processes [84]. A subgroup of genes within the largest cluster contained RING domains (this domain was the most frequent among all domains identified). Thus, genes within this large cluster may mediate protein-protein interactions in part via the ubiquitin-proteasome pathway [77]. This is hypothesized to represent an evolutionary innovation to manipulate plant development, perhaps through molecular mimicry [85–88]. In insects, for example, the Hessian fly delivers hundreds of F-box proteins, a component of SCF-type E3 ubiquitin ligase complex, as effectors likely for insect colonization and gall formation [81], and the green peach aphid (*M. persicae*) and the green rice leafhopper (*Nephotettix cincticeps*) inject EF-hand proteins as calcium binding molecules into host cells during feeding [89, 90]. This hypothesis was also supported by recent evidence of interactions between secretory RING proteins of phylloxera and plant proteins and by the finding of strong downregulation of plant genes related to protein synthesis in *Vitis* galls [91]. Our findings thus suggest that *D. vitifoliae* secretes a pool of effectors to mimic host proteins for plant manipulation.

The great expansion of effector genes is accompanied by a specific pattern of expression restricted to feeding forms, especially on roots (Fig. 8a) and fast evolution, as indicated by high dN/dS values, diversity of exon-intron structures within clusters, and tandem duplication (Fig. 8b, Additional File 1: Fig.S22) [92]. Taken together, these effector gene attributes highlight the complexities that underlie construction of an extended phenotype suggesting a role in coevolution with *Vitis* hosts and mirroring patterns observed to a lesser extent in other insects [81, 93].

### Invasion routes of phylloxera

**Fig. 9:**
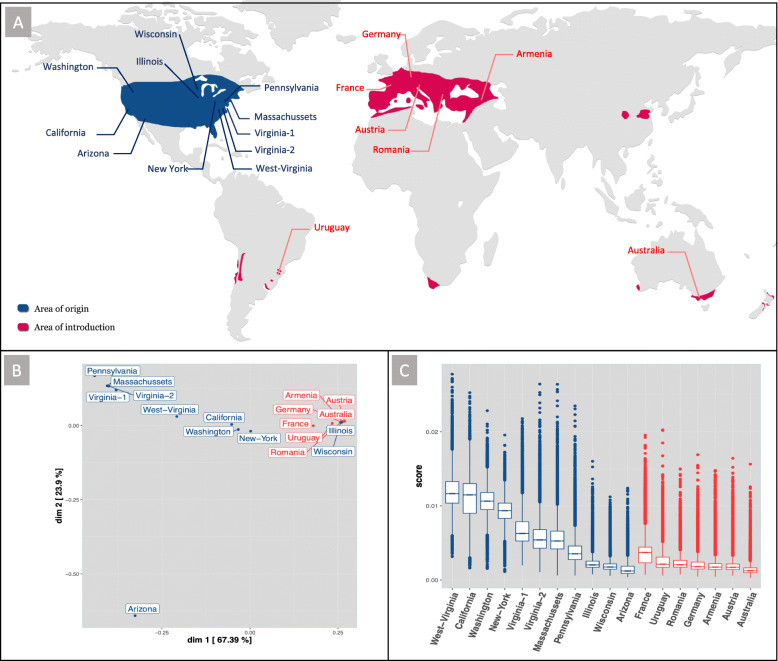
Population genomics comparison of phylloxera populations sampled in the native and invaded range. Insects collected on the same host plant species or cultivar and in the same geographical area (in a single site or in several geographically close sites) were pooled and considered to represent a “population” for genome resequencing. **a** Sampling locations and populations names. **b** Multidimensional scaling (MDS) plot performed on mean FST obtained by pairwise comparisons of native (blue) and introduced (red) phylloxera populations based on 188,980 informative SNPs. **c** Genetic diversity (pi) of native (blue) and introduced (red) phylloxera populations

**Fig. 10:**
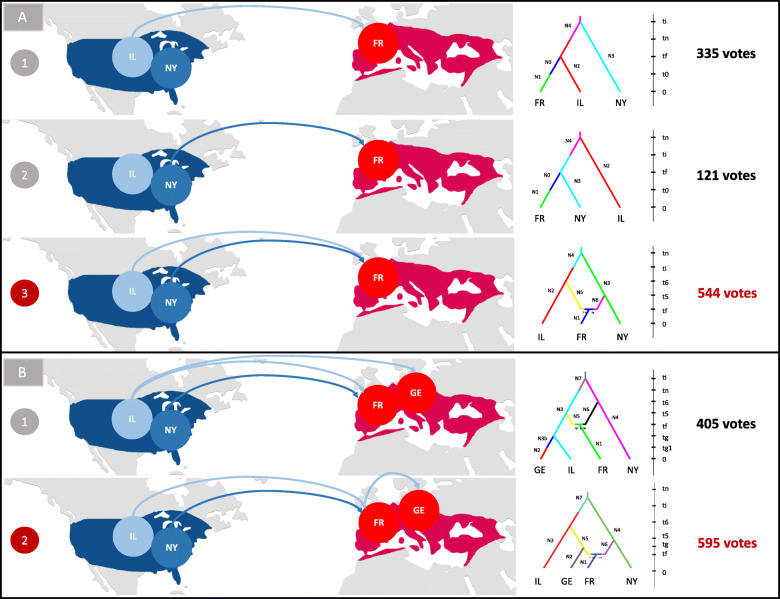
Routes of the phylloxera invasion of Europe inferred from the analysis of genome-wide resequencing data from native and introduced populations. Most likely scenarios of phylloxera introduction into Europe identified by the approximate Bayesian computation (ABC) approach using native populations collected on *Vitis riparia* (New York = NY, Illinois = IL) and introduced populations (France, Germany). **a** Scenarios for the introduction of France, testing a single origin (NY or IL) and admixture. **b** Scenarios for the secondary introduction in Germany, testing an independent introduction from IL versus a common introduction between France and Germany. Detailed legends of the diyABC trees on the right are given in Additional File 1: Table S20

Genome sequencing of pools of insects from several populations of both the native and introduced range was used as a tool to infer the most probable routes of the phylloxera invasion(s) from Northern America to the rest of the world (Fig. 9a) and to compare genetic profiles and variability of the different populations. Samples from the introduced range in Europe clustered together, which is broadly consistent with a single origin for the invasion of the different European countries (Fig. 9b). This European cluster also included two populations from the native range, Wisconsin and Illinois. Therefore, native populations of the upper Mississippi River region, which feed on the wild riverbank grape (*Vitis riparia*), could represent the source of the historic invasion of Europe by phylloxera. This result linking European population and *V. riparia* native populations is consistent with preceding studies using mitochondrial [94] or microsatellite markers [95]. This area, known as French Louisiana in the seventeenth and eighteenth centuries, was under strong French influence and had intense commercial exchanges with France and the rest of Europe into the nineteenth century. At that time, exotic plants were fashionable, and botanists and vine growers had established many personal collections of American vine varieties through the importation of seedlings, cuttings, and rooted plants [7]. Several reports indicate Missouri as the source of resistant rootstocks, suggesting an established grape culture in the Mississippi River region. The French sample however had a distinct profile from the rest of the European populations (Germany, Austria, Romania, Armenia) which were all very tightly clustered with Mississippi valley populations (Wisconsin, Illinois) (Fig. 9b). Using ABC methods, we found that the genetic profile of French populations was best explained as the result of admixture between populations from the Middle West (Wisconsin or Illinois) and from the New York region (Fig. 10a). It is tempting to relate the more diverse genetic profile of French phylloxera population with the historical reports of two independent fronts of colonization in that country, respectively, in Pujaux in the Gard department in 1861 and in Floirac near Bordeaux in 1866 [96] (two sites separated by ~ 430 km). While distinct North American localities may have been sources for the two sites of introduction in France, this hypothesis is difficult to validate without historical phylloxera collections. Also, movements of populations might have erased the possible initial genetic structure resulting from this admixture.

Considering the introduction from the Mississippi valley (represented by Illinois) into the rest of Europe, we tested two scenarios: in the first scenario, colonization of European vineyards would have followed the colonization of France (which served as a bridgehead) by the Illinois population, whereas in the second scenario, there would have been two independent introductions (and two different bottlenecks) from Illinois to France and from Illinois to the rest of Europe. ABC scenarios supported in majority the first scenario (Fig. 10b). Historical reports have documented that the invasion of French septentrional vineyards and central European countries occurred through progressive colonization from sources in South France, which is consistent with this hypothesis [7]. Our data also give new insight into the worldwide invasion of phylloxera, as we found that introduced populations from South America (Uruguay) and Australia were extremely close to European populations. This may result either from an introduction from the same North American source of the European invasion or from a secondary introduction from Europe. The second scenario is likely, as Southern hemisphere vineyards were planted with traditional *V. vinifera* varieties imported from Europe.

The native population from Arizona was found to be highly divergent from all other populations, with a very low level of genetic diversity (Fig. 9c). It is a geographically distant population with insects feeding on a locally distributed host, *Vitis arizonica*. Lund et al. [97] also reported that Arizona populations were markedly different from other North American populations, suggesting that these populations represent a different host race within grape phylloxera or even a distinct species. The estimated divergence between the population from Arizona and other native populations for the *co1* mitochondrial gene (~ 1%) represents a relatively high variation for two races, but could still remain below commonly used thresholds for defining different species [98]. However, relatively low levels of divergence can correspond to a recent event of speciation, a scenario that would fit with the reproductive isolation of this population located on sky islands and likely disconnected from all other populations from the rest of the USA. Surprisingly, Illinois and Wisconsin populations also had very low genetic diversity, similar to what is observed in introduced populations. This suggests a complex story in the native range itself, since genetic bottlenecks could likely explain these patterns (possibly after recolonization or long-term isolation). Our data therefore suggest that some native phylloxera populations had low genetic diversity before they served as a source for the invasion into Europe, suggesting that founder effects [94] are not the sole factor of the limited genetic diversity of introduced populations.

## Conclusions

The genomic resources presented here provide new insights into genome evolution that change our understanding of grape phylloxera interactions. They also open the door to research lines such as the role of the expanded family of effectors in plant feeding, the adaptation of the metabolism in absence of bacterial symbionts, and the influence of host plant specialization on genome architecture. Our results provide a detailed understanding of the genetics underlying invasion and reveal the potential threat to viticulture and native grapes naïve to phylloxera should naturally occurring populations that vary in gene repertoires invade. Given both genotypic diversity and transcriptional plasticity underlie the adaptation of species to novel hosts [24], the genome of grape phylloxera provides the means to understand how populations or even single genotypes adapted to local climates when existing or new populations of phylloxera expanded from North America or Europe to other parts of the world.

## Material and methods

### Biological material for genome sequencing

The isofemale INRA-Pcf7 clone was established from grape phylloxera individuals collected in 2010 at Pineuilh (France) on “Cabernet franc” scions grafted on S04 rootstock (*V. berlandieri x V. riparia*). The clone was maintained in rearing collection at INRAE Bordeaux through parthenogenetic reproduction (controlled chamber at 22 °C, L:16/D:8 and 60% of humidity) on American variety “Harmony” leaves, a Dog-Ridge hybrid of *V. champinii* and accession 1613C (*V. labrusca* x *V. riparia* x *V. vinifera*), and on root pieces of *V. vinifera* “Cabernet sauvignon.”

### Flow cytometry

Two measures were performed independently, using protocols described in [99, 100], respectively. Briefly, measures were performed from the whole body of a female phylloxera INRA Pcf7 clone, using D. *melanogaster* female (1C = 175 Mbp) or alfalfa (*Medicago sativa*) leaf tissue (1C = 206.4 Mbp) as a standard. Nuclei from a mixture of both biological materials (phylloxera vs standard) were prepared and propidium iodide-stained. The relative 2C red fluorescent peak positions of the sample and standard were determined by flow cytometry with the amount of DNA in phylloxera calculated as the ratio of the sample and standard 2C peak means times the 1C amount of DNA of the standard. This was done for *n* = 12 replicates (using *D. melanogaster*) and *n* = 9 replicates (using alfalfa).

### DNA extraction and sequencing

For Illumina sequencing, genomic DNA was extracted from six samples of the Pcf7 clone, each corresponding to approximately 200 individuals, with a mix of adults and larvae. The insects were homogenized using three sterilized glass beads (2 mm diameter) for 30 s at 30 Hz (Tissuelyser, Retsch), and DNA was extracted using DNeasy Blood & Tissue kit (Qiagen Inc., Chatsworth, CA). Between 14 and 25 μg of DNA were obtained from each sample after column elution with 100 μl of 10 mM Tris-HCl-1 mM EDTA, pH 7.8. Quantitation of DNA was performed using DeNovix Fluorescence Assays. Four pair-end and two mate-pair libraries were prepared according to the Illumina manufacturer’s protocol (Additional File 1: Table S17). For PacBio sequencing, four samples, each with ~ 600 adults of the Pcf7 clone, were extracted with a salting-out protocol [101]. Through this protocol, a total of 120 um of long and ultrapure genomic DNA fragments were obtained. Quality was assessed with a NanoDrop (A260/280 ratio between 1.8 and 2.0 and A260/230 ratio ≥ 2.0). Illumina sequencing was performed at the BGI Shenzhen facilities (Shenzen, China) on a HiSeq2500 machine. PacBio was performed at the Genotoul facilities (Toulouse, France) using the SMRT sequencing technology. Illumina pair-end, Illumina mate-pair, and PacBio reads gave a genome sequencing coverage of 147X, 36X, and 58X respectively (Additional File 1: Table S17).

### Reads processing and assembly

We first eliminated adaptors and removed duplicate reads. The remaining sequences were then corrected using the Soap Error Correction (SOAPec_v2.01) tool and assembled using the SOAPdenovo pipeline (version 2.04: released on July 13, 2012) with the options -K 81 (kmer size) and -d 2 (edges cutoff), resulting in 414,258 scaffolds. Scaffolds longer than 500 bp or including a gene annotation (see below) were kept (*n* = 16,380) and scaffolded with PacBio subreads (without correction) using a modified version of SSPACE-LR ver 1.1 [102], with the option “-s 1 -a 250”. Finally, the gaps of this last version were filled with Illumina reads using GapFiller [103].

### Automatic annotation and manual curation

Gene predictions were generated using MAKER2 [104]. Within MAKER2, a first gene set was predicted by similarity to known proteins, or contigs of RNA-Seq (see below). This gene set was used thereafter for training both Augustus [105] and SNAP [106], in two steps, using results from an initial training to retrain again the software. Transcriptomic evidence came from two previous RNA-Seq projects [107, 108], which included whole bodies of leaf-galling adults (gallicoles), whole bodies of root-feeding adults (radicicoles), and eggs from radicicoles. Proteomic evidence came from SwissProt (release 2016_10) and a protein set from various hemipteran species, including *A. pisum* (NCBI), *M. persicae* Clones G006 and O (AphidBase), *D. noxia* (NCBI), *Cimex lectularius* (NCBI), and *Rhodnius prolixus* (EnsEMBL). An Apollo [59] server was set up to allow manual curation of a set of genes from the automatic annotation. As many as 4815 genes were curated and checked based on guidelines defined by BIPAA [https://bipaa.genouest.org/is/how-to-annotate-a-genome/]. Curated genes were merged with the automatic annotation using a custom script [https://github.com/abretaud/ogs-tools/tree/master/ogs_merge]. Putative functions of predicted proteins by the above pipeline were identified with blastp (v2.6.0) against Genbank NR (non-redundant GenBank CDS translations+PDB+SwissProt+PIR+PRF, release 09/2017), and interproscan v5.13-52.0 against Interpro. Associated GO terms were collected from blast NR and interproscan results with blast2GO (v2.5). Transmembrane domain signal peptides were identified by tmhmm v2.0c and signalP (euk v4.1), respectively. All genome resources and the Apollo server were made available online on BIPAA, within the AphidBase section [http://bipaa.genouest.org/is/aphidbase/daktulosphaira_vitifoliae/] [21]. This system was rolled out using different projects from the GMOD tool suite (JBrowse [109], Chado [110], Tripal [111]) and developments from the Galaxy Genome Annotation project [https://galaxy-genome-annotation.github.io/] [112].

### Detection of contaminant scaffolds

A screening of contaminants was performed on scaffolds (blastp of the predicted proteins to nr), which allowed to eliminate 3 scaffolds identified as bacterial. We also used BLOB [113] which screens viral, bacterial, and eukaryotic contaminants based on GC content and similarity. We identified a very small number of potential residual contaminants: they totalled only 1.352 Mbases in 92 scaffolds (0.5% of the assembly size), suggesting that contamination would at best be marginal.

### Characterization of the mitochondrial genome

The mitochondrial genome sequence from the grape phylloxera was found during genome assembly. The initial mitochondrial scaffold was 15,613 bp in length, and inspection of the predicted gene sequences revealed a frameshift within the *nad5* sequence. Closer inspection showed a possible insertion of 45 T nucleotides within *nad5*. PacBio reads were mapped to this region, revealing that the insertion was likely due to a sequencing or assembly error. This insertion was removed, resulting in an intact *nad5* gene sequence. The final assembled scaffold is 15,568 bp in length and has a GC content of 15.6%. A gene prediction analysis was performed on this scaffold using MITOS [114] and ARWEN v1.2 [115].

### Horizontal gene transfers

To determine if genes for carotenoid biosynthesis were present in the phylloxera genome, we used genes of this pathway previously characterized in *A. pisum* as query sequences for blastp searches on the predicted proteins of the phylloxera genome. The *A. pisum* genome also contains genes from bacterial sources [17, 18], and, again using the *A. pisum* sequences as queries, we performed blastp searches on protein databases for the genomes of *D. vitifoliae*, *A. pisum*, *Myzus persicae*, *Diuraphis noxia*, *Aphis glycines*, *Rhopalosiphum padi*, *Diaphorina citri* (Psyllidae), *Pachypsylla venusta* (Psyllidae), and *Bemisia tabaci* (Aleyrodidae). A blastp search was also conducted in the NCBI non-redundant protein sequence database, in order to identify other species where these genes might be present. The alignments were made using MAFFT v7.313 using default parameters [47]. Phylogenetic trees were constructed from sequences retrieved from blastp searches, using RAxML under the PROTCATJTTF model, with 100 bootstrap replicates.

### Repetitive DNA

Transposable elements (TEs) were identified and annotated using the REPET package v2.2 [116, 117]. Manual inspection was performed to confirm TE orders, clusters, and families. The level of identity between a fragment and its reference TE/repeat consensus was used to estimate ages of TE expansions.

### Annotation of protein-coding genes

#### Gene expansions

##### Phylome reconstruction

The phylome (i.e., the complete collection of phylogenetic trees for each gene in a genome) of grape phylloxera was reconstructed to obtain a dynamic view of gene family expansion within this genome. We included nine other fully sequenced genomes of Hemiptera based on their phylogenetic position: *A. pisum* (Harris, 1776) (Sternorrhyncha, Aphididae), *M. persicae* (Sulzer, 1776) (Sternorrhyncha, Aphididae), *D. noxia* (Kurdjumov, 1913) (Sternorrhyncha, Aphididae), *C. cedri* (Curtis, 1835) (Sternorrhyncha, Aphididae), *A. glycines* Matsumara, 1917 (Sternorrhyncha, Aphididae), *R. padi* (Stal, Linnaeus, 1758) (Sternorrhyncha, Aphididae), *D. citri* Kuwayama, 1908 (Sternorrhyncha, Psylloidea), *B. tabaci* (Gennadius, 1889) (Sternorrhyncha, Aleyrodoidea), and the true bug *Rhodnius prolixus* (Stål, 1859) (Heteroptera, Reduviidae). As outgroups, we selected four insect taxa: *D. melanogaster* Meigen, 1830 (Diptera, Drosophilidae), *Nasonia vitripennis* (Ashmead, 1904) (Hymenoptera, Pteromalidae), *Frankliniella occidentalis* (Pergande, 1895) (Thysanoptera, Thripidae), and *Pediculus humanus* (Linnaeus, 1758) (Phthiraptera, Pediculidae). Genome versions are indicated in Additional File 1: Table S18. Phylomes were reconstructed using the PhylomeDB pipeline [118]. For each protein encoded in the grape phylloxera genome (25,567 annotated proteins, Official Gene Set version 3.2) (http://bipaa.genouest.org/sp/daktulosphaira_vitifoliae/), we performed a blastp search against the custom proteome database built from the genomes listed above, which included a total of 252,530 proteins. Results were filtered using an *e* value of 1e−05 and a minimum of 50% overlap between the query and the hit sequences. Multiple sequence alignments were reconstructed in forward and in reverse [119] using three different programs: MUSCLE v3.8 [120], MAFFT v6.712b [61], and Kalign v2.04 [121]. The resulting alignments were then combined using M-COFFEE v10.00.r1607 [122]. A trimming step was performed using trimAl v1.3 [48] (consistency-score cutoff 0.1667, gap-score cutoff 0.9). The best fitting model was selected by reconstructing neighbor joining trees as implemented in BioNJ [123] using seven different models (JTT, LG, WAG, Blosum62, MtREV, VT, and Dayhoff). The best model in terms of likelihood as selected by the Akaike Information Criterion (AIC) [124] was chosen for tree reconstruction. Trees were reconstructed using PhyML v20120412 [62]. Four rate categories were used, and invariant positions were inferred from the data. Branch support was computed using an aLRT (approximate likelihood ratio test) based on a chi-square distribution. Resulting trees and alignments are stored in phylomeDB 4.0 [118] (http://phylomedb.org), with the phylomeID 196. A species-overlap algorithm, as implemented in ETE v3.0 [125], was used to infer orthology and paralogy relationships from the phylogenetic trees reconstructed in the phylome. The algorithm traverses the tree and calls speciation or duplication events at internal nodes based on the presence of common species at both daughter partitions defined by the node. Gene gains and losses were calculated on this basis. Duplication ratios per node were calculated by dividing the number of duplications observed in each node by the total number of gene trees containing that node: theoretically, a value of 0 would indicate no duplication, a value of 1 an average of one duplication per gene in the genome, and > 1 an average of more than 1 duplication per gene and node.

##### Species tree reconstruction

The species tree was built using one-to-one orthologs present in all 14 included species, with a final alignment of 409 genes and 245,463 concatenated amino acid positions. To ensure a congruent phylogenetic hypothesis under different models, a series of approaches were followed to infer the species tree. First, an approximately maximum-likelihood tree was reconstructed with FastTree v. 2.1 [126] under the LG [127] model of amino acid evolution. Second, a supertree was reconstructed using DupTree [128] based on all the trees reconstructed in the phylome. Both phylogenies were congruent.

##### Removal of proteins from transposable elements

In order to disentangle the effect of transposable elements (TEs) and of other factors, we removed all genes annotated as proteins encoded by TEs, prior to the inference of gene expansions, GO term enrichment and gene gains, losses, and duplications.

##### Detection of expanded protein families

For each gene tree, we selected the nodes that contained only phylloxera sequences with ETE v3.0 [125]. Nodes with more than 5 sequences were counted as expansions. Overlapping expansions (i.e., partial gene trees with terminals in common) were fused when they shared more than 20% of their members.

##### Gene annotation and scrutiny of putative phylloxera-specific genes

In addition to the automatic and manual annotation performed on the phylloxera Official Gene Set (OGS) (http://bipaa.genouest.org/sp/daktulosphaira_vitifoliae/), all genes in the phylloxera genome were functionally annotated with InterProScan v.5.19 [129]. Gene Ontology [130] annotations and PFAM [65] motifs were assigned to these genes as well. All genes that did not show any BLAST hits during the all-by-all comparison (see the “Phylome reconstruction” section) were interpreted as putative phylloxera-specific genes. These genes were further scrutinized through functional annotations with InterProScan v.5.19 [129] as well.

##### GO term enrichment

FatiGO [131] was used to check for enrichment in GO terms between the phylloxera genes and the rest of the database (i.e., the sum of the genes belonging to the other species included in the phylome). Sets of enriched GO terms were summarized and visualized in REVIGO [132]. GO enrichment was explored for phylloxera-specific genes, as well as for genes duplicated in each of the nodes to evaluate potential specific adaptation at different time points of the evolution of this species and group.

##### Synonymous distance-based assessment of duplication ages

To remove potentially spurious gene models from the official gene set, we first used a filtering step, eliminating genes which had very weak support: these were defined as genes with no manual annotation, no hit to the nr database of GenBank, and very low RNA-Seq support (< 0.5 CPM for the average of expression counts between radicicoles, gallicoles, and eggs). This left us with *n* = 21,863 genes (a filtering of nearly 4000 genes). To evaluate synonymous distances (dS) among paralogs, we used a Reciprocal Best Hit approach (RBH) by blasting gene collections against themselves, determining pairs of genes that matched the RBH criteria. Doing this in just one step would lead us to focus on terminal branches in expanded gene families, neglecting deeper nodes and thus missing the ancient dynamics in the history of duplications. To account for this, we applied an approach similar to that used in [133]: after a first round of RBH identification, a member of each RBH pair was tagged for elimination (we chose the shortest sequence, or randomly selected one of the genes in case of equal lengths). We then re-started the RBH identification, allowing to gradually reach deeper nodes in gene families. The process was reiterated 10 times, as the number of duplications decreased sharply in the last runs. Each RBH pair of genes in the different runs (representing a node in gene families) was used for a pairwise estimation of synonymous distance. For this, the protein sequences were aligned; this alignment was then reported on the nucleotide sequence and cleaned using GBlocks [134]; this step eliminated poorly aligned regions, giving a conservative estimate of the distances among copies. Finally, dS was estimated using Codeml (PAML software [135]). For comparison, we applied the same procedure to the *A. pisum* genome (using the NCBI update prediction, *n* = 27,986 genes) and for *D. melanogaster* (using the r6.21 annotation, and selecting the longest alternative transcript of each gene, *n* = 13,931 gene sequences).

#### Metabolism

##### CycADS annotation and DakviCyc database generation

We used the Cyc Annotation Database System (CycADS [136]), an automated annotation management system, to integrate protein annotations from different sources into a Cyc metabolic network. Using the CycADS pipeline, proteins were annotated using Blast2GO [137], InterProScan [129], KAAS [138], PRIAM [139], and PhylomeDB [118] to obtain EC and GO numbers. These data were processed in the CycADS SQL database and automatically extracted to generate appropriate input files to build or update BioCyc databases [140] using the Pathway Tools software [141]. The DakviCyc database, representing the metabolic protein-coding genes of phylloxera, was thus generated and is now included in the ArthropodaCyc database, a collection of arthropod metabolic network databases [142] (http://arthropodacyc.cycadsys.org/).

##### Metabolic pathway gap filling

Metabolic reconstructions from the ArthropodaCyc databases for *D. vitifoliae*, *A. pisum*, and *M. persicae* (clone G006) were exported in the SBML format and imported into the PSAMM software [143]. First, metabolic pathway gaps were identified using the “*gapcheck*” function, which reports a list of all metabolites not produced in the metabolic network. Then, the objective function was defined for each non-producing metabolite, and a gap-filling procedure was performed for each objective function through individual rounds of simulations using the PSAMM implementation of the *fastgapfill* algorithm [144]. In the gap-filling step, results from *A. pisum* and *M. persicae* were used as candidates for identifying potentially missing annotations. Following the gap-filling predictions, candidate missing genes were identified through the identification of homologs to annotated genes in *A. pisum* and *M. persicae*. This was achieved with manual curations using evidence from blast alignments, Pfam protein domain identifications [65], phylomeDB [118], transcriptomic support of gene expression, and literature review. Two rounds of annotation were performed with the above procedure, and predictions in the DakviCyc database were updated through these iterations. External links to resources that include the comprehensive enzyme information system: BRENDA (https://www.brenda-enzymes.org/), InterPro [129], KEGG orthology (https://www.genome.jp/kegg/), PhylomeDB [118], and crosslinks to the AphidBase [21] genome browser were added for all predicted genes.

### Immunity genes

Immune genes were annotated using bidirectional blastp analyses. We first used the phylloxera gene set to identify proteins with similarity to genes of the IMD and TOLL pathways. These putative phylloxera proteins were then blasted against *D. melanogaster* reference proteins. This approach was then extended to a complete collection of *D. melanogaster* immune genes. For reciprocal best hits (RBH) between phylloxera and *D. melanogaster*, the *D. melanogaster* annotation was directly transferred to phylloxera. In other cases (non-RBH relationship), a manual curation was performed, using the genomic information for arthropods with well-annotated immune pathways (*Nasonia vitripennis*, *Plautia stali*, *Rhodnius prolixus*, *Tribolium castaneum*) or for other aphid genomes (*A. pisum and M. persicae*) available in Genbank, ArthropodaCyc, and ImmunoDB [145] databases.

### Cuticular proteins

To determine the full set of genes coding for cuticular proteins (CPs) (including cuticular proteins with R&R motif defined as CPR proteins [146]), we searched CPs among the initial prediction by using the CutProtFam annotation website [147] (http://aias.biol.uoa.gr/CutProtFam-Pred/), with standard settings. Candidate genes were then fully manually curated on AphidBase through Apollo. Phylogenetic analyses were performed using the updated protein sequences of sets of RR-1 or RR-2 genes of *M. persicae* [24], *A. pisum* [148], and *D. noxia* [149]. RR-1 and RR-2 sub-groups were treated separately. For RR-1 proteins, signal peptides were predicted using ExPASy tools (http://www.expasy.org/tools/) and removed; then phylogenetic analyses were conducted on the mature sequences. For RR-2 proteins, only the extended 69 amino acids RR domain (pfam00379) was used for phylogenetic analysis, the rest of the sequences being too divergent to align. Alignments were made with Clustal Omega [150], and phylogenetic analyses were made using the Phylogeny.fr platform [151] where alignments were cleaned with Gblocks and a maximum likelihood method as implemented in the PhyML program was used to infer a phylogenetic tree.

### Selenoproteins

Selenoproteins contain the non-canonical amino acid selenocysteine (Sec), known as the 21st amino acid. Sec is encoded by a UGA codon, normally a stop codon, and is inserted through a recording mechanism that requires a dedicated set of factors known as the Sec machinery [152]. Selenoproteins exist in different domains of life and are widespread in Metazoa, but appear to be lacking in some insect species [153] including the pea aphid [13], two Astigmata (non-insect arthropods) species [154], and plant parasitic nematodes [155]. To search for selenoproteins and the Sec machinery, the genome of grape phylloxera was analyzed with Selenoprofiles [156] and Secmarker [154].

### Effectors

To identify genes underlying effector proteins active when grape phylloxera interacts with *Vitis* host plants, we modified a bioinformatics pipeline from [157]. This pipeline was designed based on four features of effectors: (1) secretory, (2) small-sized (≤ 500 amino acids, and this only applied on the initial screening), (3) herbivore-only, and (4) gene-duplicating [157, 158]. Testing of this pipeline on the genome dataset of the Hessian fly (*Mayetiola destructor*), a plant manipulating herbivore [81], showed that 95% of the predicted effector genes matched (blastp *e* value < 1e−5) the salivary gland-derived Hessian fly effector genes. We therefore screened the 24,585 automated gene models (OGS3.0_20161223_proteins) and predicted a first set of 354 effector genes that classified (using OrthoMCL) into 78 clusters according to sequence similarity. We then performed manual annotation on each of these clusters to (1) correct gene models based on the transcript data from gallicole, radicicole, and egg samples [107] integrated into Apollo and sequence similarity to other members of the same cluster and (2) recover gene models, through tblastn searches, that were not included in the automated annotation and prediction of OGS3.0_20161223_proteins because of mis-prediction. Using this automated gene model-based (AGMB) approach (note that it also identified effector candidates that were absent of automated gene models but shared sequence similarity to the ones predicted from the automated gene model collections), and eliminating our sequence size limit to include proteins > 500AA, we predicted 1766 effector candidates from the genome of *D. vitifoliae*. While conducting manual annotation on the genome, we detected a number of putative genes which had particular characteristics: (1) absence of automatic annotation (i.e., no gene model was predicted), (2) presence of an ORF usually encoding more than 200 amino acids and corresponding to a monoexonic structure, and (3) clear RNA-Seq support, in particular in the radicicole samples. The two former points suggested that these ORFs represented bona fide genes, with a particular intron-less structure. Such pattern is usually penalized in gene prediction tools for Eukaryotes, which could explain the absence from the automatic gene model prediction. Additional traits of these genes suggested that they encoded effectors because of (1) the presence of a secretory signal peptide in the N-terminus; (2) clusters of similar gene copies, indicative of tandem gene duplication; and (3) some sequence similarity to the putative effectors predicted using the AGMB approach described above. To generalize the search of similar genes, we performed tblastn searches to the genome and annotated matching regions which shared the above patterns. Because we usually found different hits in each search, but with a relatively low amino acid identity (as low as 20%), it appeared that the grape phylloxera genome encodes highly expanded gene families characterized by high evolutionary rates. To ensure that we collected the most complete collection of genes, the tblastn searches were performed iteratively, each time using the collection of manually annotated monoexonic effector candidates as a query data set, then annotating the new hits, and repeating this process until no new hits were detected. Some of the effector candidates identified using this non-automated gene model-based (NAGMB) approach overlapped with those identified through the AGMB pipeline and therefore were combined with the latter resulting into a total number of 2741 manually annotated predicted effectors (PREFs) in the phylloxera genome. Genes were clustered using SiLiX. Because numerous PREFs appeared unique, lacking sequence homology to other PREFs, and comparisons were based within species rather than among species, the final clusters were determined through an iterative process in SiLiX. As overlap among sequences increased to 60%, very few new clusters were formed. Similarly, as identity decreased down to 20%, the number of clusters reached a minimum. Thus, 60% overlap and 20% identity were designated as conservative thresholds per parameters defined in SiLiX. As PREF function is validated through further study, these thresholds may change to best organize clusters without breaking up families of known function predicted from sequence motifs. Phylogenetic analyses of the largest orthogroup (cluster3, *n* = 1551 PREFs) were performed following the protocol described by [30] with modifications. Briefly, the deduced protein sequences were aligned using MAFFT (v7.271) [47] with “auto” setting and the alignments were trimmed using TRIMAL(v1.4) based on a gap threshold of 0.25. One PREF (DV3018723) was removed because its sequence is composed only by gaps after trimming, leaving a total of 1550 PREFs with 375 amino acids each (including gaps) for phylogenetic tree construction. Lastly, these aligned sequences were run on PhyML (v3.0) with the value of approximate Likelihood-Ratio Test (aLRT) for branches set as “-1.” To evaluate selective pressures acting on PREFs (comparing the different orthogroups, and comparing PREFs and non-PREFs), we estimated evolutionary rates for the most recent duplication events in the genome. These events were pointed by determining reciprocal best hits (RBH) and by estimating the pairwise non-synonymous to synonymous ratio (dN/dS) for each pair of sequences found to be RBH. For that, we aligned sequences, trimmed the alignments (with Gblocks), and evaluated rates with codeml (PAML). For RBH detection, we included all manually curated genes (including PREFs); among the other genes, we eliminated gene models with very low support (genes with no hit and a very low RNA-Seq support, i.e., < 0.5 counts per million reads in radicicoles, gallicoles, and eggs data sets). This filtering was intended to remove noise and potentially inflated rate estimates that might occur for spurious gene models (the resulting data set comprised 23,961 genes).

### Genome resequencing of phylloxera populations and invasion route inference

Phylloxera individuals were collected from both native and introduced areas. All samples consisted of gall-feeding adult insects except for two American populations (California, Washington) that were sampled as root-feeding insects. Insects collected in the same geographical area (in a single site or in several geographically close sites) and on the same host plant species or cultivar were pooled and considered to represent one population for genome resequencing. In the native area, samples were collected either on cultivated grapevines or on wild *Vitis* species: *Vitis arizonica* (Arizona), *Vitis labrusca* (Massachusetts), *Vitis aestivalis* (West Virginia), *Vitis vulpina* (Pennsylvania, Virginia1), *Vitis riparia* (Wisconsin, Illinois, New York), interspecific hybrid Chambourcin and Concord (Virginia2 and Washington, respectively), rootstocks 1103P (California). Populations from introduced areas (France, Germany, Hungary, Austria, Romania, Armenia, Uruguay, Australia) were collected from galls on leaves of *Vitis vinifera cv.* Details on this sampling are presented in Additional File 1: Table S19. For each pool, which comprised between 30 and 100 individuals (adult insects), a DNA library was prepared with the TruSeq Nano Illumina kit, and sequenced on one lane of an Illumina HiSeq3000 sequencing machine at the Genotoul platform (reaching a genome coverage of ~ 60X for each pool). The reads (paired-end 2 × 150 bp) were mapped on the genome reference with BWA mem v0.7.10, with default parameters. Only primary alignments of properly paired reads were kept using samtools, and PCR duplicates were removed using Picard tools (https://github.com/broadinstitute/picard). Each pileup file was then subsampled with Popoolation2/subsample-pileup.pl [159] in order to reach a coverage of 15 at each site, and individual population genetic statistics (diversity, mutation rate, and Tajima’s *D*) were calculated with Popoolation2/Variancesliding.pl. The counts of major alleles for each population and for each position were calculated from the subsamples and used as entry for the PCA (FactomineR). We used popoolation2/FST_sliding to estimate pairwise FST after the synchronization of the pileup files with Popoolation2/mpileup2sync.jar, extraction of polymorphic sites (minimal count of the minor allele over all the samples = 4, and coverage at each site and each sample > 10) and subsampling (as above). The average of FST pairwise were computed and used for generating a distance matrix distance for the MDS plot (done with R/ggplot2).

In order to test various demographic scenarios for the introduction of phylloxera in Europe, we used diyABC, and abcRF [160]. We randomly selected 10,000 polymorphic SNPs and 100 monomorphic SNPs in 5 populations (France, Germany, Illinois, Wisconsin, and New York) and generated individual data (respectively 200, 140, 170, 120, and 200 individuals) based on the observed allelic frequencies at each site. With diyABC, we extracted summary statistics (with respect to the distributions of the diversity, FST, and Nei’s distances) for more than 10,000 simulations by scenarios and used abcRF to compare simulations results with summary statistics from our observed genotypes in order to choose the most realistic model (i.e., those with more votes among 1000 trees in the random forest). We first compared the demographic scenarios in the native area, with and without admixture, selecting the best model, then introduced sequentially the French and German populations (representing the two genetic profiles of phylloxera populations found in Europe)—for detailed statistics, see Additional File 1: Table S20.

## Supplementary information

**Additional file 1: Figures. S1-S22, Table S1-S20, Methods and Results. Figure S1.** Mitochondrial genome view of grape phylloxera. **Figure S2.** Proportion of transposable elements (TE) in the genome. **Figure S3.** GO terms of phylloxera-specific genes. **Figure S4.** Enriched GO terms in the phylloxera genome with and without TEs. **Figure S5.** Gene gain/loss at different nodes or branches. **Figure S6.** Species phylogenetic tree based on insect genomes and the transcriptomes of *Planoccoccus citri* and *Adelges tsugae*. **Figure S7.** Diagram of the gap-filling and annotation process. **Figure S8.** Urea cycle in *D. vitifoliae* and *A. pisum*. **Figure S9.** IMD immune pathway in *D. vitifoliae.***Figure S10.** Phylogenetic tree of RR-1 cuticular proteins*.***Figure S11.** Phylogenetic tree of RR-2 cuticular proteins*.***Figure S12.** Comparison of miRNAs in *D. vitifoliae* and other insect genomes. **Figure S13.** Phylogenetic tree of aquaporin protein sequences. **Figure S14.** Comparison of the phylloxera PER protein with other insects. **Figure S15.** Amino acid alignment of PTTH amino acid sequences. **Figure S16.** Phylogeny of hemipteran ORs. **Figure S17.** Phylogeny of hemipteran GRs. **Figure S18.** Phylogenetic analysis of OBPs. **Figure S19.** Phylogenetic analysis of CSPs. **Figure S20.** Phylogenetic analysis of NPC2s. **Figure S21.** Distribution of cluster sizes of putative effectors. **Figure S22.** Physical distribution of the three largest clusters of effectors. **Table S1.** Genes of bacterial and fungal origin. **Table S2.** Statistics on TEs. **Table S3.** GO enrichment of genes duplicated at different ancestral nodes. **Table S4.** Metabolic gaps in the D. vitifoliae reaction network. **Table S5.** Functional annotation of metabolic genes. **Table S6.** Genes of the TOLL pathway. **Table S7.** Genes of the IMD pathway. **Table S8.** Statistics on cuticular proteins. **Table S9.** Developmental genes in *D. vitifoliae* and *A. pisum*. **Table S10.** miRNAs. **Table S11.** Clock-related genes. **Table S12.** List of ORs and GRs. **Table S13.** Number of OBPs, CSPs and NPC2s. **Table S14.** List of Cytochromes P450. **Table S15.** List of genes involved in detoxification. **Table S16.** Effector genes with predicted domains and their corresponding functions. **Table S17.** Statistics on sequence reads and SRA accessions used for the reference genome. **Table S18.** List of species used to study gene expansions. **Table S19.** Sampling sites and SRA used for population genomics analyses. **Table S20.** Prior distribution of parameters used for ABC modeling of invasion routes.

## Data Availability

All data generated or analyzed during this study are included in this published article and its supplementary information files. Reads produced in this study are available at the NCBI Short Read Archive (SRA) under accession PRJNA588186 [161] for reads used for the reference genome, and under accession PRJNA588387 [162] for reads used for the population genomics study. Other datasets (assembled sequence, official gene sets, microRNAs, mitochondrial genome) are available at the Aphidbase repository [163].
