# Correction to: The genome sequence of the grape phylloxera provides insights into the evolution, adaptation, and invasion routes of an iconic pest

**DOI:** 10.1186/s12915-020-00864-7

**Published:** 2020-09-11

**Authors:** Claude Rispe, Fabrice Legeai, Paul D. Nabity, Rosa Fernández, Arinder K. Arora, Patrice Baa-Puyoulet, Celeste R. Banfill, Leticia Bao, Miquel Barberà, Maryem Bouallègue, Anthony Bretaudeau, Jennifer A. Brisson, Federica Calevro, Pierre Capy, Olivier Catrice, Thomas Chertemps, Carole Couture, Laurent Delière, Angela E. Douglas, Keith Dufault-Thompson, Paula Escuer, Honglin Feng, Astrid Forneck, Toni Gabaldón, Roderic Guigó, Frédérique Hilliou, Silvia Hinojosa-Alvarez, Yi-min Hsiao, Sylvie Hudaverdian, Emmanuelle Jacquin-Joly, Edward B. James, Spencer Johnston, Benjamin Joubard, Gaëlle Le Goff, Gaël Le Trionnaire, Pablo Librado, Shanlin Liu, Eric Lombaert, Hsiao-ling Lu, Martine Maïbèche, Mohamed Makni, Marina Marcet-Houben, David Martínez-Torres, Camille Meslin, Nicolas Montagné, Nancy A. Moran, Daciana Papura, Nicolas Parisot, Yvan Rahbé, Mélanie Ribeiro Lopes, Aida Ripoll-Cladellas, Stéphanie Robin, Céline Roques, Pascale Roux, Julio Rozas, Alejandro Sánchez-Gracia, Jose F. Sánchez-Herrero, Didac Santesmasses, Iris Scatoni, Rémy-Félix Serre, Ming Tang, Wenhua Tian, Paul A. Umina, Manuella van Munster, Carole Vincent-Monégat, Joshua Wemmer, Alex C. C. Wilson, Ying Zhang, Chaoyang Zhao, Jing Zhao, Serena Zhao, Xin Zhou, François Delmotte, Denis Tagu

**Affiliations:** 1 BIOEPAR, INRAE, Oniris, Nantes, France; 2 BIPAA, IGEPP, Agrocampus Ouest, INRAE, Université de Rennes 1, 35650 Le Rheu, France; 3 Department of Botany and Plant Sciences, University of California, Riverside, USA; 4 Bioinformatics and Genomics Unit, Centre for Genomic Regulation (CRG), Barcelona Institute of Science and Technology, Dr. Aiguader, 88, 08003 Barcelona, Spain; 5 Present address: Institute of Evolutionary Biology (CSIC-UPF), Passeig marítim de la Barceloneta 37-49, 08003 Barcelona, Spain; 6 Department of Entomology, Cornell University, Ithaca, NY 14853 USA; 7 Univ Lyon, INSA-Lyon, INRAE, BF2I, UMR0203, F-69621 Villeurbanne, France; 8 Department of Biology, University of Miami, Coral Gables, FL 33146 USA; 9 Facultad de Agronomía, Montevideo, Uruguay; 10 Institut de Biologia Integrativa de Sistemes, Parc Cientific Universitat de Valencia, C/ Catedrático José Beltrán n° 2, 46980 Paterna, València Spain; 11 Université de Tunis El Manar, Faculté des Sciences de Tunis, LR01ES05 Biochimie et Biotechnologie, 2092 Tunis, Tunisia; 12 Department Biol, Univ Rochester, Rochester, NY 14627 USA; 13 Laboratoire Evolution, Génomes, Comportement, Ecologie CNRS, Univ. Paris-Sud, IRD, Université Paris-Saclay, Gif-sur-Yvette, France; 14 LIPM, Université de Toulouse, INRAE, CNRS, Castanet-Tolosan, France; 15 Sorbonne Université, UPEC, Université Paris 7, INRAE, CNRS, IRD, Institute of Ecology and Environmental Sciences, Paris, France; 16 SAVE, INRAE, Bordeaux Sciences Agro, Villenave d’Ornon, France; 17 Department of Molecular Biology and Genetics, Cornell University, Ithaca, NY 14853 USA; 18 Department of Cell and Molecular Biology, College of the Environment and Life Sciences, University of Rhode Island, Kingston, RI USA; 19 Departament de Genètica, Microbiologia i Estadística and Institut de Recerca de la Biodiversitat (IRBio), Universitat de Barcelona, 08028 Barcelona, Spain; 20 Department of Biology, University of Miami, Coral Gables, USA; 21 Current affiliation: Boyce Thompson Institute for Plant Research, Cornell University, Ithaca, USA; 22 Universität für Bodenkultur (BOKU), Vienna, Austria; 23 Universitat Pompeu Fabra, 08003 Barcelona, Spain; 24 Institució Catalana de Recerca i Estudis Avançats (ICREA), Pg. Lluís Companys 23, 08010 Barcelona, Spain; 25 Centre for Genomic Regulation (CRG), The Barcelona Institute of Science and Technology, Barcelona, Spain; 26 Universitat Pompeu Fabra (UPF), Barcelona, Spain; 27 Université Côte d’Azur, INRAE, CNRS, Institut Sophia Agrobiotech, Sophia-Antipolis, France; 28 Institute of Biotechnology and Department of Entomology, College of Bioresources and Agriculture, National Taiwan University, Taipei, Taiwan; 29 Present affiliation: Bone and Joint Research Center, Chang Gung Memorial Hospital, Taoyuan, Taiwan; 30 IGEPP, Agrocampus Ouest, INRAE, Université de Rennes 1, 35650 Le Rheu, France; 31 INRAE, Institute of Ecology and Environmental Sciences, Versailles, France; 32 Department of Entomology, Texas A&M University, College Station, TX 77843 USA; 33 Université Côte d’Azur, INRAE, CNRS, Institut Sophia Agrobiotech, Sophia-Antipolis, France; 34 Laboratoire d’Anthropobiologie Moléculaire et d’Imagerie de Synthèse, CNRS UMR 5288, Université de Toulouse, Université Paul Sabatier, Toulouse, France; 35 China National GeneBank-Shenzhen, BGI-Shenzhen, Shenzhen, 518083 Guangdong Province People’s Republic of China; 36 BGI-Shenzhen, Shenzhen, 518083 Guangdong Province People’s Republic of China; 37 Department of Entomology, College of Plant Protection, China Agricultural University, Beijing, 100193 People’s Republic of China; 38 Université Côte d’Azur, INRAE, CNRS, ISA, Sophia Antipolis, France; 39 Department of Post-Modern Agriculture, MingDao University, Changhua, Taiwan; 40 Institut de Biologia Integrativa de Sistemes, Parc Cientific Universitat de Valencia, C/ Catedrático José Beltrán n° 2, 46980 Paterna, València Spain; 41 Sorbonne Université, Institute of Ecology and Environmental Sciences, Paris, France; 42 Department of Integrative Biology, University of Texas at Austin, Austin, USA; 43 Univ Lyon, INRAE, INSA-Lyon, CNRS, UCBL, UMR5240 MAP, F-69622 Villeurbanne, France; 44 BIPAA IGEPP, Agrocampus Ouest, INRAE, Université de Rennes 1, 35650 Le Rheu, France; 45 Plateforme Génomique GeT-PlaGe, Centre INRAE de Toulouse Midi-Pyrénées, 24 Chemin de Borde Rouge, Auzeville, CS 52627, 31326 Castanet-Tolosan Cedex, France; 46 Division of Genetics, Department of Medicine, Brigham and Women’s Hospital, Harvard Medical School, Boston, MA 02115 USA; 47 Facultad de Agronoía, Montevideo, Uruguay; 48 School of BioSciences, The University of Melbourne, Parkville, VIC Australia; 49 BGPI, Université Montpellier, CIRAD, INRAE, Montpellier SupAgro, Montpellier, France

**Correction to: BMC Biol 18, 90 (2020)**

**https://doi.org/10.1186/s12915-020-00820-5**

Following publication of the original article [1], the authors identified an error in Fig. 4. The error was made in the x-axis labels of the three panels, which were noted as “pairwise dN/dS”. The figure represents distributions of dS, so the labels were corrected as “pairwise dS”. The correct Fig. 4 is given below.

**Fig. 4:**
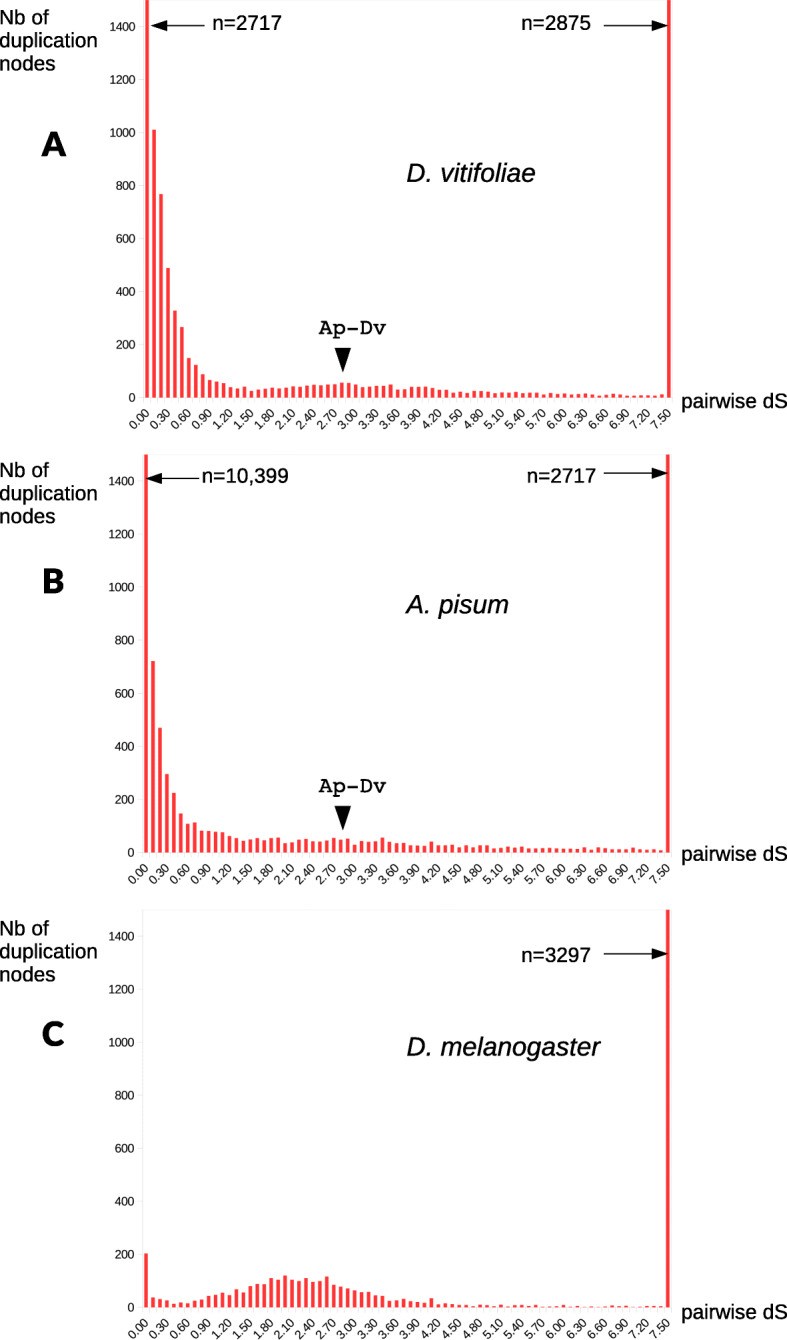
Distribution of synonymous distances among paralogs for grape vine phylloxera (panel **a**, *D. vitifoliae),* pea aphid (panel **b**, *A. pisum*), and fruit fly (panel **c**, *D. melanogaster*). Paralogs were identified as RBH pairs, with an iterative approach allowing to cover both recent duplications (terminal nodes in gene families) and more ancient duplications (internal nodes). For readability, the *y*-axis (number of dS classes) is truncated to 1500 (numbers above that threshold are indicated on the figures). For both *A. pisum* and *D. vitifoliae*, an arrowhead indicates the median dS between orthologs (RBH genes between the two species), dS = 2.83: this metric, a proxy of the age of separation between the two species allows to distinguish duplications that are more recent (left of the arrow, lower dS values) *or more ancient (right of the arrowhead, higher dS) than the speciation event
