# Supplementary material for: The genome sequence of the grape phylloxera provides insights into the evolution, adaptation, and invasion routes of an iconic pest
Source: BMC Biol. 2020 Jul 23;18:90. doi: 10.1186/s12915-020-00820-5 (PMC7376646; doi:10.1186/s12915-020-00820-5)
Supplement: Supplementary file 1 — Additional file 1: Figures. S1-S22, Table S1-S20, Methods and Results. Figure S1. Mitochondrial genome view of grape phylloxera. Figure S2. Proportion of transposable elements (TE) in the genome. Figure S3. GO terms of phylloxera-specific genes. Figure S4. Enriched GO terms in the phylloxera genome with and without TEs. Figure S5. Gene gain/loss at different nodes or branches. Figure S6. Species phylogenetic tree based on insect genomes and the transcriptomes of Planoccoccus citri and Adelges tsugae. Figure S7. Diagram of the gap-filling and annotation process. Figure S8. Urea cycle in D. vitifoliae and A. pisum. Figure S9. IMD immune pathway in D. vitifoliae.Figure S10. Phylogenetic tree of RR-1 cuticular proteins.Figure S11. Phylogenetic tree of RR-2 cuticular proteins.Figure S12. Comparison of miRNAs in D. vitifoliae and other insect genomes. Figure S13. Phylogenetic tree of aquaporin protein sequences. Figure S14. Comparison of the phylloxera PER protein with other insects. Figure S15. Amino acid alignment of PTTH amino acid sequences. Figure S16. Phylogeny of hemipteran ORs. Figure S17. Phylogeny of hemipteran GRs. Figure S18. Phylogenetic analysis of OBPs. Figure S19. Phylogenetic analysis of CSPs. Figure S20. Phylogenetic analysis of NPC2s. Figure S21. Distribution of cluster sizes of putative effectors. Figure S22. Physical distribution of the three largest clusters of effectors. Table S1. Genes of bacterial and fungal origin. Table S2. Statistics on TEs. Table S3. GO enrichment of genes duplicated at different ancestral nodes. Table S4. Metabolic gaps in the D. vitifoliae reaction network. Table S5. Functional annotation of metabolic genes. Table S6. Genes of the TOLL pathway. Table S7. Genes of the IMD pathway. Table S8. Statistics on cuticular proteins. Table S9. Developmental genes in D. vitifoliae and A. pisum. Table S10. miRNAs. Table S11. Clock-related genes. Table S12. List of ORs and GRs. Table S13. Number of OBPs, CSPs and NPC2s. Table S14. List of [file 12915_2020_820_MOESM1_ESM.pdf]

### Supplementary Information for

# The genome sequence of the grape phylloxera provides insights into the evolution, adaptation and invasion routes of an iconic pest

**Claude Rispe*$, Fabrice Legeai*$,** Paul Nabity, Rosa Fernández, Arinder K. Arora, Patrice Baa-Puyoulet, Celeste R. Banfill, Leticia Bao, Miquel Barberà, Maryem Bouallègue, Anthony Bretaudeau, Jennifer A. Brisson, Federica Calevro, Pierre Capy, Olivier Catrice, Thomas Chertemps, Carole Couture, Laurent Delière, Angela E. Douglas, Keith Dufault-Thompson, Paula Escuer, Honglin Feng, Astrid Forneck, Toni Gabaldón, Roderic Guigó, Frédérique Hilliou, Silvia Hinojosa-Alvarez, Yi-min Hsiao, Sylvie Hudaverdian, Emmanuelle Jacquin-Joly, Edward B. James, Spencer Johnston, Benjamin Joubard, Gaëlle Le Goff, Gaël Le Trionnaire, Pablo Librado, Shanlin Liu, Eric Lombaert, Hsiao-ling Lu , Martine Maïbèche, Mohamed Makni, Marina Marcet-Houben, David Martínez-Torres, Camille Meslin, Nicolas Montagné, Nancy A. Moran, Daciana Papura, Nicolas Parisot, Yvan Rahbé, Mélanie Ribeiro Lopes, Aida Ripoll-Cladellas, Stéphanie Robin, Céline Roques, Pascale Roux, Julio Rozas, Alejandro Sánchez-Gracia, J11ose F. Sánchez-Herrero, Didac Santesmasses, Iris Scatoni, Rémy-Félix Serre, Ming Tang, Wenhua Tian, Paul A. Umina, Manuella van Munster, Carole Vincent-Monégat, Joshua Wemmer, Alex C. C. Wilson, Ying Zhang, Chaoyang Zhao, Jing Zhao, Serena Zhao, Xin Zhou, **François Delmotte*£, Denis Tagu*£**

*: corresponding authors, $: co-first authors, £: co-last authors

## Additional files

**Additional File 1: Figures S1-S22, Table S1-S20, Methods and Results.** **FigS1.** Mitochondrial genome view of grape phylloxera. **FigS2.** Proportion of transposable elements (TE) in the genome. **FigS3.** GO terms of phylloxera-specific genes. **FigS4.** Enriched GO terms in the phylloxera genome with and without TEs. **FigS5.** Gene gain/loss at different nodes or branches. **FigS6.** Species phylogenetic tree based on insect genomes and the transcriptomes of *Planoccoccus citri* and *Adelges tsugae*. **FigS7.** Diagram of the gap-filling and

annotation process. **FigS8.** Urea cycle in *D. vitifoliae* and *A. pisum*. **FigS9.** IMD immune pathway in *D. vitifoliae.* **FigS10.** Phylogenetic tree of RR-1 cuticular proteins. **FigS11.** Phylogenetic tree of RR-2 cuticular proteins. **FigS12.** Comparison of miRNAs in *D. vitifoliae* and other insect genomes. **FigS13.** Phylogenetic tree of aquaporin protein sequences. **FigS14.** Comparison of the phylloxera PER protein with other insects. **FigS15.** Amino acid alignment of PTTH amino acid sequences. **FigS16.** Phylogeny of hemipteran ORs. **FigS17.** Phylogeny of hemipteran GRs. **FigS18.** Phylogenetic analysis of OBPs. **FigS19.** Phylogenetic analysis of CSPs. **FigS20.** Phylogenetic analysis of NPC2s. **FigS21.** Distribution of cluster sizes of putative effectors. **FigS22.** Physical distribution of the three largest clusters of effectors. **TabS1.** Genes of bacterial and fungal origin.**TabS2.** Statistics on TEs. **TabS3.** GO enrichment of genes duplicated at different ancestral nodes. **TabS4.** Metabolic gaps in the D. vitifoliae reaction network. **TabS5.** Functional annotation of metabolic genes. **TabS6.** Genes of the TOLL pathway. **TabS7.** Genes of the IMD pathway. **TabS8.** Statistics on cuticular proteins. **TabS9.** Developmental genes in *D. vitifoliae* and *A. pisum*. **TabS10.** miRNAs. **TabS11.** Clock-related genes. **TabS12.** List of ORs and GRs. **TabS13.** Number of OBPs, CSPs and NPC2s. **TabS14.** List of Cytochromes P450. **TabS15.** List of genes involved  in detoxification. **TabS16.** Effector genes with predicted domains and their corresponding functions. **TabS17.** Statistics on sequence reads and SRA accessions used for the reference genome. **TabS18.** List of species used to study gene expansions. **TabS19.** Sampling sites and SRA used for population genomics analyses. **TabS20.** Prior distribution of parameters used for ABC modelling of invasion routes.

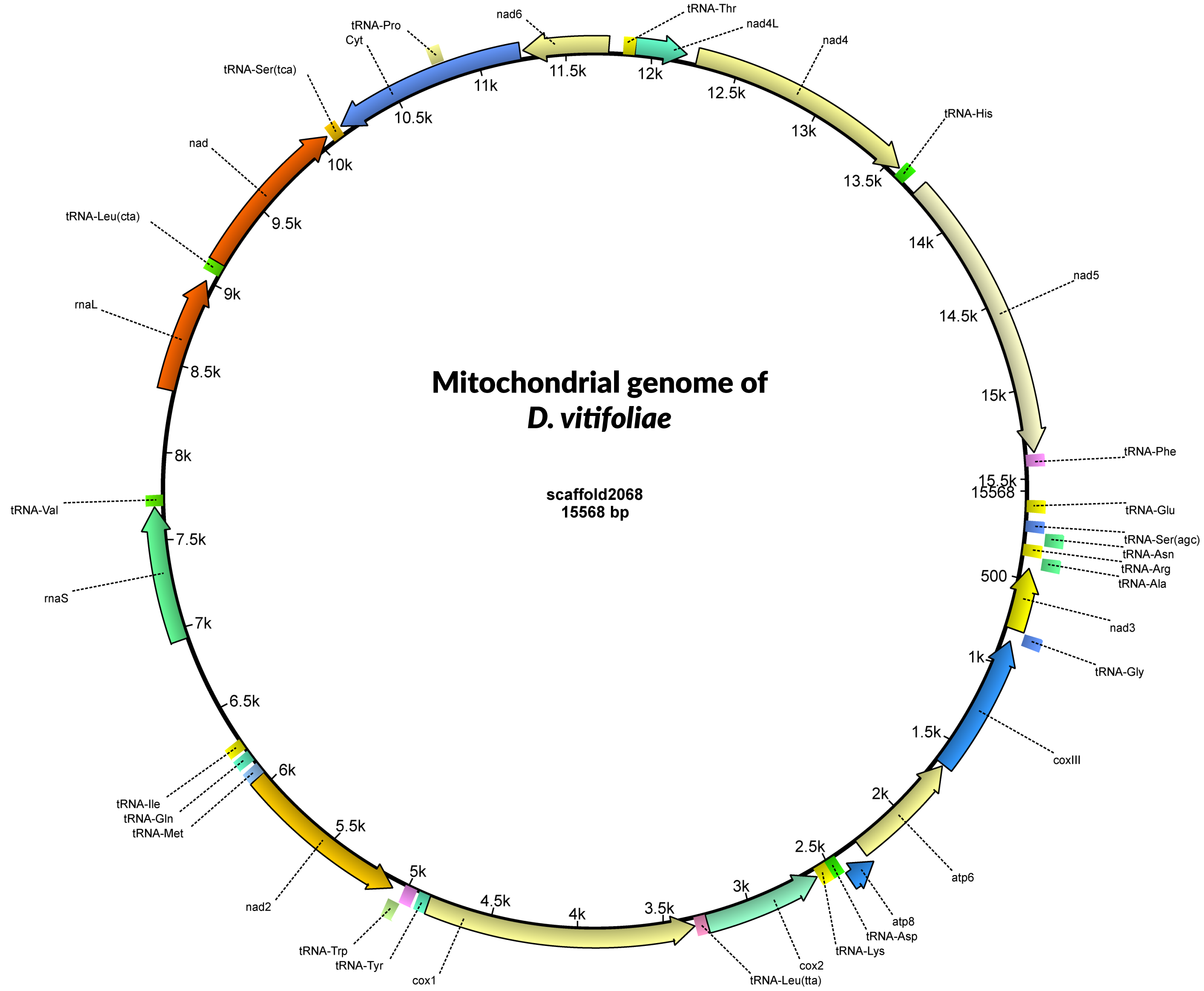


**Figure S1.** Mitochondrial genome view after editing of the Nd5 gene. The contig is shown in its circular form. The phylloxera mitochondrial scaffold is slightly smaller (15568 bp) than the mitochondrial genomes from *A. pisum* (16971 bp) and *D. melanogaster* (19517 bp) but has similar GC content to both other species (15.5% vs 15.2% and 17.8% respectively). The protein sequences shared an average of 78% identity with their homologs in *A. pisum* (ranging between 53% and 94%); average identity with *D. melanogaster* homologs was 49% (ranging between 31% and 72%).

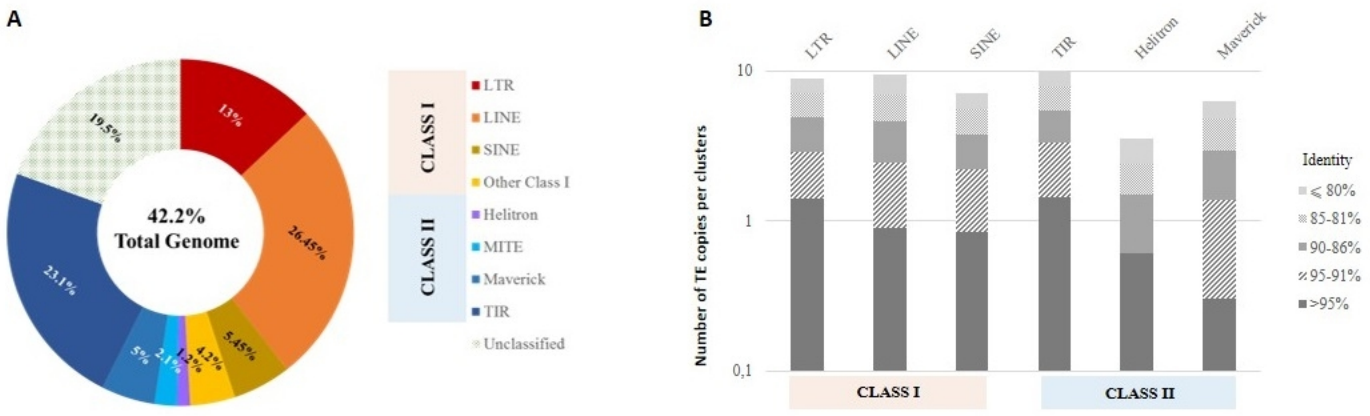


**Figure S2.** Proportion of transposable elements (TE) in the phylloxera draft genome. A. ercentage of the phylloxera genome occupied by the main Orders of TE (according to the classification of Wicker et al. [18]). Long interspersed elements (LINE) and Terminal Inverted Repeats (TIR) are the most frequent elements. Unclassified elements (according to REPET) have no structural features or similarity with other known TEs. B. Identity levels between TE of the different clusters defined by REPET. Identities are calculated from pairwise comparison between all TE of a cluster. The clusters are grouped according to the main Orders of TE. The y-axis is a log scale. Clusters with a high level of identity probably correspond to the recently expanded TE family. On the contrary, clusters with low identity values are probably old families. Repeat Orders previously named ‘‘Other Class I”, “MITE’’ and “Unclassified” are not shown included in this analysis.

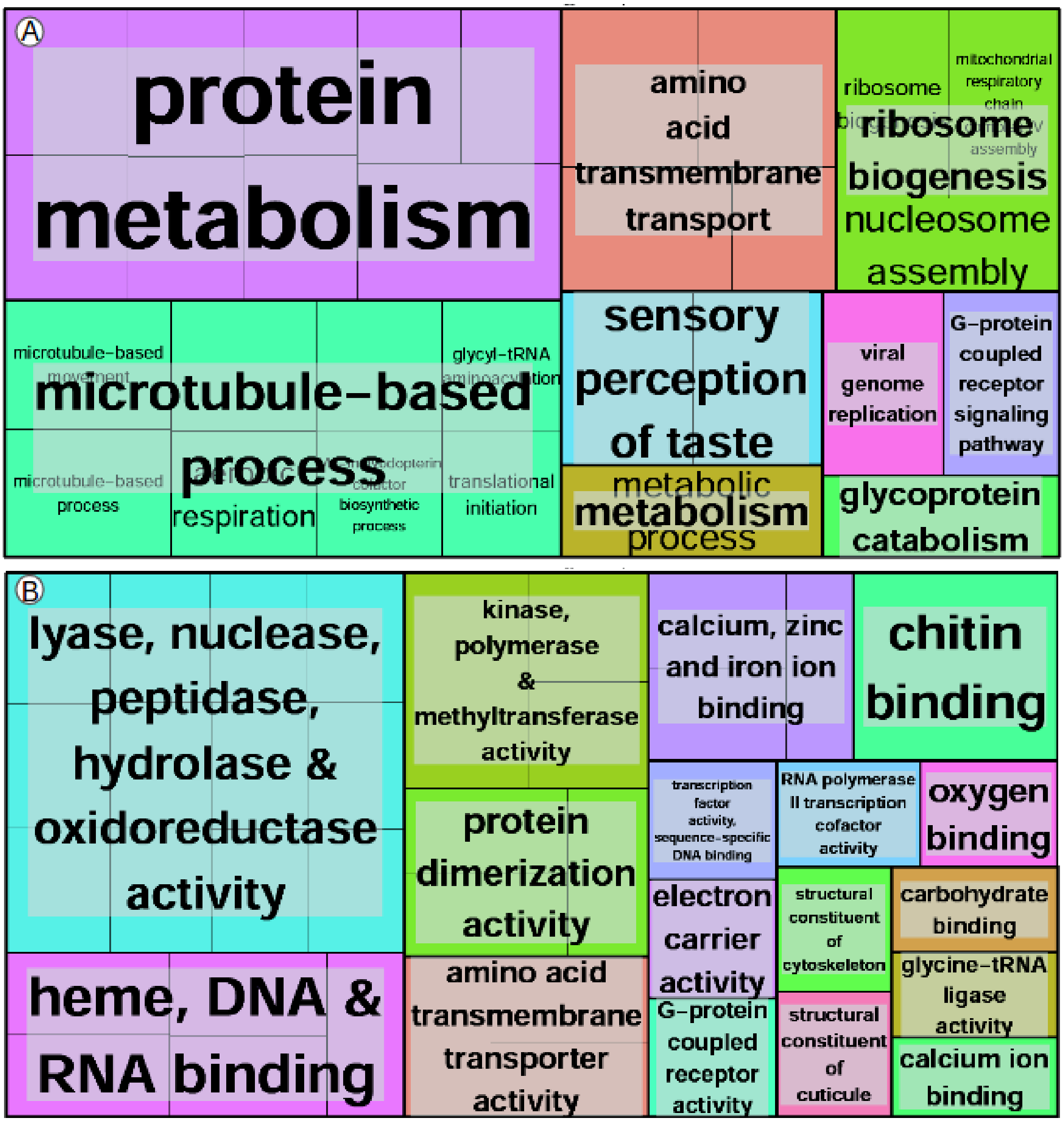


**Figure S3**. REVIGO tree maps summarizing GO terms in the 363 lineage-specific genes in phylloxera genome at the biological process level (A) and molecular function level (B). The size of each square is related to the uniqueness of each category.

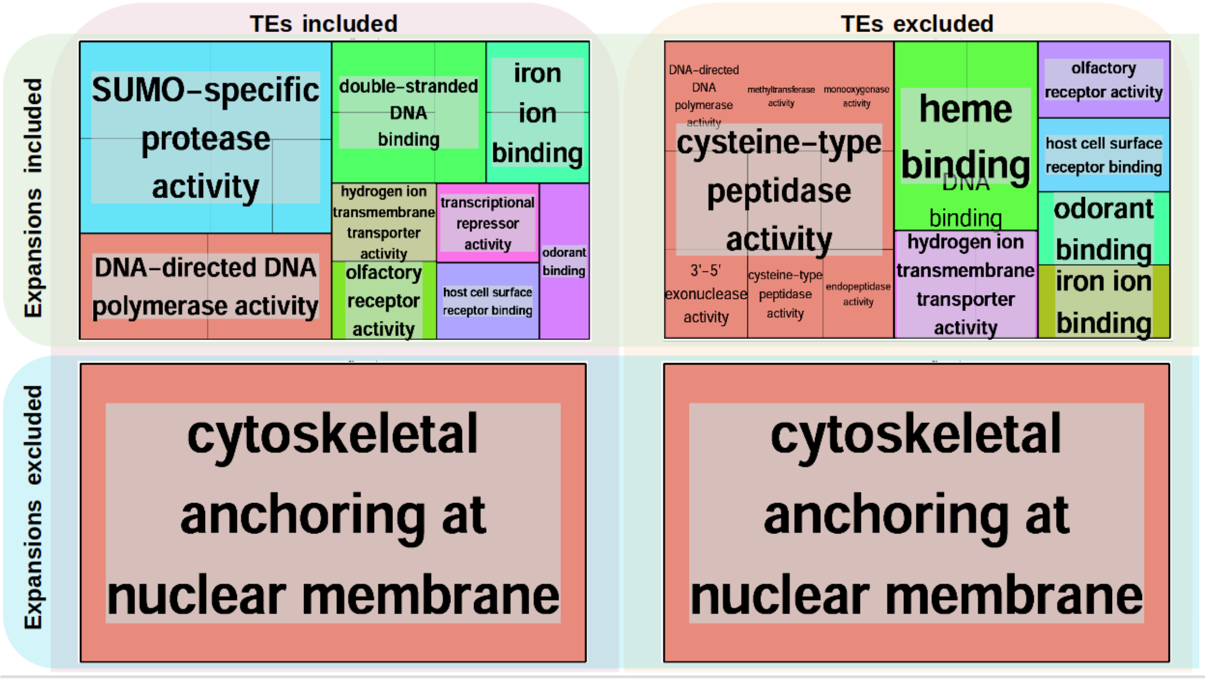


**Figure S4.** REVIGO tree maps summarizing enriched GO terms in the phylloxera genome (molecular function). The effect of inclusion and exclusion of expansions and TEs in the enrichment analysis is shown by the different composition of terms in the treemaps. The size of each square is related to the significance of p-values in the enrichment analyses.

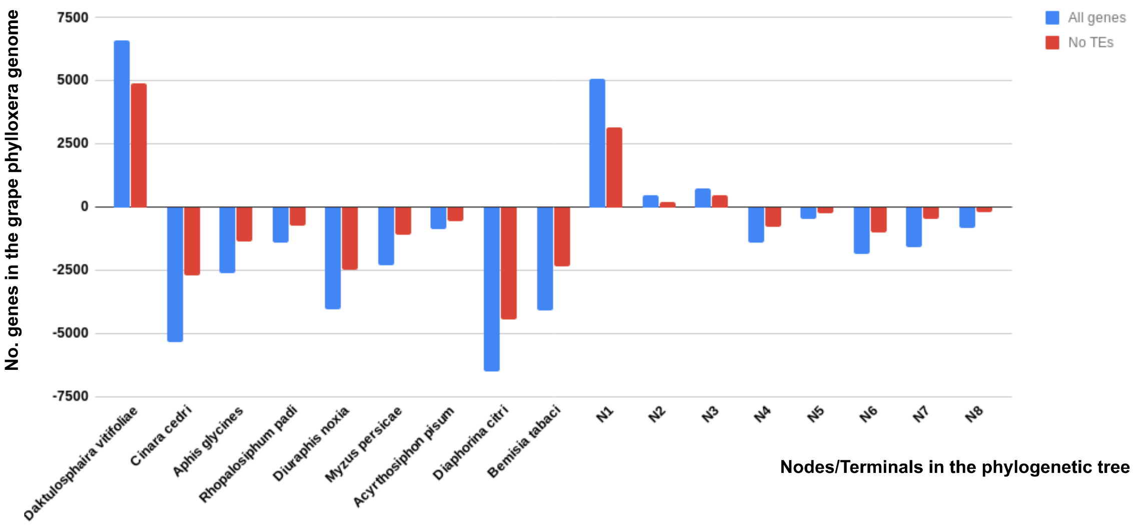


**Figure S5.** Histogram of gene gain/loss reference to the gene content of *D. vitifoliae*. Values represent the number of genes of the genome of the grape phylloxera that have been gained or lost in each taxon or nodes. Note that genes can only be gained at nodes previous to or during the split of phylloxera (*i.e.*, phylloxera-specific genes), and only be lost at nodes before or after any splits not involving phylloxera (*i.e.*, the remaining clades in the tree). Node numbers refer to a phylogenetic study based on the complete genomes of 14 different species of insects (Figure 3).

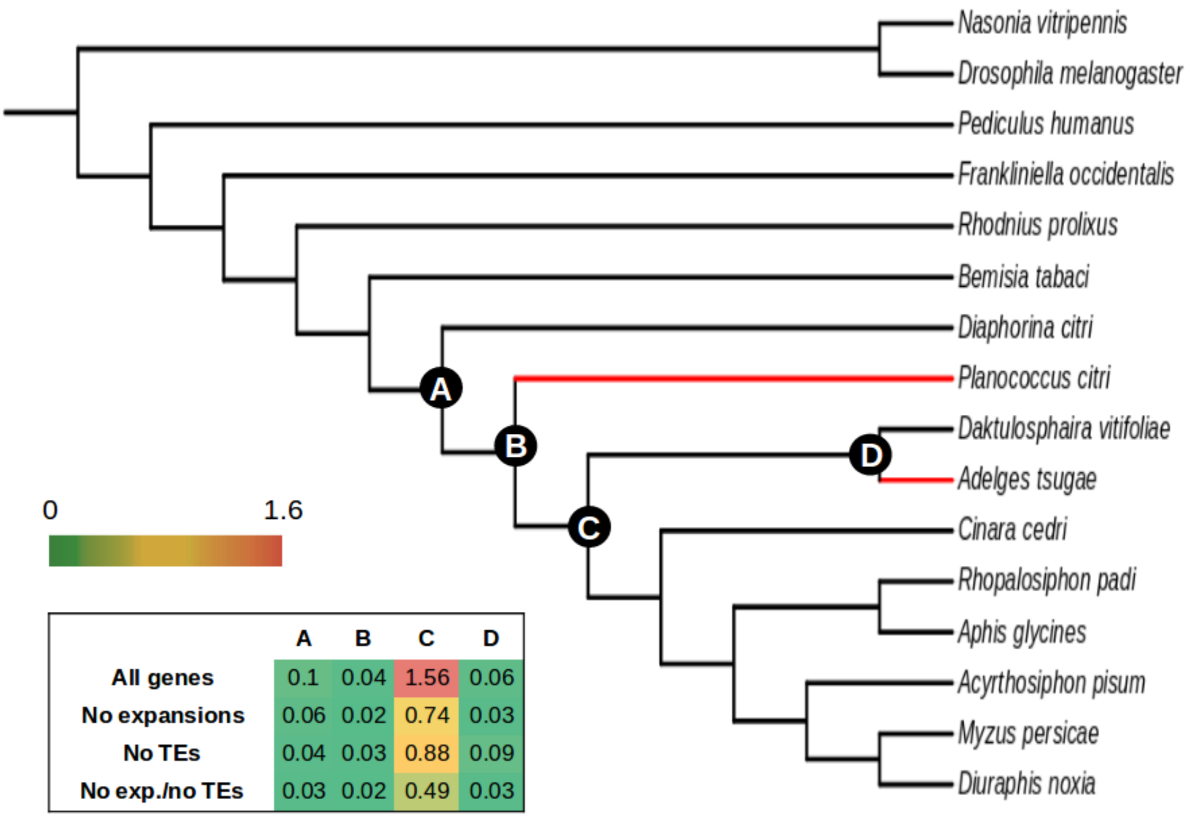


| | A | B | C | D |
|---|---|---|---|---|
| All genes | 0.1 | 0.04 | 1.56 | 0.06 |
| No expansions | 0.06 | 0.02 | 0.74 | 0.03 |
| No TEs | 0.04 | 0.03 | 0.88 | 0.09 |
| No exp./no TEs | 0.03 | 0.02 | 0.49 | 0.03 |

**Figure S6.** Species tree based on one-to-one orthologs inferred in the data set after inclusion of *Planococcus citri* (Coccoidea) and *Adelges tsugae* (Adelgidae) (branches highlighted in red). All nodes were highly supported (>0.95 SH-like support). Duplication ratios considering all genes and excluding proteins encoded by transposable elements (TE), as well as including and excluding phylloxera-specific expansions, are indicated for the three most basal nodes of Sternorrhyncha and Hemiptera (A, B and C), and the node of Phylloxeridae and Adelgidae (D).

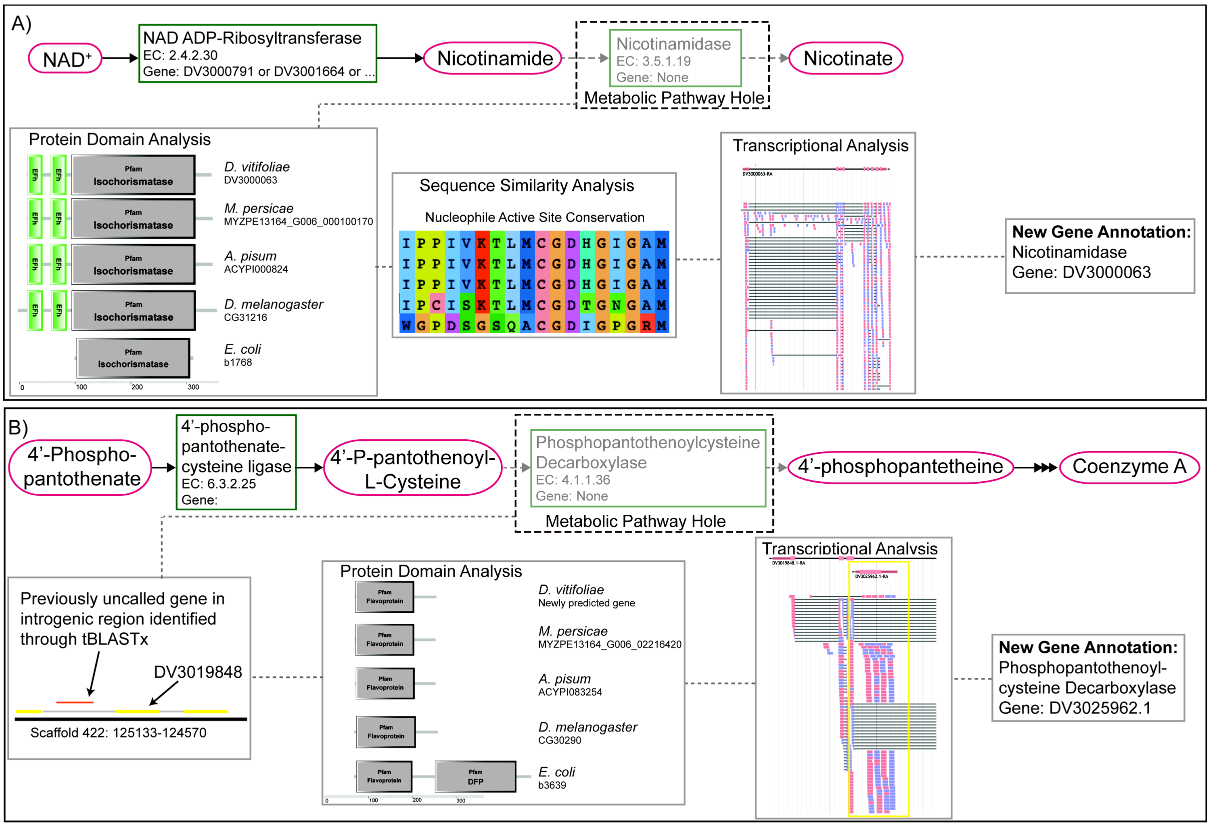


**Figure S7.** A diagrammatic representation of the metabolic gap-filling and annotation process. Pathway gaps are shown as faded reaction boxes in the pathway diagram. Visual representations of the evidence used during the gene annotation process for DV3000063 (A) and DV3025962.1 (B) are shown in boxes below the pathway diagrams.

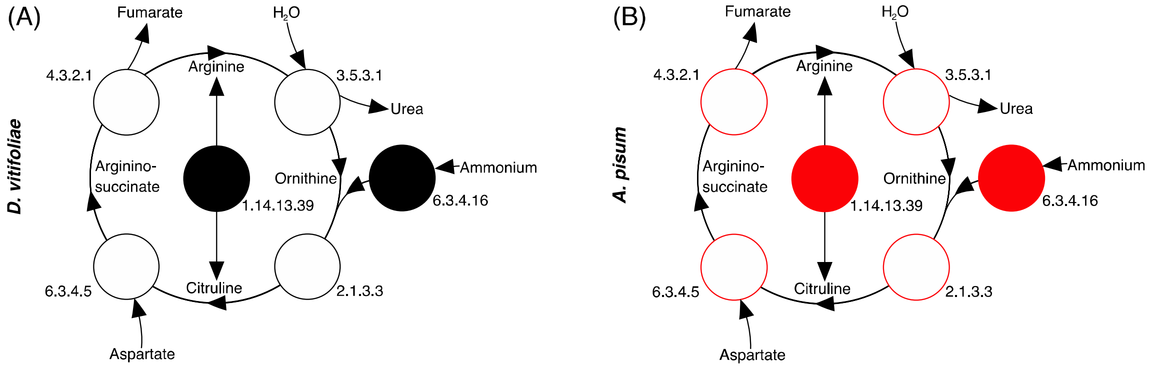


**Figure S8.** A comparison of the urea cycle between *D. vitifoliae* (left cycle, with enzymes shown by black circles and the aphid *A. pisum* (right cycle, with enzymes shown by red circles). The presence of an enzyme in the cycle is shown by a filled circle, and absence by an empty circle. Enzymes are identified by EC number. All annotations are from Dakvicyc and gap-filling analysis, except for 1.14.13.39, which was annotated via blastp and HMM searches based on KEGG.

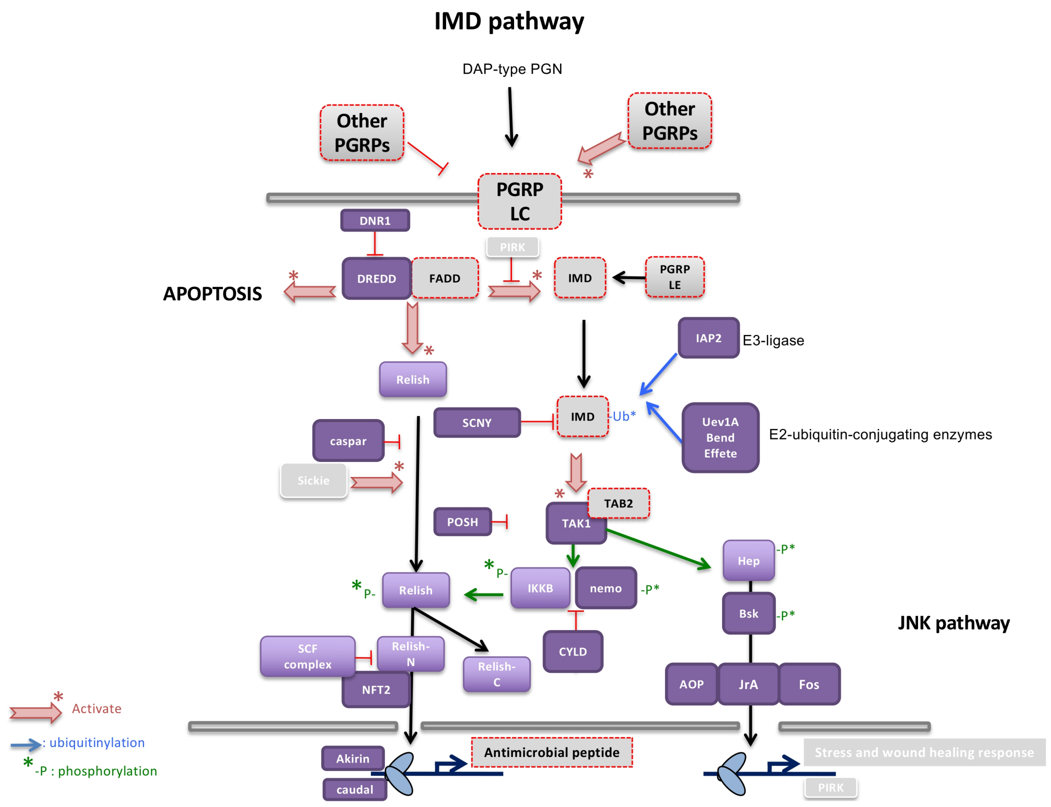


**Figure S9.** Schematic representation of the putative IMD immune pathway in *D. vitifoliae*. All the proteins present in the reference pathway (*D. melanogaster*) are indicated. Phylloxera proteins with high similarity scores are shown in purple boxes. Proteins with lower similarity scores are shown in clear purple boxes. Missing proteins are shown in gray boxes highlighted in red. Light gray boxes contain proteins that were not searched for. Red arrows show the potential to activate a target protein. Ubiquitinylation targets are represented by blue arrows and phosphorylation by green arrows, respectively.

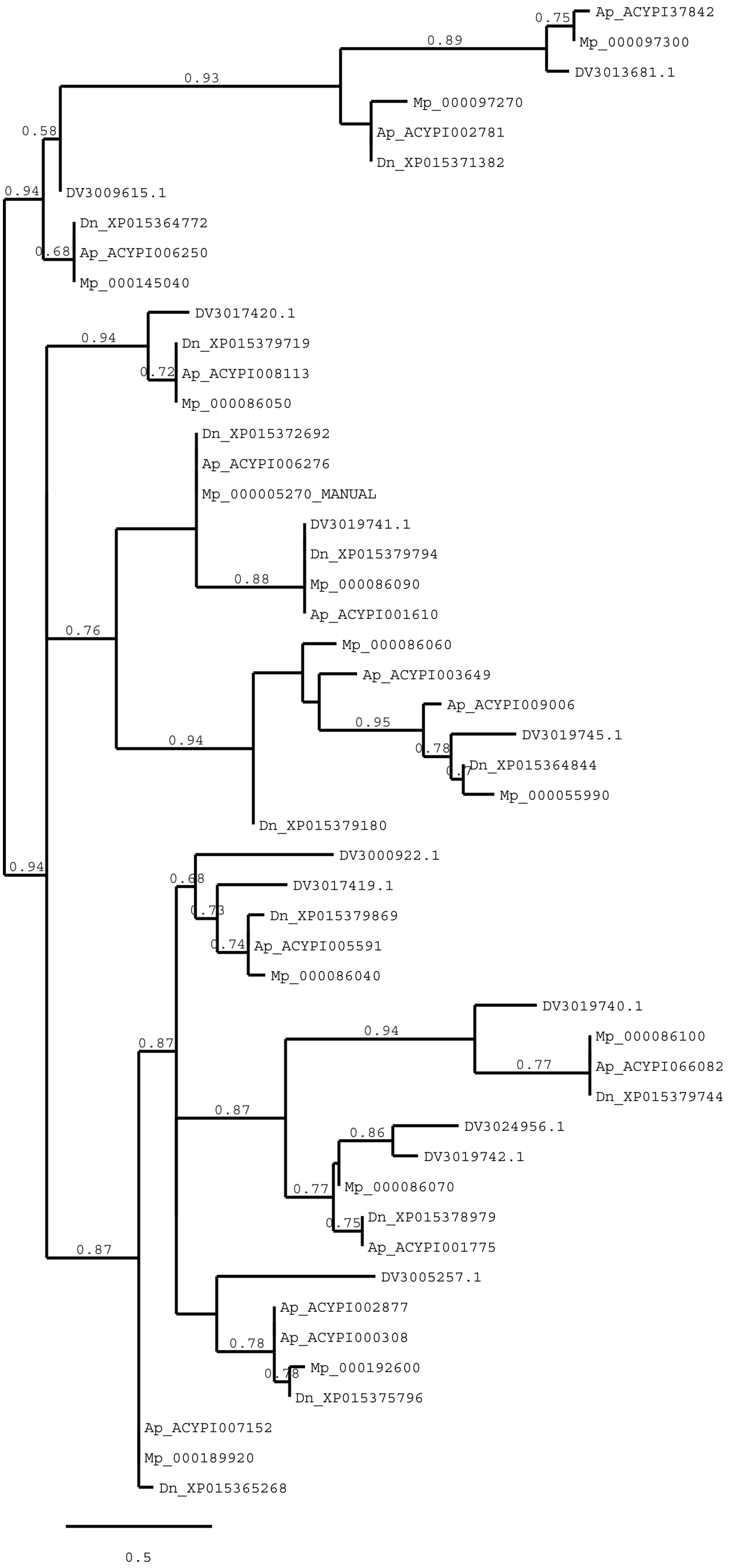


**Figure S10.** Phylogenetic relationships of full RR-1 proteins of *D. vitifoliae*, *D. noxia*, *A. pisum* and *M. persicae*. Phylogenetic reconstruction was performed using an updated list of RR-1 proteins of the different aphid species and *D. vitifoliae* as described in Material & Methods section. Numbers at nodes indicate the aLRT value that supports each node. The scale represents probabilities of change from one amino acid to another in terms of a unit, which is an expected 1 % change between two amino acid sequences.

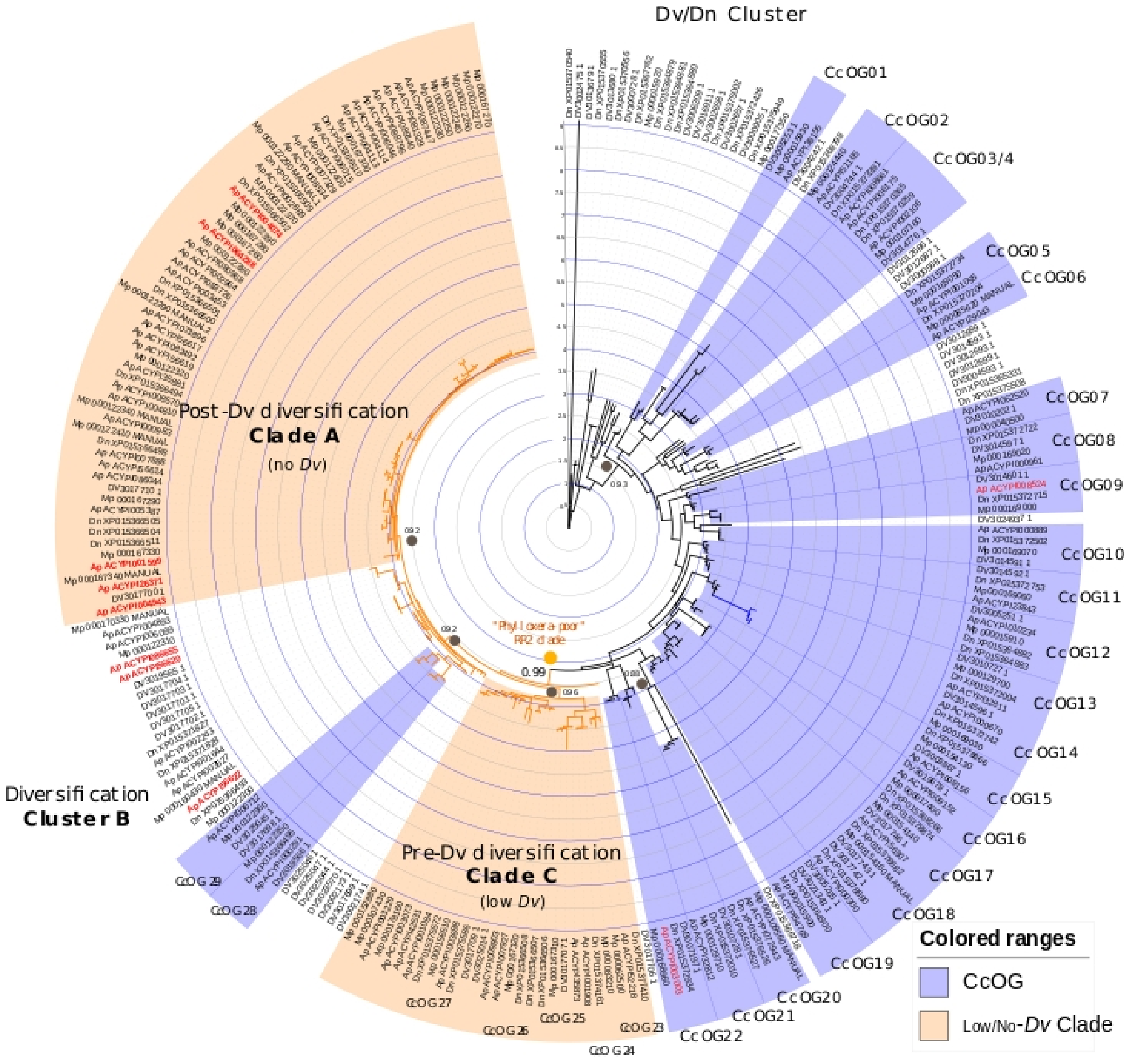


**Figure S11.** Phylogenetic relationships of the core RR-2 proteins of *D. vitifoliae* and three aphid species, *D. noxia* , *A. pisum* and *M. persicae.* Phylogenetic reconstruction was performed using the extended domain of 69 amino acids specific of RR2-proteins. Selected numbers at nodes indicate the percentage of 1000 bootstrap replicates. Groups of one-to-one orthologs among the four species are shaded in blue and numbered (CcOG), as well as clades showing expansions in aphids only (Clade A) or in both aphids and phylloxera.

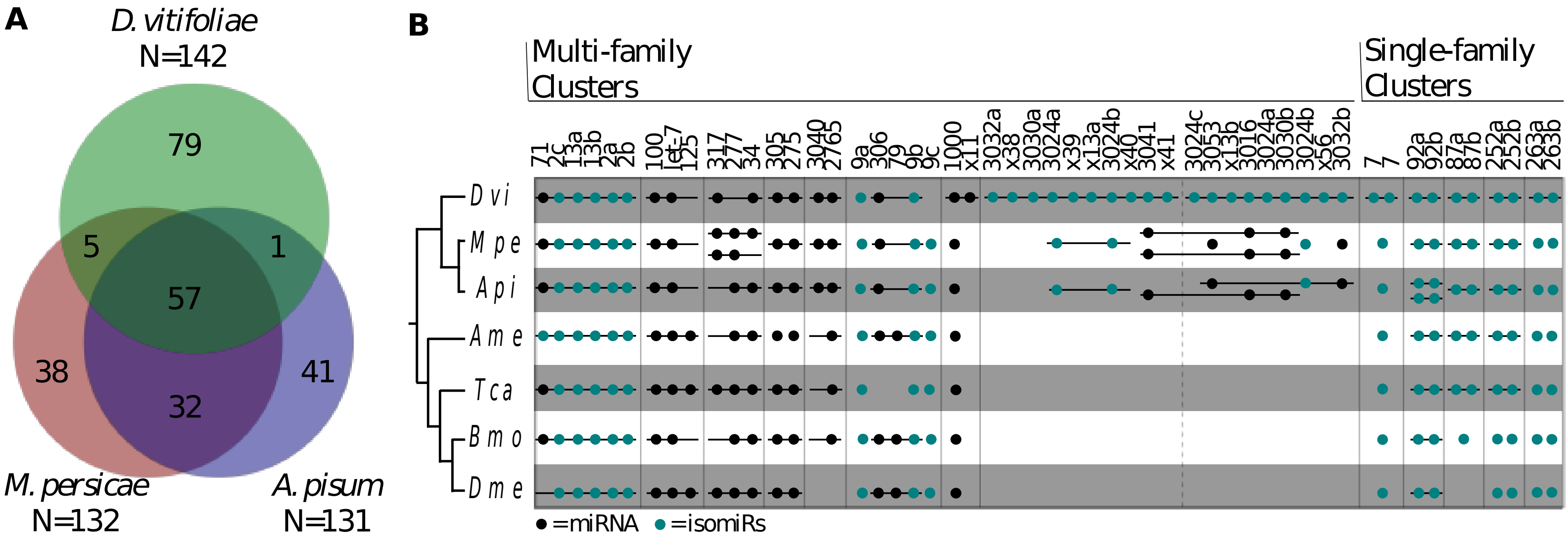


**Supplementary Figure S12.** Comparison of miRNAs in *D. vitifoliae* and other insect genomes. (A) Overlap of mature miRNAs between the genomes of *D. vitifoliae* (green), *A. pisum* (blue) and *M. persicae* (red). (B) Genomic clusters of miRNAs. Similar to other insect taxa that include *M. periscae (Mpe), A. pisum (Api), Aphis mellifera* (*Ame*), *Tribolium castaneum* (*Tca*), *Bombyx mori* (*Bmo*), *Drosophila melanogaster* (*Dme*), 31% (i.e. 47/150 miRNA precursors) of *D. vitifoliae* miRNAs are arranged in 14 genomic clusters. The majority of those miRNA clusters are deeply conserved across different insect taxa. Two large clusters: 3032a/x38/3030a/3024a/x39/x13a/3024b/x40/3041/x41 and 3024c/3053/x13b/3016/3024a/3030b/3024b/x56/3032b share some orthology with aphids. Multi-family clusters are composed of miRNAs from different miRNA families, while single-family clusters are composed of duplicated members of the same miRNA. Each circle marks an individual miRNA; teal circles highlight isomiRs, anciently duplicated miRNAs that share identical seed regions but diverge in sequence of non-seed regions. miRNA names are abbreviated and full names are available by appending “Dvit-miR-” to each of the abbreviated names.

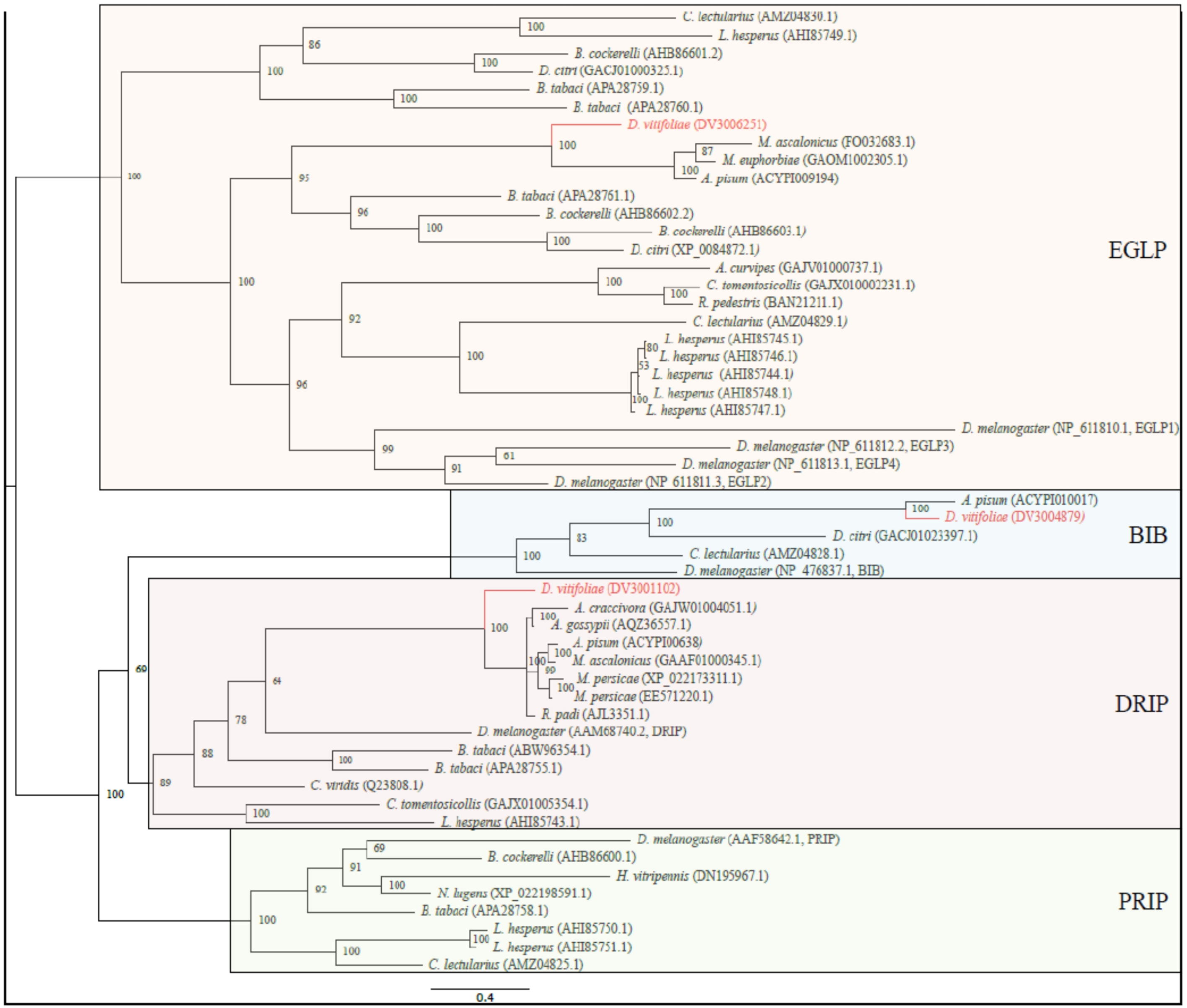


| | NPA motif | | ar/R filter |
|---|---|---|---|
| | loopB | loopE | |
| D. vitifoliae (DV3006251) | NPC | NPV | $F^{73}$ $S^{201}$ $N^{210}$ $R^{216}$ |
| A. pisum (ACYPI009194) | NPC | NPA | $F^{120}$ $S^{247}$ $N^{256}$ $R^{262}$ |
| A. pisum (ACYPI010017) | NPA | NPA | $S^{94}$ $Y^{212}$ $P^{221}$ $C^{227}$ |
| D. vitifoliae (DV3004879) | NPA | NPA | $S^{180}$ $F^{318}$ $P^{327}$ $C^{333}$ |
| D. vitifoliae (DV3001102) | NPA | NPA | $F^{64}$ $H^{183}$ $A^{192}$ $R^{198}$ |
| A. pisum (ACYPI00638) | NPA | NPA | $F^{85}$ $H^{209}$ $A^{218}$ $R^{224}$ |

**Figure S13 (above).** Bayesian phylogenetic tree of aquaporin protein sequences identified in *D. vitifoliae*. Percent posterior probabilities are given on the branches. All sequences were downloaded from NCBI except for *A. pisum*, which was downloaded from AphidBase, as its BIB sequence is not available on NCBI. Abbreviations: DRIP, *Drosophila* integral protein family; PRIP, *Pyrocoelia rufa* integral protein family; EGLP, *Entomoglyceroporin*; BIB, *Drosophila* big brain; *C. lectularius, Cimex lectularius; L. hesperus, Lygus hesperus; B. cockerelli, Bactericera cockerelli*; *D. citri, Diaphorina citri; B. tabaci, Bemisia tabaci; D. vitifoliae, Daktulosphaira vitifoliae; M. ascalonicus, Myzus ascalonicus; M. euphorbiae, Macrosiphum euphorbiae; A. pisum, Acyrthosiphon pisum; A. curvipes, Anoplocnemis curvipes; C. tomentosicollis, Clavigralla tomentosicollis; R. pedestris, Riptortus pedestris; D. melanogaster, Drosophila melanogaster; A. craccivora, Aphis craccivora; R. padi, Rhopalosiphum padi*); *C. viridis, Cicadella viridis*; *H. vitripennis, Homalodisca vitripennis; N. lugens, Nilaparvata lugens*. The amino acids comprising NPA boxes and ar/R selectivity filter of *D. vitifoliae* and *A. pisum* AQPs are displayed in red and black, respectively.

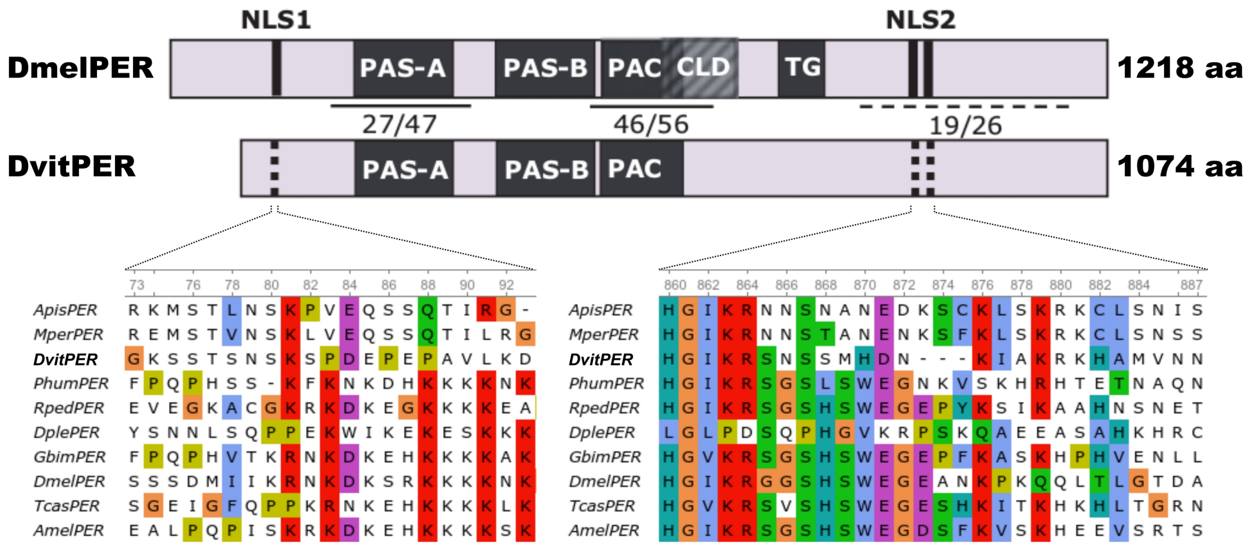


**Figure S14.** Comparison of the phylloxera PER protein with other insects. Top: schematic representation of the whole protein in Drosophila and phylloxera. Bottom: detail of the alignment of PER proteins from different insect species including two aphid species. Note that in the two regions shown (otherwise highly conserved), aphids and phylloxera are both rather divergent from other insects. Accession numbers of sequences used in the alignment are as follows: Apis (*A. pisum*, XP_029344821.1); Mper (*M. persicae*, XP_022163848.1); Phum (*P. humanus*, XP_002426301.1); Rped (R. pedestris, BAG07407.1); Dple (*D plexippus*, AAO48719.1); Gbim (*G. bimaculatus*, BAG48878.1); Dmel (*D. melanogaster*, XP_003399949.1); Tcas (*T. castaneum*, EFA04566.2); Amel (*A. mellifera*, ARB43935.1).

## PTTH

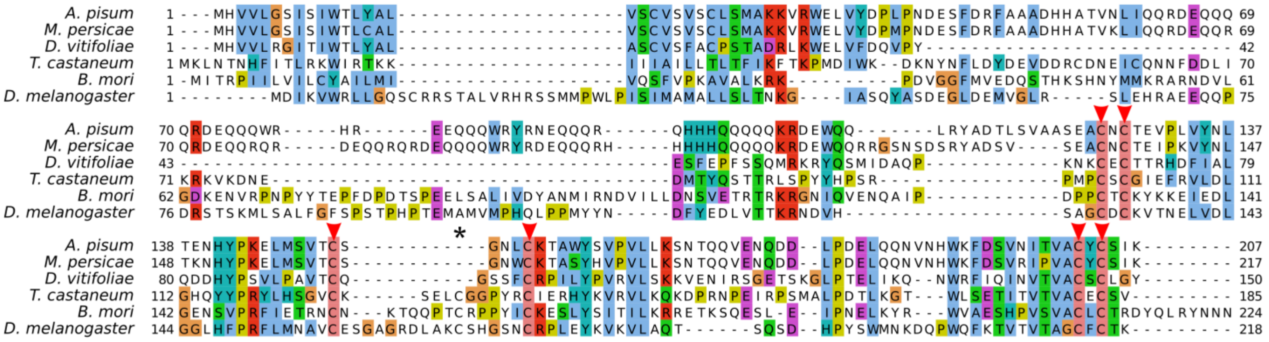


**Figure S15.** Amino acid alignment of PTTH amino acid sequences of different insect species, including aphids and the putative PTTH of *D. vitifoliae*. Conserved cysteine residues are indicated with red arrowheads. The cysteine conserved in all insects except Aphidomorpha is indicated by an asterisk.

## Phylogenetic tree of ORs

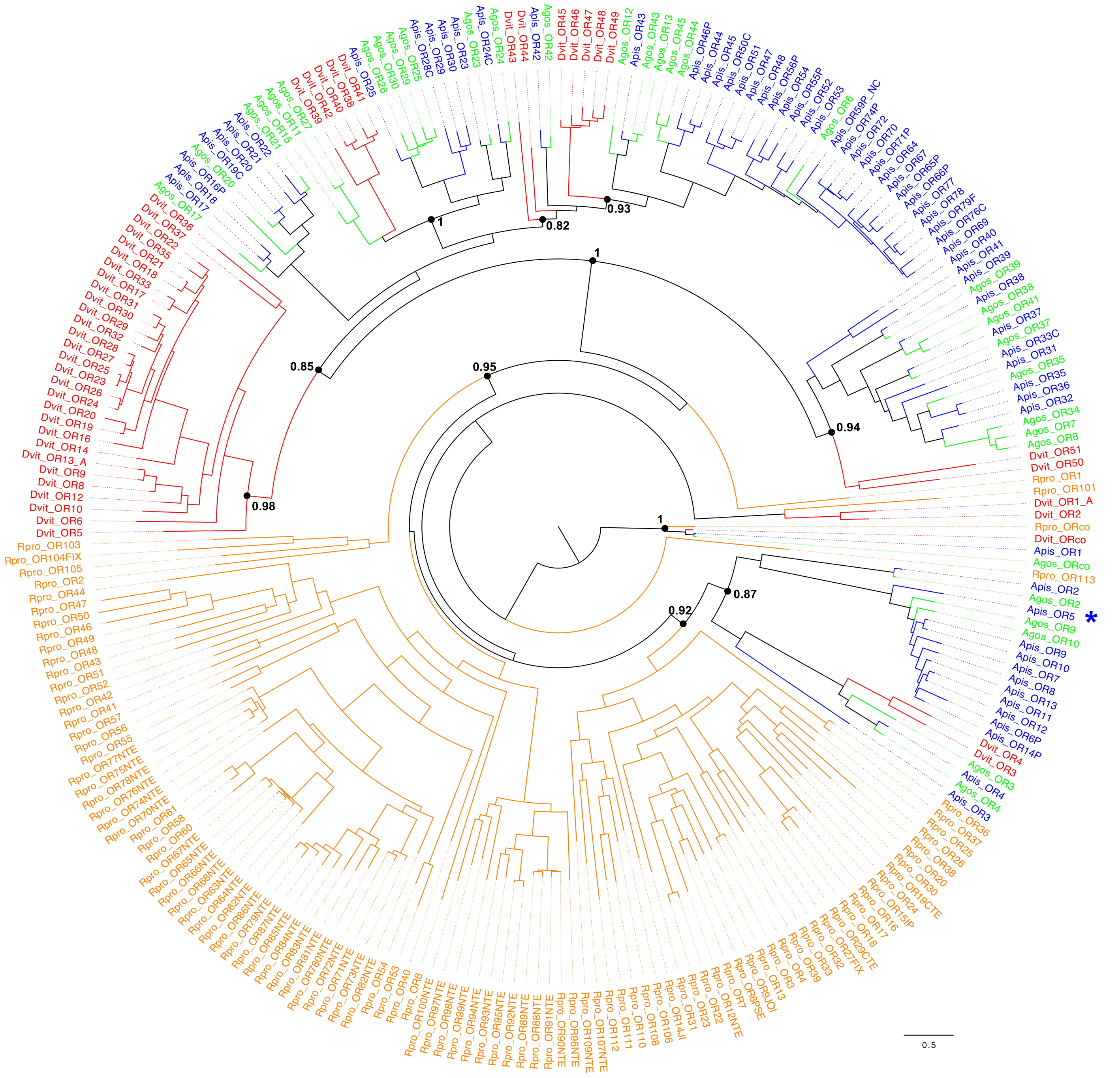


**Figure S16**. Maximum likelihood phylogeny of hemipteran ORs. The amino acid dataset included OR sequences from *D. vitifoliae* (Dvit), *A. pisum* (Apis), *A. gossypii* (Agos) and *R. prolixus* (Rpro). The tree was rooted using the OR co-receptor (ORco) clade as the outgroup. The scale bar indicates the expected number of amino acid substitutions per site. Support values indicated on the nodes by a black circle correspond to the result of the likelihood ratio-test (aLRT values). An asterisk indicates the only hemipteran receptor that was deorphanized so far, the receptor to the *A. pisum* alarm pheromone.

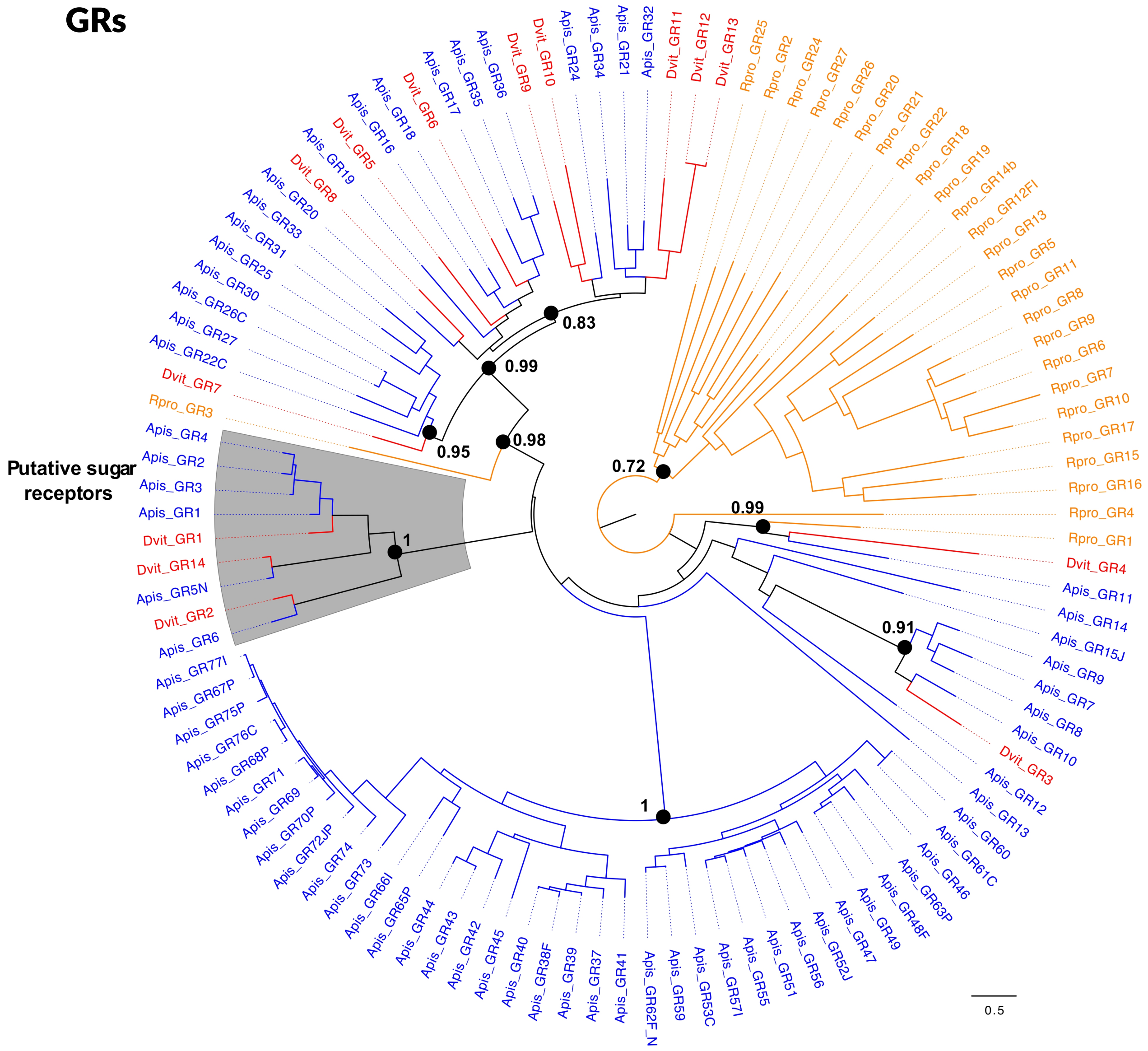


**Figure S17.** Maximum likelihood phylogeny of hemipteran GRs. The amino acid dataset included OR sequences from *D. vitifoliae* (Dvit), *R. prolixus* (Rpro) [34] and *A. pisum* (Apis) [35]. The tree was mid-point rooted. The scale bar indicates the expected number of amino acid substitutions per site. Support values indicated on the nodes by a black circle correspond to the result of the likelihood ratio-test (aLRT values). The gray clade indicates putative sugar receptors [35].

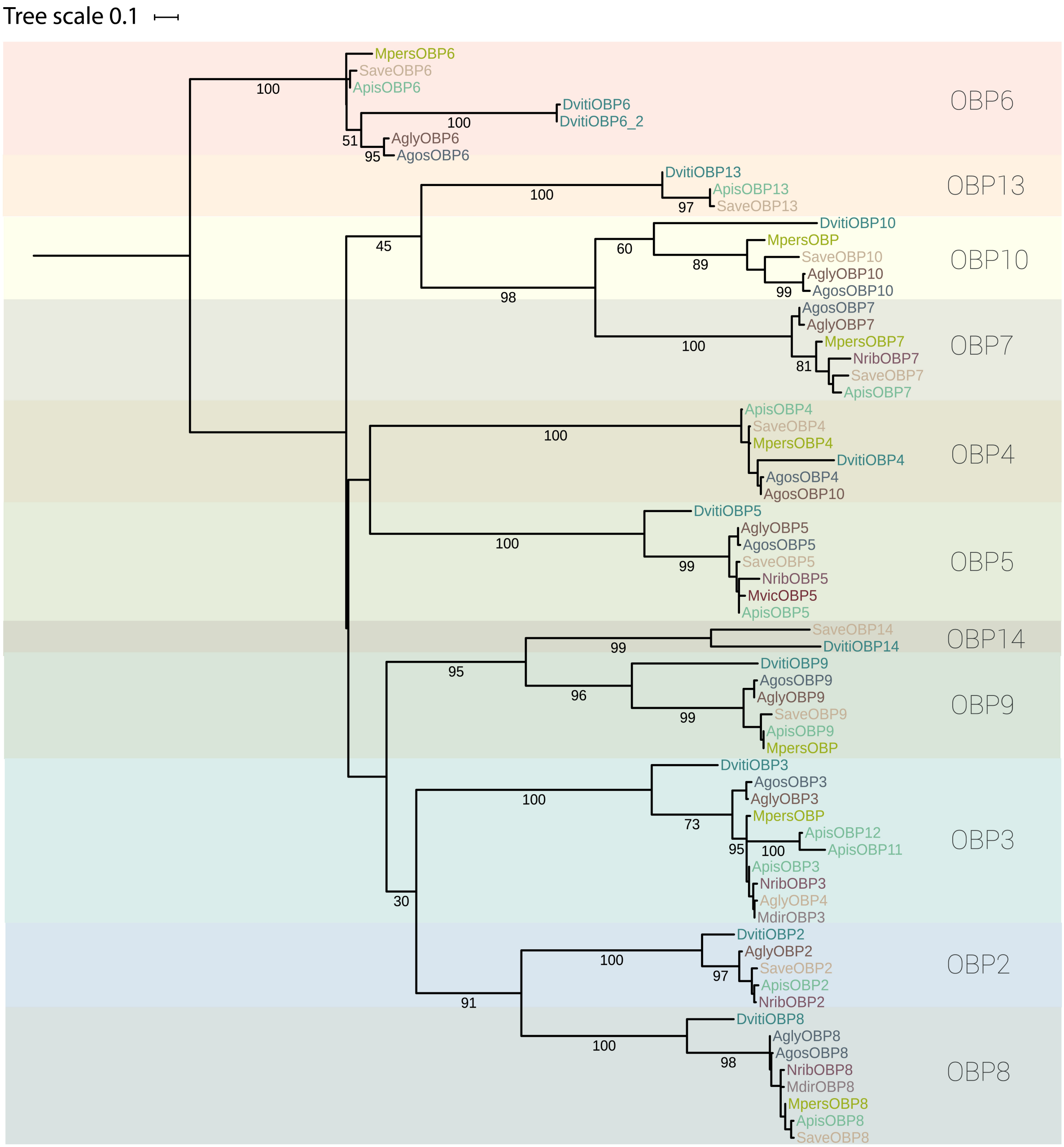


**Figure S18.** Phylogenetic analysis of the full length OBPs from phylloxera and representative aphid species. Each orthogroup is shaded in a different colour.

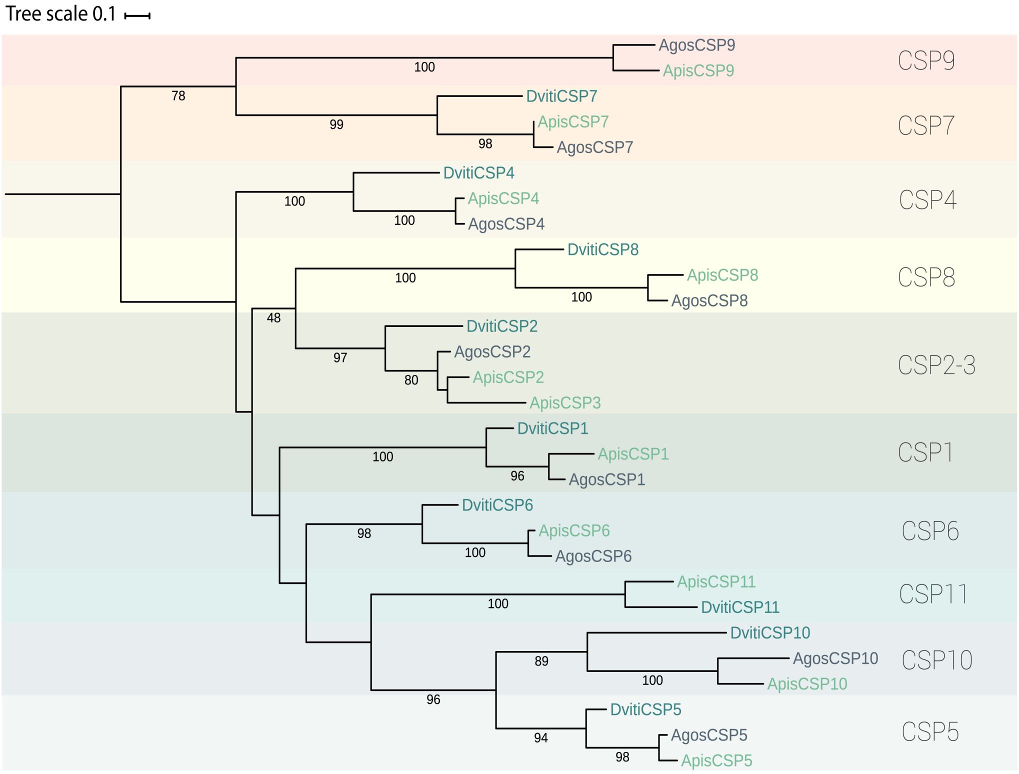


**Figure S19.** Phylogenetic relationships of the full length CSPs identified in phylloxera and some representative aphid species. Each orthogroup is shaded in a different colour.

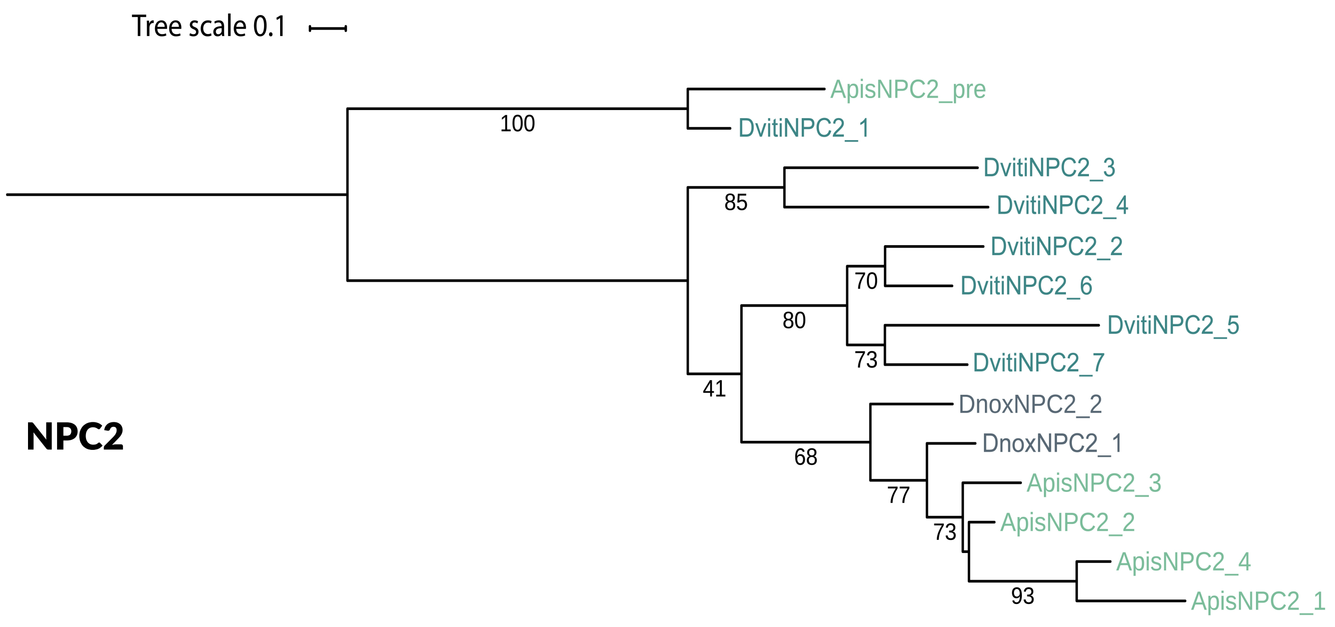


**Figure S20.** Phylogenetic relationships of the full length NPC2s identified in phylloxera and some representative aphid species.

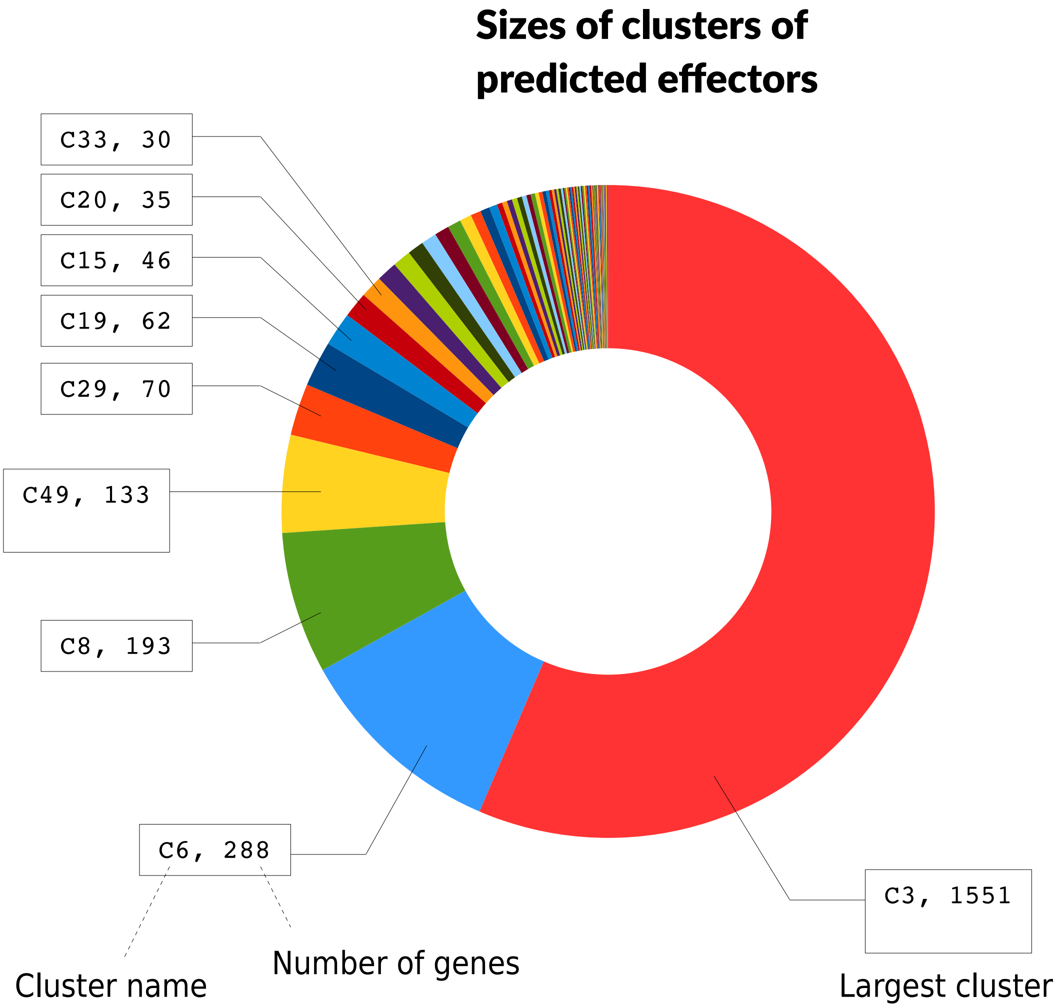


**Figure S21.** Distribution of siezs of clusters for putative effectors. Surface is proportional to cluster size, n=78 clusters. Details are given for the largest clusters (≥ 30 members), which totalize 87.6% of predicted effectors: cluster number and cluster size, separated by commas.

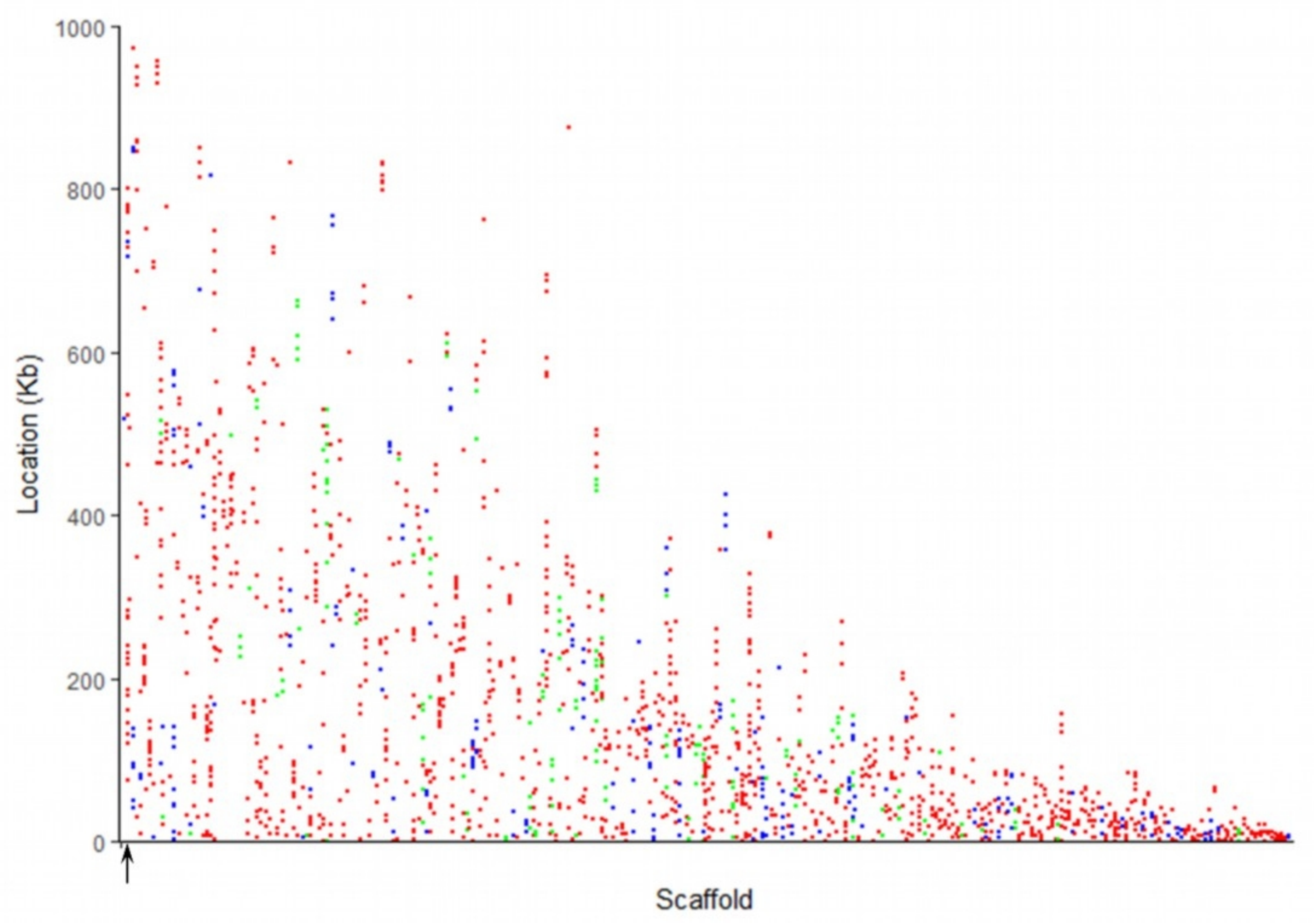


**Figure S22.** Predicted effectors of the three largest clusters (cluster numbers 3, 6, and 8) form tandem arrays on scaffolds. Each dot represents one gene. Only scaffolds (n=351) with ≥2 genes from a same cluster are shown (X-axis), and the scaffold length, if longer, is shown to the first 1000-kb (Y-axis). The arrow points to Scaffold #2 where 24 genes from the largest cluster (cluster 3) are located within an 800-kb region. Colors are coded such that cluster 3=red, 6=blue and 8=green.

**Table S1.** Genes of bacterial and fungal origin in *D. vitifoliae* and other insects. Horizontally transferred genes in *D. vitifoliae* and other insects [16,17]

| **Taxonomical group** | **Species** | **Genome DB** | **LD-carboxypeptidase (*LdcA*)**[1] | **Rare lipoprotein A (*rlpA*)**[1] | **N-acetylmuramoyl-L-alanine amidase (*amiD*)**[1] | **Phytoene desaturase**[2] | **Lycopene cyclase/ Phytoene synthase**[2] |
|---|---|---|---|---|---|---|---|
| Aphididae | *Acyrthosiphon pisum* | NCBI | 100168405 | 100163669, 100570300, 100167729, 100165005, 100570509 | 103308396 | 100169245 | 100161104 |
| | *Myzus persicae* | NCBI | 111041263 | 111033601, 111033591, 111033602, 111033592 | 111040498 | 111035763 | 111035746 |
| | *Diuraphis noxia* | NCBI | 107162751 | 107165933, 107165935 | 107164800 | 107165446 | 107165458 |
| | *Aphis glycines* | Aphidbase | AG000567 | AG002737, AG002738, AG002739 | no hit | AG002135 | AG002132 |
| | *Rhopalosipum padi* | Aphidbase | Rpa16117 | Rpa06900, Rpa06899, Rpa06898 | no hit | no hit | Rpa14780 |
| Phylloxeridae | *Daktulosphaira vitifoliae* | Aphidbase | no hit | no hit | no hit | DV3003641 | DV3003639 |
| Adelgidae | *Adelges cooleyi, Adelges laricis* | NA | NA | NA | NA | JN022785, JN022784 | not confirmed |
| Psyllidae | *Diaphorina citri* | NCBI | no hit | no hit | 103506806 | no hit | no hit |
| Aphalaridae | *Pachypsylla venusta* | NCBI | no hit | no hit | no hit | no hit | no hit |
| Aleyrodidae | *Bemisia tabaci* | NCBI | no hit | no hit | no hit | no hit | no hit |
| Other insects | *Habropoda spp, Rhodnius prolixus, Blatella germanica, Drosophila spp.* | i5K | no hit | inconclusive | no hit | no hit | no hit |

Putative homologs to genes of bacterial and fungal origin found by BLAST: [1] Gene of bacterial origin, [2] Gene of fungal origin , NA: no published genome

**Table S2.** Statistic results of the major superfamilies belonging to long terminal repeats (LTR) and terminal inverted repeats (TIR) orders. *Gypsy* and *hAT* elements are the most represented LTR and TIR superfamilies, respectively.

| Classes | Order | Super-families | Number of clusters | Total copy number | Coverage (% of the Genome) |
|---|---|---|---|---|---|
| Class I | LTR | *Copia* | 10 | 857 | 0.13 |
| | | *Gypsy* | 259 | 14.577 | 2.88 |
| | | *Bel/Pao* | 128 | 9.305 | 1.25 |
| | | *ERV* | 10 | 519 | 0.12 |
| Class II | TIR | *Tc1-mariner* | 111 | 5.996 | 0.71 |
| | | *piggyBac* | 21 | 1.268 | 0.14 |
| | | *hAT* | 370 | 23.003 | 3.25 |
| | | *CACTA* | 23 | 1672 | 0.18 |
| | | *Merlin* | 1 | 42 | 0.004 |
| | | *Transib* | 13 | 1.057 | 0.11 |
| | | *PIF-Harbinger* | 20 | 1.506 | 0.19 |
| | | *P* | 10 | 889 | 0.1 |
| | | *Mutator* | 17 | 1.053 | 0.17 |

**Table S3.** GO enrichment of genes duplicated at different ancestral nodes on the branch leading to graphe phylloxera (Aphidomorpha; Phylloxeroidea). From more basal to more terminal, Sternorrhyncha, Psyllidae + Aphidomorpha, and Aphidomorpha. Node, GO category (biological process, molecular function and cellular component), GO term, p-value of enrichment and putative functions are indicated.

**GO enrichment genes duplicated at different nodes**

| Node | #overlist | term | term_level | adj. pvalue | name |
|---|---|---|---|---|---|
| **1. Sternorrhyncha** | | | | | |
| Molecular_function | 2 | GO:0005515 | 1 | 5.54E-14 | structural constituent of ribosome |
| Cellular_component | 2 | GO:0005737 | 1 | 6.96E-04 | cytoplasm |
| **2. Psyllidae + Aphidomorpha** | | | | | |
| Molecular_function | 2 | GO:0003964 | 1 | 1.95E-04 | RNA-directed DNA polymerase activity |
| **3. Aphidomorpha** | | | | | |
| Molecular_function | 2 | GO:0003735 | 1 | 2.13E-04 | structural constituent of ribosome |
| | 2 | GO:0005488 | 1 | 2.47E-08 | binding |
| | 2 | GO:0005515 | 1 | 7.86E-83 | protein binding |
| Cellular_component | 2 | GO:0005622 | 1 | 3.49E-06 | intracellular |
| | 2 | GO:0005634 | 1 | 1.66E-09 | nucleus |
| | 2 | GO:0005737 | 1 | 1.51E-28 | cytoplasm |
| | 2 | GO:0005739 | 1 | 2.60E-07 | mitochondrion |
| | 2 | GO:0005783 | 1 | 7.63E-04 | ndoplasmic reticulum |
| | 2 | GO:0005811 | 1 | 1.37E-05 | lipid droplet |
| | 2 | GO:0005829 | 1 | 1.93E-10 | cytosol |
| | 2 | GO:0005875 | 1 | 2.44E-06 | microtubule associated complex |
| | 2 | GO:0005886 | 1 | 2.37E-07 | plasma membrane |
| | 2 | GO:0005887 | 1 | 6.65E-06 | integral component of plasma membrane |
| | 2 | GO:0012505 | 1 | 1.75E-04 | endomembrane system |
| | 2 | GO:0016020 | 1 | 5.11E-05 | membrane |
| Biological Process | 2 | GO:0006350 | 1 | 5.85E-05 | transcription, DNA-templated |
| | 2 | GO:0006355 | 1 | 1.61E-04 | regulation of transcription, DNA-templated |
| | 2 | GO:0006412 | 1 | 1.61E-04 | translation |
| | 2 | GO:0006468 | 1 | 1.37E-05 | protein phosphorylation |
| | 2 | GO:0006508 | 1 | 9.79E-09 | proteolysis |
| | 2 | GO:0019233 | 1 | 4.41E-11 | sensory perception |
| | 2 | GO:0022008 | 1 | 1.49E-11 | neurogenesis |
| | 2 | GO:0048477 | 1 | 5.42E-04 | oogenesis |
| | 2 | GO:0055114 | 1 | 4.34E-06 | oxidation-reduction process |

**Table S4.** Metabolic gaps in the *D. vitifoliae* reaction network. EC number, Gene ID, and type of annotation. Gene ID's linked by "and" are all required for function. Annotation categories are defined as follows: (i) *New gene prediction*: the gene was not called as part of the initial set of gene predictions. The gene was called, annotated, and is now part of the new official gene set, (ii) *New gene annotation*: The gene was called in the initial set of gene prediction but was not assigned any function by automated annotation. Function was assigned as a result of gap filling, and (iii) *Modified existing gene association*: the gene was called and assigned a function, gap filling identified an additional or alternative gene function.

| Enzyme Name | EC Number | Gene ID(s) | Annotation Category |
|---|---|---|---|
| 4-phosphopantotheoylcysteine decarboxylase | 4.1.1.36 | DV3025962.1 | New gene prediction |
| nicotinamidase | 3.5.1.19 | DV3000063 | New gene annotation |
| L-serine ammonia-lyase | 4.3.1.17 | DV3002372 | Modified existing gene association |
| cysteine synthase | 2.5.1.47 | DV3002954 | Modified existing gene association |
| glycerate 3-kinase | 2.7.1.31 | DV3011431 | Modified existing gene association |
| glycine hydroxymethyltransferase | 2.1.2.1 | DV3018664 | Modified existing gene association |
| glutamine—fructose-6-phosphate transaminase (isomerizing) | 2.6.1.16 | DV3002742 | Modified existing gene association |
| enoyl-CoA hydratase | 4.2.1.17 | DV4015045 | Modified existing gene association |
| 2-(1,2-epoxy-1,2-dihydrophenyl) acetyl-CoA isomerase | 5.3.3.18 | DV3008326 | Modified existing gene association |
| 3-oxo-5,6-didehydrosuberyl-CoA thiolase | 2.3.1.223 | DV3017937 | Modified existing gene association |
| spermine synthase | 2.5.1.22 | DV3017954 | Modified existing gene association |
| 3-hydroxybutyryl-CoA epimerase | 5.1.2.3 | DV3015045 | Modified existing gene association |
| dodecenoyl-CoA isomerase | 5.3.3.8 | DV3015045 | Modified existing gene association |
| fatty-acyl-CoA synthase | 2.3.1.86 | DV3018512, DV3000627, DV3004693 | Modified existing gene association |
| nicotinamide phosphoribosyltransferase | 2.4.2.12 | DV3004539 | Modified existing gene association |
| dihydropyrimidine dehydrogenase | 1.3.1.1 | DV3010281 | Modified existing gene association |
| pimelyl-ACP methyl ester esterase | 3.1.1.85 | DV3004923 | Modified existing gene association |
| dihydropyrimidine dehydrogenase | 1.3.1.1 | DV3010281 | Modified existing gene association |
| ditrans,polycis-undecaprenyl-diphosphate synthase | 2.5.1.31 | DV3017386 | Modified existing gene association |
| oxoglutarate dehydrogenase | 1.2.4.2 | DV3002078 and (DV3009715-PA or DV3006787) and DV3008581 | Modified existing gene association |
| tripeptide aminopeptidase | 3.4.11.4 | DV3012927 | Modified existing gene association |
| glycine dehydrogenase | 1.4.4.2 | DV3008581 and DV3001871 and DV3000698 and (DV3005511 or DV3020276) | Modified existing gene association |
| NADPH dehydrogenase | 1.6.99.1 | DV3017563 | Modified existing gene association |
| protein phosphatase methylesterase-1 | 3.1.1.89 | DV3009888 | Modified existing gene association |
| versatile peroxidase | 1.11.1.16 | DV3001833, DV3001185, DV3010201, DV3000879 | Modified existing gene association |
| acetaldehyde dehydrogenase | 1.2.1.10 | DV3000458, DV3009670, DV3010601, DV3008252 | Modified existing gene association |
| acetaldehyde dehydrogenase | 1.2.1.10 | DV3000458, DV3009670, DV3010601, DV3008252 | Modified existing gene association |
| glycerol-3-phosphate dehydrogenase | 1.1.5.3 | DV3012446 | Modified existing gene association |
| 1-pyrroline-5-carboxylate dehydrogenase | No EC | DV3010601 | Modified existing gene association |

**Table S5.** Functional annotation of metabolic genes in the Phylloxera, *Myzus persicae* and *Acyrthosiphon pisum* genomes. [1] Number of unique and complete numerical designations within the Enzyme Commission (EC) classification system for enzymes and their catalytic reactions. [2] comparison among the three species (Phylloxera, *Myzus persicae* and *Acyrthosiphon pisum*).

| **Species** | ***Daktulosphaira vitifoliae*** | ***Myzus persicae (G006)*** | ***Acyrthosiphon pisum*** |
|---|---|---|---|
| Gene set ID | OGS v3.2 | TGAC Annotation 1.0 | OGS v2.1b |
| CycADS Database ID | DakviCyc | MyzpeCyc | AcypiCyc |
| Polypeptides | 25840 | 24849 | 36228 |
| Pathways | 319 | 319 | 307 |
| Enzymatic reactions | 2377 | 2384 | 2339 |
| Enzymes | 3182 | 4646 | 5087 |
| Compounds | 1598 | 1603 | 1637 |
| EC[1] present in the genome | 1097 | 1115 | 1169 |
| EC unique to this genome[2] | 66 | 34 | 116 |
| EC missing only in this genome[2] | 71 | 21 | 49 |

**Table S6.** Identification of genes belonging to the TOLL pathway in *D. vitifoliae* based on the annotation available for *D. melanogaster.*

| **UniProt ID** | **Flybase ID** | **name** | **Sequences producing best significant alignments** | **E-value** | **score** |
|---|---|---|---|---|---|
| Q03017 | FBgn0000250 | cact | DV3003678.2-PA gene=Cactus | 118 | 2.00E-29 |
| P98149 | FBgn0011274 | dif | DV3014881.2-PA gene=Dorsal | 271 | 1.00E-79 |
| P15330 | FBgn0260632 | dl | DV3014881.2-PA gene=Dorsal | 454 | 3.00E-144 |
| Q9VVR4 | FBgn0040322 | gnbp2 | DV3010589-PA gene=DV3010589 | 125 | 2.00E-30 |
| Q7K105 | | myd88 | DV3005273.2-PA gene=myeloid differentiation primary response 88 | 141 | 3.00E-37 |
| Q05652 | FBgn0010441 | pll | DV3016029-PA gene=DV3016029 | 296 | 5.00E-92 |
| Q9VWU1 | FBgn0030926 | psh | DV3022332.2-PA gene=predicted effector 1928-5000 | 166 | 1.00E-46 |
| Q9VCJ8 | | Spe | DV3019909-PA gene=DV3019909 | 177 | 9.00E-51 |
| P48607 | | Spz | DV3006862.2-PA gene=spatzle | 87 | 1.00E-19 |
| P08953 | FBgn0262473 | toll | DV3003707.2-PA gene=Toll | 435 | 1.00E-134 |
| P22812 | | tube | DV3008856-PA gene=DV3008856 | 67.8 | 3.00E-12 |

**Table S7.** Identification of genes belonging to the IMD pathway in *D. vitifoliae* based on the annotation available for *D. melanogaster* (except for no-hit genes, e.g. Q8IPC3).

| **UniProt ID** | **Flybase ID** | **name** | **in/out** | **Sequences producing best alignments** | **E-value** | **score** |
|---|---|---|---|---|---|---|
| Q9VS59 | FBgn0082598 | akirin | in | DV3005216-PA gene=DV3005216 | 108 | 5.00E-29 |
| P35128 | FBgn0000173 | ben | in | DV3001602-PA gene=DV3001602 | 285 | 6.00E-99 |
| P92208 | FBgn0000229 | bsk | in | DV3005807-PA gene=DV3005807 | 648 | 0.00E+00 |
| P09085 | FBgn0000251 | cad | in | DV3019704.2-PA gene=caudal-3 | 135 | 5.00E-36 |
| Q7JUP3 | FBgn0034068 | casp | in | DV3001972-PA gene=DV3001972 | 385 | 5.00E-124 |
| Q24311 | FBgn0015509 | Cul1 | in | DV3006657-PA gene=DV3006657 | 963 | 0 |
| Q8IPC3 | FBgn0032210 | CYLD | out | | | |
| P36192 | FBgn0010385 | def | out | | | |
| Q24307 | FBgn0015247 | Diap2 | in | DV3009949-PA gene=DV3009949 | 228 | 6.00E-68 |
| Q8SWW8 | FBgn0260866 | dnr1 | in | DV3014323.2-PA gene=E3 ubiquitin-protein ligase MYLIP | 207 | 5.00E-59 |
| Q8IRY7 | FBgn0020381 | Dredd | in | DV3011341-PA gene=DV3011341 | 80.1 | 4.00E-16 |
| P25867 | FBgn0011217 | eff | in | DV3012664-PA gene=DV3012664 | 258 | 4.00E-89 |
| Q9V3B4 | FBgn0038928 | fadd | out | | | |
| Q23977 | | hep | in | DV3010686-PA gene=DV3010686 | 430 | 6.00E-136 |
| Q9VEZ5 | FBgn0024222 | ikkb | in | DV3002191-PA gene=DV3002191 | 111 | 2.00E-25 |
| Q7K4Z4.1 | FBgn0013983 | IMD | out | | | |
| P18289 | | jra | in | DV3016658-PA gene=DV3016658 | 158 | 5.00E-46 |
| P21525 | FBgn0001297 | kay | in | DV3008765-PA gene=DV3008765 | 60.1 | 2.00E-09 |
| NP_729319 | FBgn0011817 | nemo | in | DV3004747.2-PA gene=serine/threonine-protein kinase NLK | 650 | 0.00E+00 |
| NP_608422 | FBgn0031145 | ntf2 | in | DV3003037-PA gene=DV3003037 | 183 | 9.00E-160 |
| Q9GNK5 | FBgn0035976 | PGRP-LC | out | | | |
| NP_523776 | FBgn0040294 | posh | in | DV3011697-PA gene=DV3011697 | 180 | 2.00E-47 |
| Q94527 | FBgn0014018 | Rel | in | DV3014881.2-PA gene=Dorsal | 162 | 5.00E-41 |
| Q9VRP5 | FBgn0260936 | scny | in | DV3005278.2-PA gene=ubiquitin carboxyl-terminal hydrolase 36 | 344 | 5.00E-102 |
| NP_730815.2 | FBgn0037236 | skp2 | in | DV3014210-PA gene=DV3014210 | 150 | 1.00E-39 |
| Q4TZQ4 | | tab2 | out | | | |
| Q9V3Q6 | FBgn0026323 | tak1 | in | DV3004556.1-PA gene=TGF-beta activated kinase 1 | 333 | 1.00E-105 |
| NP_001286940.1 | FBgn0035601 | Uev1A | in | DV3004034-PA gene=DV3004034 | 231 | 5.00E-78 |

**Table S8.** Number of genes for different cuticular proteins sub-groups in *D. vitifoliae* (manual curation) and three aphid species. * Automatic annotation using CutProtFramPred, no manual curation.

| CP sub-groups | *D. vitifoliae* | *M. persicae* | *A. pisum* | *D. noxia** |
|---|---|---|---|---|
| CPAP1 | 10 | 6 | 10 | 10 |
| CPAP3 | 8 | 5 | 8 | 9 |
| CPCFC | 1 | 1 | 1 | 1 |
| CPF | 0 | 0 | 2 | 1 |
| Tweedle | 3 | 1 | 3 | 3 |
| RR-1 | 11 | 13 | 14 | 12 |
| RR-2 | 61 | 65 | 98 | 57 |
| **Total** | **94** | **91** | **137** | **93** |

**Table S9.** List and comparison of developmental genes in the *D. vitifoliae* and *A. pisum* genomes. In blue: genes not found. Part1:

| Classes | Gene Name | Number of copies | symbol | scaffold | start | end | strand |
|---|---|---|---|---|---|---|---|
| 1. Anterior-posterior axis formation | *bicoid* | 0 | - | | | | |
| | *exuperantia* | 1 | *exu* | 495 | 1614 | 6788 | - |
| | *torso* | 0 | - | | | | |
| | *torso-like protein* | 2 | *tsl-1* | 153 | 30556 | 46072 | + |
| | | | *tsl-2* | 409 | 270198 | 274152 | - |
| | *staufen* | 1 | *stau* | 163 | 172837 | 182839 | + |
| | *nos, vas, pum, tud* (in germ-cell development) | | | | | | |
| | *cad* (in Gap genes) | | | | | | |
| 2. Dorsal-ventral axis formation | *snail* | 0 | - | | | | |
| | *short gastrulation* | 1 | *sog* | 144 | 447524 | 458891 | - |
| | *twist* | 1 | *twi* | 177 | 236031 | 240028 | + |
| | *crossveinless* | 1 | *cv* | 544 | 39992 | 44630 | + |
| | *dorsal* | 1 | *dl* | 329 | 120811 | 127888 | - |
| | *cactus* | 1 | *cact* | 184 | 424807 | 430798 | - |
| | *Drop* | 1 | *Dr* | 186 | 378388 | 382070 | - |
| | *pannier* | 1 | *pnr* | 13 | 82138 | 91739 | - |
| | *ventral nervous system defective* | 1 | *vnd* | 215 | 353543 | 360483 | - |
| | *gurken* | 0 | - | | | | |
| | *intermediate neuroblasts defective* | 1 | *ind* | 4 | 738487 | 741356 | + |
| | *dachshund* | 2 | *dac-1* | 453 | 181439 | 209658 | + |
| | | | *dac-2* | 555 | 96556 | 116284 | - |
| | *tolloid* | 1 | *tld* | 35 | 713694 | 736887 | - |
| | *Race* | 0 | - | | | | |
| | *zen* (in Hox genes) | | | | | | |
| | *dpp,tkv* and *tgfbr1* (in TGF-beta signaling) | | | | | | |
| 3. Hox genes | *labial* | 1 | *lab* | 879 | 5876 | 25628 | - |
| | *proboscipedia* | 1 | *pb* | 612 | 11216 | 47474 | + |
| | *zerknüllt* | 0 | - | | | | |
| | *Deformed* | 1 | *Dfd* | 218 | 54308 | 64316 | - |
| | *Sex combs reduced* | 1 | *Scr* | 218 | 193293 | 211340 | - |
| | *fushi tarazu* | 1 | *ftz* | 218 | 218666 | 220045 | - |
| | *Antennapedia* | 1 | *Antp* | 218 | 347931 | 363442 | - |
| | *Ultrabithorax* | 1 | *Ubx* | 737 | 138256 | 215533 | + |
| | *abdominal-A* | 1 | *abd-A* | 205 | 209056 | 235709 | + |
| | *Abdominal-B* | 1 | *Abd-B* | 1302 | 25688 | 30488 | + |
| 4. Segmentation - Gap genes | *Kruppel* | 2 | *Kr-2* | 39 | 370715 | 377549 | + |
| | | | *Kr-1* | 185 | 101941 | 105133 | + |
| | *knirps* | 1 | *kni* | 324 | 58057 | 68980 | - |
| | *hunchback* | 1 | *hb* | 54 | 438888 | 443102 | + |
| | *tailless* | 1 | *tll* | 542 | 90047 | 94834 | - |
| | *empty spiracles* | 1 | *ems* | 449 | 88454 | 101741 | + |
| | *orthodenticle* | 1 | *otd* | 491 | 76397 | 102830 | - |
| | *Otx* | 0 | - | | | | |
| | *cap-n-collar* | 1 | *cnc* | 257 | 154479 | 208718 | + |
| | *caudal* | 3 | *cad-1* | 1046 | 6086 | 8793 | + |
| | | | *cad-2* | 2021 | 4829 | 6030 | - |
| | | | *cad-3* | 860 | 28796 | 43916 | - |
| | *knot* | 1 | *kn* | 26 | 46250 | 79571 | + |
| | *giant* | 0 | - | | | | |
| | *huckebein* | 0 | - | | | | |
| | *buttonhead* | 0 | - | | | | |

**Table S9.** Legend in the preceding page. Part2:

| Classes | Gene Name | Number of copies | symbol | scaffold | start | end | strand |
|---|---|---|---|---|---|---|---|
| 5. Segmentation - pair-rule genes | *hairy* | 1 | *h* | 507 | 43994 | 47821 | + |
| | *even skipped* | 1 | *eve* | 198 | 199497 | 207954 | - |
| | *runt* | 2 | *run-1* | 355 | 196452 | 205877 | - |
| | | | *run-2* | 937 | 5530 | 10812 | + |
| | *ftz* (in Hox genes) | | | | | | |
| | *odd skipped* | 2 | *odd-1* | 7 | 975573 | 978345 | + |
| | | | *odd-2* | 7 | 963819 | 965051 | - |
| | *sloppy paired* | 1 | *slp* | 534 | 181714 | 183575 | + |
| | *paird* | 0 | - | | | | |
| 6. Segmentation - segment polartiy genes | *engrailed* | 2 | *en-1* | 198 | 390864 | 402479 | - |
| | | | *en-2* | 198 | 318773 | 349713 | + |
| | *fused* | 1 | *fu* | 128 | 147432 | 155857 | - |
| | *gooseberry* | 1 | *gsb-1* | 433 | 215455 | 223347 | + |
| | | | *gooseberry-p1* | 2833 | 1243 | 2220 | + |
| | *Wg, arm, pan* (in Wnt signaling) | | | | | | |
| | *hh, ptc, ci* (in Hh signaling) | | | | | | |
| 7. Eye development | *twin of eyeless* | 1 | *toy* | 145 | 350880 | 378445 | - |
| | *eyeless* | 2 | *ey-1* | 1555 | 1383 | 15325 | - |
| | | | *ey-2* | 928 | 37512 | 64135 | + |
| | *eyes absent* | 1 | *eya* | 50 | 376877 | 400438 | - |
| | *twin of eyegone* | 1 | *toe* | 1685 | 1339 | 2238 | + |
| | *eyegone* | 1 | *eyg* | 10314 | 116 | 317 | - |
| | *sine oculis* | 1 | *so* | 499 | 121303 | 141266 | + |
| | *Optix* | 1 | *Optix* | 268 | 250760 | 268797 | - |
| | *lozenge* | 1 | *lz* | 289 | 178730 | 205388 | - |
| | *sevenless* | 1 | *sev* | 273 | 158483 | 174481 | - |
| | *bride of sevenless* | 1 | *boss* | 105 | 230184 | 235144 | + |
| | *Son of sevenless* | 1 | *Sos* | 289 | 311036 | 314711 | + |
| | *downstream of receptor kinase* | 1 | *drk* | 338 | 440240 | 444008 | + |
| | *atonal* | 1 | *ato* | 77 | 694066 | 694585 | + |
| | *pointed* | 1 | *pnt* | 361 | 52652 | 75745 | - |
| | *hh* (in Hh signaling) | | | | | | |
| | *Wg, dpp* (in Wnt signaling) | | | | | | |
| | *dac* (in Dorsal-Ventral axis formation) | | | | | | |
| | *Dl, ser* (in Notch signaling) | | | | | | |
| | *Egfr* (in EGF signaling) | | | | | | |
| 8. Salivary gland genes | *fork head* | 1 | *fkh* | 26 | 453100 | 455934 | - |
| | *saliva* | 1 | *slv* | 83 | 278720 | 287383 | + |
| | *extradenticle* | 1 | *exd* | 1003 | 3191 | 27638 | - |
| | *homothorax* | 3 | *hth-1* | 277 | 99537 | 106214 | + |
| | | | *hth-2* | 143 | 163637 | 179124 | + |
| | | | *hth-3* | 143 | 189823 | 214685 | + |
| 9. Appendage developmental genes | *apterous* | 2 | *ap-1* | 98 | 599879 | 614498 | - |
| | | | *ap-2* | 98 | 670044 | 698588 | - |
| | *Distal-less* | 1 | *Dll* | 392 | 315693 | 354992 | - |
| | *dpp* (in Wnt signaling) | | | | | | |
| | *hh* (in Hh signaling) | | | | | | |
| | *en* (in segment polarity) | | | | | | |

**Table S9.** Legend in the preceding pages. Part3:

| Classes | Gene Name | Number of copies | symbol | scaffold | start | end | strand |
|---|---|---|---|---|---|---|---|
| 10. Nurogenesis and muscle | *nautilus* | 0 | - | | | | |
| | *nubbin* | 1 | *nub* | 134 | 572199 | 601225 | + |
| | *castor* | 1 | *cas* | 137 | 587896 | 638128 | - |
| | *achaete* | 0 | - | | | | |
| | *scute* | 0 | - | | | | |
| | *asense* | 0 | - | | | | |
| | *lethal of scute* | 0 | - | | | | |
| | *ato* (in eye development) | | | | | | |
| | *Dl and N* (in Notch signalling) | | | | | | |
| 11. Gem-cell development | *armitage* | 1 | *armi* | 7 | 347915 | 357173 | - |
| | *bruno 1* | 0 | - | | | | |
| | *cappuccino* | 1 | *capu* | 184 | 385770 | 404785 | + |
| | *cup* | 0 | - | | | | |
| | *mago nashi* | 2 | *mago-1* | 523 | 24376 | 27222 | + |
| | | | *mago-2* | 190 | 50787 | 55999 | + |
| | *oo18 RNA-binding protein* | 0 | - | | | | |
| | *spire* | 1 | *spir* | 49 | 567784 | 586723 | - |
| | *oskar* | 0 | - | | | | |
| | *valois* | 0 | - | | | | |
| | *vasa* | 4 | *vas-1* | 102 | 336827 | 346608 | - |
| | | | *vas-2* | 102 | 274123 | 283160 | - |
| | | | *vas-3* | 60 | 454821 | 463448 | - |
| | | | *vas-4* | 95 | 671350 | 676692 | + |
| | *tuder* | 1 | *tud* | 401 | 37741 | 41026 | + |
| | *stau* (in Anterior-posterior axis formation) | | | | | | |
| | *germ cell-less* | 0 | - | | | | |
| | *gustavus* | 1 | *gus* | 468 | 16847 | 33631 | - |
| | *pipsqueak* | 1 | *psq* | 25 | 668106 | 680064 | + |
| | *nanos* | 3 | *nos-1* | 389 | 136705 | 138652 | - |
| | | | *nos-2* | 43 | 211197 | 215297 | + |
| | | | *nos-3* | 300 | 209744 | 215053 | + |
| | *wunen* | 0 | - | | | | |
| | *wunen-2* | 0 | - | | | | |
| | *HMG Coenzyme A reductase* | 1 | *Hmgcr* | 61 | 204652 | 219637 | + |
| | *argonaute-3* | 2 | *ago-3-a* | 392 | 6496 | 23191 | + |
| | | | *ago-3-b* | 440 | 3272 | 26332 | - |
| | *aubergine* | 0 | - | | | | |
| | *piwi* | 2 | *piwi-1* | 227 | 119927 | 157232 | - |
| | | | *piwi-2* | 227 | 186276 | 215905 | - |
| | *pumilio* | 1 | *pum* | 120 | 907333 | 912298 | + |
| | *Tropomyosin 1* | 1 | *Tm1* | 35 | 143676 | 153089 | + |
| | *par-1* | 0 | - | | | | |
| | *zero population growth* | 0 | - | | | | |
| | *swallow* | 0 | - | | | | |
| | *polar granule component* | 0 | - | | | | |
| | *spindle E* | 1 | *spn-E* | 248 | 49438 | 77571 | + |

**Table S10, part1.** miRNAs predicted by miRDeep2 (Part1, 'known' miRNAs, Part2, 'novel' miRNAs). 'Known' miRNAs are miRNAs with orthologs reported from other species, while 'novel' miRNAs are specific to *D. vitifoliae*. Clusters were assigned based on sorted scaffold numbers while the number of each cluster member is assigned based on its position. Duplications were assigned based on the sorted given miRNA names and duplicates were assigned based on genomic position. Pairs of miRNAs encoded from both strands at the same genomic location are named Both# and the numbers are assigned based on the sorted given miRNA names; + and - is in reference to the scaffold in the assembly. Part1.

| miRNA name | provisional id | miRDeep2 score | total read count | mature read count | loop read count | star read count | significant randfold p-value | consensus mature sequence | consensus star sequence | consensus precursor sequence | precursor coordinate | Clusters (distance < 10 kb) | Duplications | Encoded by both strands | Topology |
|---|---|---|---|---|---|---|---|---|---|---|---|---|---|---|---|
| Dvit-bantam | scaffold200_11861 | 462260.7 | 906698 | 905984 | 25 | 689 | yes | ugagaucauugugaaagcuaau | ugguuuucacuuugaucugucaga | ugguuuucacuuugaucugucagauuuauguuuugccuuuucugagaucauugugaaagcuaau | scaffold200:126202..126266:+ | | | | IGR (Between DV3005276.2 and DV3005280.2) |
| Dvit-let-7 | scaffold30_3527 | 8998.2 | 17642 | 17512 | 1 | 129 | yes | ugagguaguugguuguauagu | cuauacaaucggcuaacuuuucu | ugagguaguugguuguauaguaccauauaaucaauuuggaacguacuauacaaucggcuaacuuuucu | scaffold30:608779..608847:- | Cluster7-1 | | | Exon (of DV3002867) |
| Dvit-miR-10 | scaffold218_12729 | 2865228 | 5620020 | 5538002 | 0 | 82018 | yes | uacccuguagauccgaauuugu | caaauucgguucuagagagguuu | uacccuguagauccgaauuuguuugaaaacggcgacaaauucgguucuagagagguuu | scaffold218:95780..95839:- | | | | 3UTR&Exon Conjunction (of DV3014923.2) |
| Dvit-miR-100 | scaffold30_3529 | 382.5 | 751 | 732 | 0 | 19 | no | gacccguagauccgaacuugug | caaguacgguucuagaggaca | gacccguagauccgaacuugugucguaaaauuuagcaucgcaaguacgguucuagaggaca | scaffold30:612021..612082:- | Cluster7-2 | | | Intron (of DV3002867) |
| Dvit-miR-1000 | scaffold280_15631 | 5 | 7063 | 7011 | 28 | 24 | yes | auauuguccugucacagcag | cugcuguugcgggacauuuuca | auauuguccugucacagcaguaaugucacauuuaugaaucguacugcuguugcgggacauuuuca | scaffold280:208680..208745:- | Cluster5-1 | | | IGR (Between DV3014985 and DV3014989.1) |
| Dvit-miR-124* | scaffold116_8112 | 984.2 | 1935 | 1426 | 0 | 509 | no | cgcauucuccguuggucaauuaug | uaaggcacgcggugaaugcca | cgcauucuccguuggucaauuauguguaaaauuauucuucauaaggcacgcggugaaugcca | scaffold116:76333..76395:+ | | | | Intron&Exon Conjunction (of DV3008513) |
| Dvit-miR-137 | scaffold88_7006 | 1002.5 | 1969 | 1639 | 0 | 330 | yes | uuauugcuugagaauacacguag | ccgcguauucuugggacauaaca | ccgcguauucuugggacauaacacauauuuaagacuguuauugcuugagaauacacguag | scaffold88:195796..195856:- | | | | IGR (Between DV3000981.2 and DV3000984.2) |
| Dvit-miR-13a | scaffold690_30149 | 65869.6 | 129194 | 128581 | 0 | 613 | yes | uaucacagccacuuugaugagc | caucaaaauggaugugaagug | caucaaaauggaugugaaguguacauuacaauauucauaucacagccacuuugaugagc | scaffold690:75678..75737:+ | Cluster12-3 | Duplication1-1 | | Intron (of DV3017631) |
| Dvit-miR-13b | scaffold690_30151 | 1041823.9 | 2043496 | 2042694 | 0 | 802 | no | uaucacagccguuuuugacaauu | uggucaaaucggaugugacgug | uggucaaaucggaugugacguguugauaugucuauucauaucacagccguuuuugacaauu | scaffold690:76517..76578:+ | Cluster12-4 | Duplication1-2 | | Intron (of DV3017631) |
| Dvit-miR-14 | scaffold368_19305 | 218285.6 | 428151 | 423581 | 61 | 4509 | yes | ucagucuuuuucucucuccuau | cggagagaguuaacaggcuuuggcu | cggagagaguuaacaggcuuuggcuguuuacauguuugucauuagucagucuuuuucucucuccuau | scaffold368:38036..38103:+ | | | | IGR (At scaffold end before DV3025882.1) |
| Dvit-miR-184a | scaffold1_17 | 179121.7 | 351332 | 349682 | 0 | 1650 | yes | uggacggagaacugauaagggc | ccuuaucauucuucgccuugu | ccuuaucauucuucgccuugugugcauuuuaaacaacuggacggagaacugauaagggc | scaffold1:352628..352687:+ | | Duplication2-1 | | IGR (Between DV3002342 and DV3002343) |
| Dvit-miR-184b | scaffold360_19068 | 59781 | 117250 | 116837 | 0 | 413 | yes | uggacggagaacugauaaaggu | cuuuaucauucucuugucuugu | cuuuaucauucucuugucuugugugauguuaaacuacuggacggagaacugauaaaggu | scaffold360:52323..52383:- | | Duplication2-2 | | Intron (of DV3010567) |
| Dvit-miR-190 | scaffold339_18126 | 5318.9 | 10425 | 10248 | 0 | 177 | yes | agauauguuugauauucuugguug | accgggaaucagacauauugua | agauauguuugauauucuugguuguaauauuugaaaucgaccgggaaucagacauauugua | scaffold339:80816..80877:+ | | | | Intron (of DV3005333) |
| Dvit-miR-252a | scaffold89_7026 | 3087 | 6049 | 6041 | 2 | 6 | yes | cuaaguacuagugccgcgggag | ccugcagcucaagugcuuauca | cuaaguacuagugccgcgggagugguuuccuauuuuguaucuccugcagcucaagugcuuauca | scaffold89:496123..496187:+ | Cluster13-2 | Duplication3-1 | | IGR (Between DV3005002 and DV3005004) |
| Dvit-miR-252b-1 | scaffold110_7879 | 16987.4 | 33320 | 32955 | 9 | 356 | yes | uaaguaguagcgccaacgguga | accugcguacuacugcuuacc | uaaguaguagcgccaacggugaauugaauuuaaauaugucaccugcguacuacugcuuacc | scaffold110:207217..207278:- | | Duplication3-2 | | IGR (At scaffold end after DV3015934 |
| Dvit-miR-252b-2 | scaffold89_7024 | 16987.8 | 33320 | 32955 | 9 | 356 | yes | uaaguaguagcgccaacgguga | accugcguacuacugcuuacc | uaaguaguagcgccaacggugaauuguauuuaaauaugucaccugcguacuacugcuuacc | scaffold89:488252..488313:+ | Cluster13-1 | Duplication3-3 | | IGR (Between DV3005002 and DV3005004) |
| Dvit-miR-263a | scaffold162_10057 | 80164.1 | 157230 | 152905 | 75 | 4250 | yes | aauggcacugaaagaauucacggg | cguaauucuucugugccgugcu | aauggcacugaaagaauucacgggguacucacauuuuuauaccgcccguaauucuucugugccgugcu | scaffold162:230133..230201:- | Cluster3-2 | Duplication4-1 | | IGR (Between DV3007646 and DV3007648) |
| Dvit-miR-263b | scaffold162_10055 | 704.2 | 1376 | 1325 | 7 | 44 | yes | cuuggcacuggaagaauucacaga | gugagucucucggugucaauua | cuuggcacuggaagaauucacagacugaauguuauaaagaucgugagucucucggugucaauua | scaffold162:221202..221266:- | Cluster3-1 | Duplication4-2 | | IGR (Between DV3007646 and DV3007648) |
| Dvit-miR-275 | scaffold59_5347 | 17100.7 | 33534 | 31009 | 66 | 2459 | yes | ucagguaccugaaguagcgcg | ggcugcugcaggcacuuuuagacu | ggcugcugcaggcacuuuuagacucuguauuguuugaugaagucagguaccugaaguagcgcg | scaffold59:130977..131040:+ | Cluster10-1 | | | IGR (Between DV3009288 and DV3009292) |
| Dvit-miR-2765 | scaffold109_7852 | 16999.3 | 33336 | 31358 | 146 | 1832 | yes | ugguaacuccaaaccauugccgg | cgcaaugguuggaaguuccuca | ugguaacuccaaaccauugccggugucuuguaauucuuuuguaucccgcaaugguuggaaguuccuca | scaffold109:139874..139942:+ | Cluster1-1 | | | IGR (Between DV3012389 and DV3012392.2) |
| Dvit-miR-276a | scaffold25_3139 | 1240309.1 | 2432805 | 2427679 | 72 | 5054 | yes | uaggaacuucauaccgugcucu | agcgagguauagaguuccuacg | agcgagguauagaguuccuacguucaaaaauauuucauuauucguaggaacuucauaccgugcucu | scaffold25:160833..160899:+ | | | Both1 + | IGR (Between DV3025716.1 and DV3004263) |
| Dvit-miR-276b | scaffold25_3174 | 4.7 | 20 | 18 | 0 | 2 | yes | uaggaacucuauaccucgcuag | cacgguaugaaguuccuacgaau | cacgguaugaaguuccuacgaauaaugaaauauuuuugaacguaggaacucuauaccucgcuag | scaffold25:160831..160895:- | | | Both1 - | IGR (Between DV3004260 and DV3004265) |
| Dvit-miR-278 | scaffold18_2623 | 12978.5 | 25448 | 19802 | 12 | 5634 | yes | ucggugggacuuucguucguuu | acgggcgaagguuucaucaauc | acgggcgaagguuucaucaaucguguuacauuaagcgugucggucggugggacuuucguucguuu | scaffold18:449212..449277:+ | | | | IGR (Between DV3011526 and DV3011528) |
| Dvit-miR-279a | scaffold505_24573 | 153621.8 | 301315 | 295123 | 46 | 6146 | yes | ugacuagauccacacucaucc | augggugugcgucuugugacaug | augggugugcgucuugugacaugcuuuuuauauuuauucgacaugacuagauccacacucaucc | scaffold505:59622..59686:+ | | Duplication5-1 | | Exon (of DV3017081) |
| Dvit-miR-279b | scaffold250_14514 | 68331.5 | 134024 | 129264 | 118 | 4642 | yes | ugacuagaucuacacucauca | cguggguguucuucuagucuaca | cguggguguucuucuagucuacacuaaguaaauaaguuugugacuagaucuacacucauca | scaffold250:223628..223689:- | | Duplication5-2 | | Intron (of DV3016322.1) |
| Dvit-miR-281 | scaffold277_15541 | 129064.9 | 253155 | 246494 | 1 | 6660 | yes | aagagagcuauccgucgacagu | ugucauggaguugcucucuu | aagagagcuauccgucgacaguauaaugauugacuacacugucauggaguugcucucuu | scaffold277:112571..112630:- | | | | 3UTR (of DV3015052) |
| Dvit-miR-29 | scaffold281_15796 | 7141.8 | 14003 | 13684 | 39 | 280 | yes | uagcaccauuugaaaccaguac | acugguuuuuuuuggugggguaga | acugguuuuuuuuggugggguagacuaaaaauguugaacucaucuagcaccauuugaaaccaguac | scaffold281:267179..267244:- | | | | Intron (of DV3006964) |
| Dvit-miR-2a | scaffold690_30153 | 79686.9 | 156302 | 156243 | 0 | 59 | yes | ucacagccagcuuugaugagcg | cucacaaagcaacugugaaaug | cucacaaagcaacugugaaauguagauauugaagaaucauaucacagccagcuuugaugagcg | scaffold690:76626..76689:+ | Cluster12-5 | Duplication6-1 | | Intron (of DV3017631) |
| Dvit-miR-2b | scaffold690_30155 | 51584.7 | 101174 | 101079 | 92 | 3 | yes | uaucacagccagcuuuguaug | aucaaagugguagugaaac | aucaaagugguagugaaacguucuauuuucuuaauuauacauuaauucguaucacagccagcuuuguaug | scaffold690:76757..76827:+ | Cluster12-6 | Duplication6-2 | | Intron (of DV3017631) |
| Dvit-miR-2c | scaffold690_30147 | 77807.6 | 152615 | 147957 | 24 | 4634 | yes | ucacagccagcuuugaugagca | cgucaaagcgggcugucguaga | cgucaaagcgggcugucguagaguguuaacuguguuugacuaucacagccagcuuugaugagca | scaffold690:75558..75622:+ | Cluster12-2 | Duplication6-3 | | Intron (of DV3017631) |
| Dvit-miR-3016 | scaffold57_5279 | 148603.9 | 291474 | 289467 | 55 | 1952 | yes | auugguaacacucaacgucuuuag | aaagauguuuuuguugccuuug | auugguaacacucaacgucuuuagcauguuauuaaugaaagccuaaagauguuuuuguugccuuug | scaffold57:345463..345529:- | Cluster9-4 | | | IGR (Between DV3005077.2 and DV3005080.2) |
| Dvit-miR-3024a-1 | scaffold299_16540 | 4.5 | 101774 | 101774 | 0 | 0 | yes | ucuuugggauuuaagagagc | uaucuagaauuccaaaau | ucuuugggauuuaagagagcagguuuagauaauuaauaaaauuaauuaccagcuaucuagaauuccaaaau | scaffold299:20950..21021:+ | Cluster6-4 | Duplication7-1 | | IGR (Between DV3005472 and DV3005476) |
| Dvit-miR-3024a-2 | scaffold57_5281 | 4.2 | 101774 | 101774 | 0 | 0 | yes | ucuuugggauuuaagagagc | uaucuugaauuucaaaau | ucuuugggauuuaagagagcagguuuuaaguugaaauaccagcuaucuugaauuucaaaau | scaffold57:345578..345640:- | Cluster9-5 | Duplication7-4 | | IGR (Between DV3005077.2 and DV3005080.2) |
| Dvit-miR-3024b | scaffold299_16546 | 1.4 | 64 | 64 | 0 | 0 | yes | ucuuugggauuuccagcgagcug | gcucuuuuaaauuccaagaua | ucuuugggauuuccagcgagcuggucaaaauuaaauuaaauuaucagcucuuuuaaauuccaagaua | scaffold299:21931..21998:+ | Cluster6-7 | Duplication7-2 | | IGR (Between DV3005472 and DV3005476) |
| Dvit-miR-3024c | scaffold57_5273 | 4.4 | 24628 | 24079 | 124 | 425 | yes | ucuuugggauuucuagugggcu | aguuccuuagaaucccaggaua | ucuuugggauuucuagugggcugguauaaagaguauaaauugaccaguuccuuagaaucccaggaua | scaffold57:344857..344924:- | Cluster9-1 | Duplication7-3 | | IGR (Between DV3005077.2 and DV3005080.2) |
| Dvit-miR-3027-1 | scaffold3852_48070 | 40707.4 | 79838 | 63755 | 5 | 16078 | yes | ccagucuugcauuuauuccacu | ggggauaauuguagggcuuacu | ggggauaauuguagggcuuacuguucccuuuaaauacagccagucuugcauuuauuccacu | scaffold3852:363..424:- | | Duplication8-1 | | IGR (At scaffold end before DV3023864) |
| Dvit-miR-3027-2 | scaffold750_31842 | 40707.4 | 79838 | 63755 | 5 | 16078 | yes | ccagucuugcauuuauuccacu | ggggauaauuguagggcuuacu | ggggauaauuguagggcuuacuguucccuuuaaauacagccagucuugcauuuauuccacu | scaffold750:19392..19453:- | | Duplication8-2 | | Intron (of DV3018158.2) |
| Dvit-miR-3030a | scaffold299_16538 | 89043.7 | 174659 | 173621 | 81 | 957 | yes | agguaacggauuugcugugggga | ccaagcgaauuucgauacccu | agguaacggauuugcugugggauuauggacguuuaaauucccaagcgaauuucgauacccu | scaffold299:20846..20907:+ | Cluster6-3 | Duplication9-1 | | IGR (Between DV3005472 and DV3005476) |
| Dvit-miR-3030b | scaffold57_5283 | 99367.1 | 194904 | 180125 | 39 | 14740 | yes | agguaacggauuugcuauggga | ccaagcgaauccuguacccug | agguaacggauuugcuaugggaaagaucauguuaaauaucccaagcgaauccuguacccug | scaffold57:345705..345766:- | Cluster9-6 | Duplication9-2 | | IGR (Between DV3005077.2 and DV3005080.2) |
| Dvit-miR-3032a* | scaffold299_16533 | 233.7 | 452 | 331 | 1 | 120 | yes | ugacuagaauuauacuagca | guuaguauuguucuuaguaacg | guuaguauuguucuuaguaacgcgugaaaauuuaauucgugacuagaauuauacuagca | scaffold299:20327..20386:+ | Cluster6-1 | Duplication10-1 | | IGR (Between DV3005472 and DV3005476) |
| Dvit-miR-3032b* | scaffold57_5288 | 2.5 | 62216 | 60811 | 39 | 1366 | yes | ugacuagaauuuauacuacu | gguaguauaauucuuaguaaca | gguaguauaauucuuaguaacauugauuauuuaaguacugugacuagaauuuauacuacu | scaffold57:346050..346110:- | Cluster9-8 | Duplication10-2 | | IGR (Between DV3005077.2 and DV3005080.2) |
| Dvit-miR-3038* | scaffold226_12999 | 101.9 | 199 | 178 | 0 | 21 | yes | caaugcccuuagaaaucccaug | ugggguuucgauaggcauuuacc | ugggguuucgauaggcauuuaccuuguagauuaaaauggcaaugcccuuagaaaucccaug | scaffold226:166057..166118:+ | | | | IGR (Between DV3012084 and DV3012092) |
| Dvit-miR-3040* | scaffold109_7854 | 16709.1 | 32767 | 25694 | 12 | 7061 | yes | ugguaacuccaccaccgauugcg | cagccgguggugacuguucccaca | ugguaacuccaccaccgauugcguuuuuuuuuuuguuucaaaaaauuguuuuugaacucgcagccgguggugacuguucccaca | scaffold109:140047..140131:+ | Cluster1-2 | | | IGR (Between DV3012389 and DV3012392.2) |
| Dvit-miR-3041 | scaffold299_16549 | 15253.1 | 29910 | 23520 | 0 | 6390 | yes | uuaaagcuuugaugacgggau | ucccgaucgucaaaguuuuaggu | ucccgaucgucaaaguuuuagguuuuaauaauuuuuacaauacuuaaagcuuugaugacgggau | scaffold299:25866..25930:+ | Cluster6-9 | | | IGR (Between DV3005472 and DV3005476) |
| Dvit-miR-3049a | scaffold294_16228 | 654.6 | 1275 | 1134 | 0 | 141 | yes | ucgggaaggcaguugcggcggacu | uccguccaacuccuuuccguuu | ucgggaaggcaguugcggcggacugugugcuaauauauuaguccguccaacuccuuuccguuu | scaffold294:138647..138710:+ | | | Both2 + | IGR (Between DV3017398 and DV3017399) |
| Dvit-miR-3049b | scaffold294_16237 | 17.8 | 34 | 33 | 0 | 1 | yes | acggaaaggaguuggacggacu | uccgccgcaacugccuucccgauu | acggaaaggaguuggacggacuaauauauuagcacacaguccgccgcaacugccuucccgauu | scaffold294:138645..138708:- | | | Both2 - | IGR (Between DV3017397 and DV3017401.2) |
| Dvit-miR-305 | scaffold59_5350 | 42485.1 | 83333 | 83327 | 0 | 6 | yes | auuguacuucaucaggugcu | acuuguuggagugcaauu | auuguacuucaucaggugcucugguaaugcugaaaaucccggcacuuguuggagugcaauu | scaffold59:131209..131270:+ | Cluster10-2 | | | IGR (Between DV3009288 and DV3009292) |
| Dvit-miR-3053 | scaffold57_5275 | 38108.8 | 74741 | 74637 | 0 | 104 | yes | agguaacuuauucacuguggga | cacgggagaaugggggacccg | agguaacuuauucacugugggagugauauuaaauaucccacgggagaaugggggaccccg | scaffold57:345100..345160:- | Cluster9-2 | | | IGR (Between DV3005077.2 and DV3005080.2) |
| Dvit-miR-306 | scaffold118_8162 | 14123.3 | 27695 | 27580 | 0 | 115 | yes | ccagguacuaagugacuucug | aguuggcucuaaguaccucuaac | ccagguacuaagugacuucugagugaaauuaaaaauuauacaucaguuggcucuaaguaccucuaac | scaffold118:4838..4905:+ | Cluster2-2 | | | Intron (of DV3008114) |
| Dvit-miR-317 | scaffold223_12925 | 80142.2 | 157188 | 156710 | 0 | 478 | yes | ugaacacagcuggugguaucucagu | ugggaaccccgcgguguuaacu | ugggaaccccgcgguguuaacuugucauaauauuucgaugcauuauucgauaaaucagagugaacacagcuggugguaucucagu | scaffold223:172812..172897:+ | Cluster4-1 | | | IGR (Between DV3010857 and DV3010858) |
| Dvit-miR-34 | scaffold223_12930 | 3937.3 | 7715 | 6972 | 7 | 736 | yes | uggcagugugauuagcugguugug | cgaccacuaaacacauuccacu | uggcagugugauuagcugguuguguacaauaauuaauuuacuacucacgaccacuaaacacauuccacu | scaffold223:182494..182563:+ | Cluster4-2 | | | IGR (Between DV3010858 and DV3010861) |
| Dvit-miR-375 | scaffold466_23533 | 39629 | 77730 | 77479 | 0 | 251 | yes | uuuguucguucggcucgaguca | ccuugagccuagaggacaaau | ccuugagccuagaggacaaauuuguucauuauucgaaaucaauuuguucguucggcucgaguca | scaffold466:36696..36761:+ | | | | IGR (Between DV3015519 and DV3015520) |
| Dvit-miR-7-1 | scaffold419_21829 | 3.6 | 3875 | 3870 | 3 | 2 | yes | uggaagacuagugauuuuguu | caagaagucucuagucuucuua | uggaagacuagugauuuuguuguuuuaauguaaaucuuccuaacaagaagucucuagucuucuua | scaffold419:248568..248633:- | Cluster14-1 | Duplication11-1 | | IGR (Between DV3009493 and DV3009494) |
| Dvit-miR-7-2 | scaffold419_21831 | 3.6 | 3875 | 3870 | 3 | 2 | yes | uggaagacuagugauuuuguu | caagaagucucuagucuucuua | uggaagacuagugauuuuguuguuuuaauguaaaucuuccuaacaagaagucucuagucuucuua | scaffold419:331200..331265:- | Cluster14-2 | Duplication11-2 | | Intron (of DV3009500) |
| Dvit-miR-71 | scaffold690_30145 | 247771.7 | 485993 | 476228 | 0 | 9765 | yes | ucucacuaucuugucuuucaug | ugaaagacaugggguagugagaug | ugaaagacaugggguagugagauguaaaauugaaaccuaucucacuaucuugucuuucaug | scaffold690:75442..75502:+ | Cluster12-1 | | | Intron (of DV3017631) |
| Dvit-miR-8 | scaffold51_4888 | 2.9 | 1858877 | 1855094 | 0 | 3783 | yes | uaauacugucagguaaugaug | cgucuuacuuuggcaggauuaga | cgucuuacuuuggcaggauuagagugaauuaaaaugucuaauacugucagguaaugaug | scaffold51:268769..268828:- | | | | IGR (Between DV3006020.1 and DV3006022.1) |
| Dvit-miR-87a | scaffold61_5431 | 6612.8 | 12963 | 12225 | 0 | 738 | yes | gugagcaaaguuucaggugugc | gggccugacucguugcucaaccu | gggccugacucguugcucaaccugugcauuuauuauuaggugagcaaaguuucaggugugc | scaffold61:746000..746061:+ | Cluster11-1 | Duplication12-1 | | IGR (Between DV3002239 and DV3002243) |
| Dvit-miR-87b* | scaffold61_5434 | 5436.3 | 10662 | 5494 | 5 | 5163 | yes | cgccugaaacuugcuuugaccu | gugagcaaaguuucaggcgugu | cgccugaaacuugcuuugaccucgauuuauuuacgguaaggugagcaaaguuucaggcgugu | scaffold61:746138..746200:+ | Cluster11-2 | Duplication12-2 | | IGR (Between DV3002239 and DV3002243) |
| Dvit-miR-927a | scaffold232_13115 | 9268.2 | 18173 | 16703 | 3 | 1467 | yes | uuuagaauuccuacgcuuuacc | caaagcguuugaauuuuaaaac | uuuagaauuccuacgcuuuaccuauacuuuaucaaaauggcaaagcguuugaauuuuaaaac | scaffold232:92765..92827:- | | | Both3 - | IGR (Between DV3017604 and DV3017608) |
| Dvit-miR-927b | scaffold232_13107 | 262.7 | 515 | 510 | 0 | 5 | yes | uaaagcguaggaauucuaaaau | uuuaaaauucaaacgcuuugcc | uuuaaaauucaaacgcuuugccauuuugauaaaguauagguaaagcguaggaauucuaaaau | scaffold232:92767..92829:+ | | | Both3 + | IGR (Between DV3017605 and DV3017606) |
| Dvit-miR-929 | scaffold219_12752 | 272.9 | 527 | 517 | 0 | 10 | yes | aaauugacucuaguagggagu | cucucuaauggggucagauuga | aaauugacucuaguagggaguccuucuauuuaaugugacucucuaauggggucagauuga | scaffold219:138982..139042:+ | | | Both4 + | Intron (of DV3017036.2) |
| Dvit-miR-929* | scaffold219_12777 | 103.7 | 208 | 206 | 1 | 1 | yes | aaucugaccccauuagagaguc | cucccuacuagagucaauuug | aaucugaccccauuagagagucacauuaaauagaaggacucccuacuagagucaauuug | scaffold219:138981..139040:- | | | Both4 - | IGR (Between DV3017031 and DV3017045) |
| Dvit-miR-92a | scaffold394_20958 | 17301 | 33929 | 30562 | 0 | 3367 | yes | uauugcacaugucccggccaau | cggucgaugaccuguguaacuauu | cggucgaugaccuguguaacuauuguauaaauucgacaaauauugcacaugucccggccaau | scaffold394:30892..30954:- | Cluster8-2 | Duplication13-1 | | IGR (Between DV3012759 and DV3012761) |
| Dvit-miR-92b | scaffold394_20956 | 44458 | 87196 | 86355 | 18 | 823 | yes | uauugcacuugacccagccugc | agguuguguugugugcaaugug | agguuguguugugugcaaugugcguuuguucaauuaguauguuauuuucauauugcacuugacccagccugc | scaffold394:30727..30799:- | Cluster8-1 | Duplication13-2 | | IGR (Between DV3012759 and DV3012761) |
| Dvit-miR-971 | scaffold228_13039 | 4.5 | 264 | 151 | 0 | 113 | yes | uugguguucaaccuuacagu | acuuuaaguuugaucaccaagc | acuuuaaguuugaucaccaagccucagugcuaaauuguuugguguucaaccuuacagu | scaffold228:213624..213682:+ | | | | IGR (Between DV3006987 and DV3006988) |
| Dvit-miR-981 | scaffold511_24787 | 174.8 | 336 | 300 | 2 | 34 | yes | uucguuguugaugaaaccuuga | uggguuucucaauagcagaacc | uggguuucucaauagcagaaccuugacacagaauuaauaugguucguuguugaugaaaccuuga | scaffold511:140527..140591:+ | | | | IGR (At scaffold end after DV3014771) |
| Dvit-miR-993-1 | scaffold300_16668 | 7141 | 13998 | 6409 | 29 | 7560 | yes | gaagcucgucucuacagguaucu | cuacccuguagauccgggcu | cuacccuguagauccgggcuuuuguagaauaaugauuugcauuucagaagcucgucucuacagguaucu | scaffold300:336013..336082:- | | Duplication14-1 | | IGR (Between DV3010119 and DV3010120.2) |
| Dvit-miR-993-2 | scaffold7361_49118 | 7141 | 13998 | 6409 | 29 | 7560 | yes | gaagcucgucucuacagguaucu | cuacccuguagauccgggcu | cuacccuguagauccgggcuuuuguagaauaaugauuugcauuucagaagcucgucucuacagguaucu | scaffold7361:265..334:- | | Duplication14-2 | | IGR (no genes predicted on this scaffold) |
| Dvit-miR-996* | scaffold58_5324 | 16730.6 | 32815 | 20917 | 0 | 11898 | yes | acggguguagucuuuagugcacg | ugacuagaguuacacucguca | acggguguagucuuuagugcacguugauuuugaaauucgugacuagaguuacacucguca | scaffold58:495924..495984:+ | | | | Intron (of DV3006260.1) |
| Dvit-miR-998 | scaffold120_8289 | 169262.5 | 332007 | 331566 | 1 | 440 | no | uagcaccauggaauucagcug | acugaauuuuacgagugccgca | acugaauuuuacgagugccgcauguaauguaugucuuguagcaccauggaauucagcug | scaffold120:822838..822897:+ | | | | Intron (of DV3002008) |
| Dvit-miR-9a | scaffold138_9000 | 57466.7 | 112710 | 112447 | 202 | 61 | yes | ucuuugguuaucuagcuguauga | auaggucuaggugaccgaaguu | ucuuugguuaucuagcuguaugaguguaacguuucauugauucucauaggucuaggugaccgaaguu | scaffold138:295939..296006:+ | | Duplication15-1 | | Intron (of DV3016190) |
| Dvit-miR-9b | scaffold118_8160 | 10193.5 | 19989 | 19824 | 2 | 163 | yes | ucuuugguacucuagcuguagg | uaaagcuuuuguaccuaaguga | ucuuugguacucuagcuguagguauauuauaaguuggccuaaagcuuuuguaccuaaguga | scaffold118:3269..3330:+ | Cluster2-1 | Duplication15-2 | | IGR (At scaffold end before DV3008114) |
| Dvit-miR-iab-4 | scaffold205_12089 | 3.9 | 138 | 126 | 0 | 12 | yes | acguauacuaaauguauccug | cgguauaccuucaguauacguaac | acguauacuaaauguauccugagugcauuauaaacuguccgguauaccuucaguauacguaac | scaffold205:114943..115006:- | | | Both5 - | IGR (At scaffold end after DV3021311) |
| Dvit-miR-iab-4* | scaffold205_12082 | 356.8 | 698 | 491 | 0 | 207 | yes | uuacguauacugaagguauaccgga | aggauacauuuaguauacguaca | uuacguauacugaagguauaccggacaguuuauaaugcacucaggauacauuuaguauacguaca | scaffold205:114944..115009:+ | | | Both5 + | IGR (At scaffold after DV3025607.1) |

**Table S10, part2.** See preceding page for the legend.

| miRNA name | provisional id | miRDeep2 score | total read count | mature read count | loop read count | star read count | significant randfold p-value | consensus mature sequence | consensus star sequence | consensus precursor sequence | precursor coordinate | Clusters (distance < 10 kb) | Duplications | Encoded by both strands | Topology |
|---|---|---|---|---|---|---|---|---|---|---|---|---|---|---|---|
| Dvit-miR-x1 | scaffold10_1850 | 0.7 | 1289 | 1216 | 0 | 73 | yes | acugaucuguuuucuuacuuacu | uaaguaaggaaauggguaucu | uaaguaaggaaauggguaucuauuguuuucuuuguuuguaacaacugaucuguuuucuuacuuacu | scaffold10:1340790..1340856:- | | Duplication16-1 | | Intron (of DV3000834) |
| Dvit-miR-x2 | scaffold436_22300 | 3.3 | 8 | 4 | 0 | 4 | yes | auaagaaaacugguuucuauuguu | caauugaccuauuuccuuauuu | auaagaaaacugguuucuauuguuauuauuuauaacaauugaccuauuuccuuauuu | scaffold436:115648..115705:- | | Duplication16-2 | | IGR (Between DV3012678 and DV3012680.2) |
| Dvit-miR-x3a | scaffold335_18052 | 0.3 | 2718 | 2717 | 1 | 0 | yes | auugaccuguuuacuuacuuacu | uaaggaagaaagcagguuucaauug | uaaggaagaaagcagguuucaauuguucuuauuguuuguauuaauugaccuguuuacuuacuuacu | scaffold335:51676..51742:- | | Duplication16-3 | | IGR (Between DV3017612.2 and DV3017615) |
| Dvit-miR-x3b | scaffold451_22682 | 2 | 7137 | 7061 | 5 | 71 | yes | auugaccuguuuccuuacuuacu | uaaggaaacggauucuauuguu | uaaggaaacggauucuauuguucucauuguuuauaacaauugaccuguuuccuuacuuacu | scaffold451:116515..116576:+ | | Duplication16-4 | | IGR (At scaffold end after DV3019074) |
| Dvit-miR-x3c | scaffold1069_39320 | 1569.4 | 3079 | 3023 | 0 | 56 | yes | auugaccuguuugcuuacuuacu | uaaguuaggaaauggguuucu | uaaguuaggaaauggguuucuauuggucucauuauuuauaacaauugaccuguuugcuuacuuacu | scaffold1069:4840..4906:- | | Duplication16-5 | | IGR (At scaffold end before DV3021221) |
| Dvit-miR-x3d | scaffold46_4463 | 1.6 | 807 | 800 | 0 | 7 | yes | auugaccuguuuuguuacuuacu | aauaaggaaauggguuuuaauu | aauaaggaaauggguuuuaauuguuuugauuguaacaauugaccuguuuuguuacuuacu | scaffold46:592274..592334:+ | | Duplication16-6 | | IGR (Between DV3007574.2 and DV3007576) |
| Dvit-miR-x4 | scaffold699_30519 | 2429.6 | 4764 | 4745 | 0 | 19 | yes | auuuaccuguuuccuuacuuacu | caagcaaggaaacagguuuuuauug | caagcaaggaaacagguuuuuauuguuuuaauuguaacaauuuaccuguuuccuuacuuacu | scaffold699:44868..44930:- | | Duplication16-7 | | Intron (of DV3018739) |
| Dvit-miR-x5 | scaffold165_10150 | 1406.9 | 2758 | 2699 | 38 | 21 | yes | ccuauuuccuuacuuacugaaca | uucaguaaguaaguaaauaggu | uucaguaaguaaguaaauagguuucuauuguucucauuguuuguaacaaucuaccuauuuccuuacuuacugaaca | scaffold165:169283..169359:- | | Duplication16-8 | | IGR (Between DV3013994.2 and DV3024789.2) |
| Dvit-miR-x6a | scaffold14_2490 | 981.3 | 1923 | 1908 | 0 | 15 | yes | ccuguuuccuuacauacugaaca | uucuguaaguaaggaaaugggu | uucuguaaguaaggaaauggguucuauuguucucauaguuuuuaacaauggaccuguuuccuuacauacugaaca | scaffold14:435049..435124:- | | Duplication16-9 | | IGR (Between DV3005850 and DV3005853) |
| Dvit-miR-x6b | scaffold196_11780 | 2491 | 4885 | 4763 | 0 | 122 | yes | ccuguuuccuuacuuacugaaca | uucaguaaguaaggaaaugggu | uucaguaaguaaggaaauggguaucuaucguucuuauuguaaccuguuuccuuacuuacugaaca | scaffold196:524887..524952:+ | | Duplication16-10 | | IGR (Between DV3006661 and DV3006666) |
| Dvit-miR-x6c | scaffold620_28468 | 1.2 | 3051 | 2739 | 3 | 309 | yes | ccuguuuccuuauuuacugaaca | uguucaguaaguaaggaaac | uguucaguaaguaaggaaacgaguuucuauuguacuuauuauuuauaacaauucaccuguuuccuuauuuacugaaca | scaffold620:36783..36861:- | | Duplication16-11 | | Intron (of DV3017172) |
| Dvit-miR-x6d | scaffold255_14755 | 1.3 | 963 | 941 | 22 | 0 | yes | cuguuuucuuacuuacugaaca | uucagaaaguaagcaaacgau | uucagaaaguaagcaaacgauuucuauuguuuucauuguuuguaacaauuuaucuguuuucuuacuuacugaaca | scaffold255:521703..521778:+ | | Duplication16-12 | | Intron (of DV3006307.1) |
| Dvit-miR-x7 | scaffold38_4013 | 329.4 | 646 | 580 | 0 | 66 | yes | uuuccucacuuacugaacacc | uguucaguaaguaaggaaaa | uguucaguaaguaaggaaaaugguaaauuauuacaaacaauagaaauguguuuccucacuuacugaacacc | scaffold38:272660..272731:+ | | Duplication17-1 | | Intron (of DV3014139) |
| Dvit-miR-x8a | scaffold257_14807 | 1354.2 | 2657 | 2625 | 28 | 4 | yes | cccguuuccuuacuuacugaaca | uuuaguaagucaggacac | uuuaguaagucaggacacaggucaauuauuauaaacaaugaaaacaauagagacccguuuccuuacuuacugaaca | scaffold257:54910..54986:+ | | Duplication17-2 | | IGR (Between DV3014080 and DV3014083.1) |
| Dvit-miR-x8b | scaffold454_22782 | 177.1 | 348 | 159 | 0 | 189 | yes | ccguuuccuuaauuacugaaca | aucaguaaguaaggaaacagguc | aucaguaaguaaggaaacaggucagcuguuacaaacaauuacgauaauauaagaccguuuccuuaauuacugaaca | scaffold454:38274..38350:- | | Duplication17-3 | | Intron (of DV3013567) |
| Dvit-miR-x9 | scaffold75_6251 | 594.4 | 1167 | 1134 | 19 | 14 | yes | cuguuucuuuacuuacugaaca | uucaguaaguuuugaaauaggu | uucaguaaguuuugaaauagguaaauuguuauaaacaaugagaacauuagaaaucuguuucuuuacuuacugaaca | scaffold75:216285..216361:- | | Duplication17-4 | | IGR (Between DV3000430 and DV3000431) |
| Dvit-miR-x10 | scaffold2620_47495 | 9 | 53 | 51 | 0 | 2 | yes | guaaauaucaugauuguaaua | auuacgaaaucauuuauuuacag | guaaauaucaugauuguaauauuuugaaacaauuaucaauuacgaaaucauuuauuuacag | scaffold2620:1949..2012:- | | Duplication18-1 | | IGR (no genes predicted in this scaffold) |
| Dvit-miR-x10* | scaffold492_24214 | 37.2 | 90 | 39 | 0 | 51 | yes | uuaugaaaucauuuuauuuacag | guaaauaucaugauuguaaua | guaaauaucaugauuguaauauuuuugaaaaaauuaugaaauuaugaaaucauuuuauuuacag | scaffold492:232164..232228:+ | | Duplication18-2 | | Intron (of DV3011756.1) |
| Dvit-miR-x11 | scaffold280_15616 | 4748.2 | 9314 | 8921 | 0 | 393 | yes | auugggacaauggauuuauaauu | cuauaaauccauugucccaau | auugggacaauggauuuauaauucgguuuaaaauguacaacuauaaauccauugucccaau | scaffold280:218020..218081:+ | | | Both6 + | IGR (Between DV3014987 and DV3014988.2) |
| Dvit-miR-x11* | scaffold280_15634 | 7160.7 | 14044 | 4897 | 0 | 9147 | yes | uuauaaauccauugucccaaucg | auugggacaauggauuuauaguu | auugggacaauggauuuauaguuguacauuuuaaaccgaauuauaaauccauugucccaaucg | scaffold280:218018..218081:- | Cluster5-2 | | Both6 - | IGR (Between DV3014985 and DV3014989.1) |
| Dvit-miR-x12-1 | scaffold644_29283 | 43.7 | 83 | 79 | 0 | 4 | yes | auggcaucugugaucgaaugaa | ucauucgaucacagaugccaua | ucauucgaucacagaugccauaaaguuuuauacuuuauggcaucugugaucgaaugaa | scaffold644:56377..56435:+ | | | Both7 + | IGR (Between DV3019244 and DV3019248.2) |
| Dvit-miR-x12-2 | scaffold644_29288 | 43.8 | 83 | 79 | 0 | 4 | yes | auggcaucugugaucgaaugaa | ucauucgaucacagaugccaua | ucauucgaucacagaugccauaaaguauaaaacuuuauggcaucugugaucgaaugaa | scaffold644:56376..56434:- | | | Both7 - | IGR (Between DV3019246 and DV30192470 |
| Dvit-miR-x13a | scaffold299_16543 | 59387.5 | 116490 | 115890 | 0 | 600 | yes | guaaagauguuuuaacuacua | aguagcauaaaauaucuuuagc | aguagcauaaaauaucuuuagcuaguuaaaaguaucaaccuaguaaagauguuuuaacuacua | scaffold299:21511..21574:+ | Cluster6-6 | Duplication19-1 | | IGR (Between DV3005472 and DV3005476) |
| Dvit-miR-x13b | scaffold57_5278 | 61177.6 | 119996 | 118629 | 1 | 1366 | yes | guaaagauguuuuaacuacuc | aguagcaaaaagcaucuuuaacu | aguagcaaaaagcaucuuuaacuaguauauuauaguaaucuaguaaagauguuuuaacuacuc | scaffold57:345274..345337:- | Cluster9-3 | Duplication19-2 | | IGR (Between DV3005077.2 and DV3005080.2) |
| Dvit-miR-x14a | scaffold766_32636 | 2132.4 | 4183 | 4073 | 0 | 110 | yes | uguucuauaguuuuggcugcu | acagccagaucuguagaauaau | uguucuauaguuuuggcugcuuuacauguuaauaagaaacagccagaucuguagaauaau | scaffold766:35440..35500:+ | | Duplication20-1 | Both8 + | IGR (Between DV3015639 and DV3015640) |
| Dvit-miR-x14b | scaffold766_32637 | 7.1 | 23 | 17 | 0 | 6 | yes | uauucuacagaucuggcuguuu | gcagccaaaacuauagaacagc | uauucuacagaucuggcuguuucuuauuaacauguaaagcagccaaaacuauagaacagc | scaffold766:35438..35498:- | | Duplication20-2 | Both8 - | IGR (Between DV3015638 and DV3015641) |
| Dvit-miR-x15-1 | scaffold103_7495 | 104.8 | 206 | 203 | 0 | 3 | yes | ucaagaucguaauacaccggccg | ccuggguauuaccaucggcuga | ccuggguauuaccaucggcugauuuugauaccacucgaaucaagaucguaauacaccggccg | scaffold103:371948..372010:+ | | Duplication21-1 | | IGR (Between DV3006426.2 and DV3006430) |
| Dvit-miR-x15-2 | scaffold103_7497 | 104.8 | 206 | 203 | 0 | 3 | yes | ucaagaucguaauacaccggccg | ccuggguauuaccaucggcuga | ccuggguauuaccaucggcugauuuugauaccacucgaaucaagaucguaauacaccggccg | scaffold103:394935..394997:+ | | Duplication21-2 | | IGR (Between DV3006426.2 and DV3006430) |
| Dvit-miR-x16-1 | scaffold10_1455 | 94.2 | 189 | 79 | 0 | 110 | yes | guaaguaaaaauucaauuauug | auaguugaauuuuuauuuauuag | guaaguaaaaauucaauuauuguuuaaauaaauaauauaaaaauauuaauaguaauaaauaguugaauuuuuauuuauuag | scaffold10:447718..447798:- | | Duplication22-1 | | Intron (of DV3000764) |
| Dvit-miR-x16-2 | scaffold891_35621 | 79.5 | 161 | 79 | 0 | 82 | yes | guaaguaaaaauucaauuauug | auaguugaauuuuuauuuaauag | guaaguaaaaauucaauuauuguuuaaauaaauaauaugaaaauauuaauaguaauaaauaguugaauuuuuauuuaauag | scaffold891:16595..16675:- | | Duplication22-2 | | IGR (Between DV3019959 and DV3019961) |
| Dvit-miR-x17 | scaffold115_8091 | 462 | 904 | 406 | 0 | 498 | yes | uguuccaccgucauuuacuaauu | uuaguaaaucacggugggacacu | uguuccaccgucauuuacuaauuuuaacauuaguuuauauagucaaauuaguaaaucacggugggacacu | scaffold115:333811..333881:- | | Duplication23-1 | | IGR (Between DV3025277.1 and DV3002415.2) |
| Dvit-miR-x17* | scaffold88_7010 | 69.7 | 142 | 141 | 0 | 1 | yes | uuaguaaaucacagugggaca | acccacuaagauuuaccaauuu | acccacuaagauuuaccaauuuuaacauuauuuuauauuaucaaauuaguaaaucacagugggaca | scaffold88:629867..629933:- | | Duplication23-2 | | 3UTR (of DV3001018) |
| Dvit-miR-x18 | scaffold1_33 | 9.6 | 18 | 16 | 0 | 2 | yes | uuaaguauagcuauucauagga | uaugcauaaguuuuacuuaagca | uaugcauaaguuuuacuuaagcauguuguuuagauagcuuaaguauagcuauucauagga | scaffold1:698629..698689:+ | | | | Intron (of DV3002368) |
| Dvit-miR-x19 | scaffold1_47 | 68.2 | 133 | 78 | 0 | 55 | yes | acuuaauccccuuuagcucuga | agugcuaaaaggugauuaagucc | agugcuaaaaggugauuaaguccuuucaaauuacauggacuuaauccccuuuagcucuga | scaffold1:878591..878651:+ | | | | IGR (Between DV3002371.2 and DV3002376) |
| Dvit-miR-x20 | scaffold10_1058 | 252.8 | 497 | 492 | 0 | 5 | yes | ugauuuucuuuuucaauugggc | cccaauugaaaaagaaguucaua | ugauuuucuuuuucaauugggcauaauaauaauuucuucuacccaauugaaaaagaaguucaua | scaffold10:1288659..1288723:+ | | | | Intron (of DV3000829) |
| Dvit-miR-x21 | scaffold116_8129 | 39.1 | 80 | 40 | 0 | 40 | yes | aucgcucacgaaaggauguaag | uacauccauucaugagcaauuu | uacauccauucaugagcaauuucauuaagcauauuuuaaaucgcucacgaaaggauguaag | scaffold116:323231..323293:+ | | | | Intron (of DV3008536) |
| Dvit-miR-x22 | scaffold120_8346 | 4322 | 8476 | 8306 | 0 | 170 | yes | cggacagacugaguauggaccac | guucauacucagucuguucug | guucauacucagucuguucugaauuuucuuuucuauccggacagacugaguauggaccac | scaffold120:759788..759848:- | | | | IGR (Between DV3002003 and DV3002004) |
| Dvit-miR-x23 | scaffold1264_41708 | 2808.8 | 5508 | 5071 | 0 | 437 | yes | uacugauuuauguugguguaga | accaccaacauaauucgggggg | accaccaacauaauucggggggcauacuucuuugccuacugauuuauguugguguaga | scaffold1264:22211..22272:- | | | | IGR (Between DV3021919 and DV3021920.2) |
| Dvit-miR-x24 | scaffold155_9831 | 54.9 | 109 | 71 | 0 | 38 | yes | ugccuugaugacgacuguaaucg | uaugcgucguaaucaaguuaga | ugccuugaugacgacuguaaucgacguacauucucucaaggucauaugcgucguaaucaaguuaga | scaffold155:268368..268434:- | | | | Exon (of DV3009437) |
| Dvit-miR-x25 | scaffold158_9935 | 34.8 | 65 | 54 | 0 | 11 | yes | ucauuguuaugggccauauga | cuuauggcccauaccaaugaag | ucauuguuaugggccauaugagcguuaaaaagaauacauguauauaugugcucuuauggcccauaccaaugaag | scaffold158:122824..122898:- | | | | Intron (of DV3004923) |
| Dvit-miR-x26 | scaffold187_11467 | 88.9 | 178 | 169 | 0 | 9 | yes | aaaguuaauaguaauacggaacu | uucaaaauacuauuaauuuagu | aaaguuaauaguaauacggaacuguuucuucuccauuuucuuacaguucaaaauacuauuaauuuagu | scaffold187:125884..125952:- | | | | Intron (of DV3012210.1) |
| Dvit-miR-x27 | scaffold19_2729 | 14.2 | 32 | 28 | 0 | 4 | yes | ucuguccauuuuucauacccauu | gugggaauguagaacgggcacagcuca | gugggaauguagaacgggcacagcucauacuuguguacguauacucuguccauuuuucauacccauu | scaffold19:630363..630430:+ | | | | Intron (of DV3006837) |
| Dvit-miR-x28 | scaffold194_11717 | 47.1 | 88 | 87 | 0 | 1 | yes | gggaauguuuguaaaaugucaa | aauauuuuacaaauauuuuuu | aauauuuuacaaauauuuuuuguaaaaaaaaaaagggaauguuuguaaaaugucaa | scaffold194:190056..190114:+ | | | | IGR (Between DV3010043 and DV3010044) |
| Dvit-miR-x29 | scaffold2_214 | 155.1 | 311 | 266 | 0 | 45 | yes | uaauucuuauauugucgguuuug | aaacugacacuguaauuauuaaa | aaacugacacuguaauuauuaaauucuauauuauuuuaauucuuauauugucgguuuug | scaffold2:544098..544159:- | | | | 5UTR(of DV3002152) |
| Dvit-miR-x30 | scaffold208_12251 | 14.3 | 39 | 37 | 0 | 2 | yes | uauuugagauuauuaaaacugga | uaguuuuaguuuuucaucaaua | uaguuuuaguuuuucaucaauaaauugucaauaaaauuuauuugagauuauuaaaacugga | scaffold208:9877..9938:+ | | | | Iintron (of DV3018694) |
| Dvit-miR-x31 | scaffold210_12323 | 9.1 | 20 | 19 | 0 | 1 | yes | aauugacagaauaucguggaac | cccacaauguucugacaaauu | cccacaauguucugacaaauucacauucuuucuuuauggugaauugacagaauaucguggaac | scaffold210:57152..57215:+ | | | | IGR (Between DV3014482.2 and DV3014487) |
| Dvit-miR-x32 | scaffold216_12627 | 2901.4 | 5690 | 5106 | 0 | 584 | yes | aggcaagaugaggcaaagcug | agcuauggaucaucaggccacu | aggcaagaugaggcaaagcuguaguaauugcauugugcaaaguuuaaauuacagcuauggaucaucaggccacu | scaffold216:195981..196055:- | | | | IGR (Between DV3001277 and DV3001279) |
| Dvit-miR-x33 | scaffold222_12894 | 112.5 | 219 | 157 | 1 | 61 | yes | ucauuuucuuaguuggaucauu | ugauuuuacuaagaaaaugacu | ugauuuuacuaagaaaaugacugaucgauaacaauuuucaaccagauguuguugacuagucauuuucuuaguuggaucauu | scaffold222:65323..65404:+ | | | | IGR (Between DV3008972 and DV3008975) |
| Dvit-miR-x34 | scaffold223_12954 | 31.7 | 60 | 22 | 0 | 38 | yes | aagaugauggaaauuugagagc | ucucaaauuuccaucgucuuacg | ucucaaauuuccaucgucuuacgaccauacuugguguugaccauacuuaaagguccuuaagaugauggaaauuugagagc | scaffold223:135979..136059:- | | | | Intron (of DV30108520) |
| Dvit-miR-x35 | scaffold25_3191 | 109 | 218 | 201 | 0 | 17 | yes | uuuauaauuaucaguuguaug | guuaaucugguaauuauuugug | uuuauaauuaucaguuguaugaugugauuuucuaauugcgcuaauguuauuuaucguuaaucugguaauuauuugug | scaffold25:575027..575104:- | | | | Intron (of DV3004307) |
| Dvit-miR-x36 | scaffold252_14588 | 136.5 | 270 | 266 | 0 | 4 | yes | uugaacccugacaaaaugaaaa | uuuuguuuugggugggguuc | uugaacccugacaaaaugaaaauagcguuuuguugaaaaauacuugauuauucguuauuuuguuuuuguuuugggugggguuc | scaffold252:100095..100177:+ | | | | Intron (of DV3013673) |
| Dvit-miR-x37 | scaffold291_15962 | 136.6 | 268 | 244 | 0 | 24 | yes | cuguuucucuacuuacugaaca | uuuaguaagugaggaaacgg | uuuaguaagugaggaaacggauauuguucucuuuacuuuuuacaaucgaccuguuucucuacuuacugaaca | scaffold291:49815..49887:- | | | | IGR (Between DV3016942 and DV3025780.1) |
| Dvit-miR-x38 | scaffold299_16535 | 14593.5 | 28626 | 28500 | 5 | 121 | yes | uuaaaacuaggugggcgggcu | ccuguuucaucuaguuuugugu | ccuguuucaucuaguuuuguguuugauuguugaacauacuuaaaacuaggugggcgggcu | scaffold299:20498..20558:+ | Cluster6-2 | | | IGR (Between DV3005472 and DV3005476) |
| Dvit-miR-x39 | scaffold299_16542 | 17319.9 | 33966 | 30390 | 7 | 3569 | yes | auugguaacauguaacaucuuuag | aaagauguuuuuguugccuguau | auugguaacauguaacaucuuuaguaguaaauauauuaaucuaaagauguuuuuguugccuguau | scaffold299:21069..21134:+ | Cluster6-5 | | | IGR (Between DV3005472 and DV3005476) |
| Dvit-miR-x40 | scaffold299_16547 | 4.1 | 1182 | 1173 | 7 | 2 | yes | ugacuagaaguuauacuacaa | uaguauaauuuacuagauacauu | uaguauaauuuacuagauacauuaagugaaacaaauaucuaugugacuagaaguuauacuacaa | scaffold299:25709..25773:+ | Cluster6-8 | | | IGR (Between DV3005472 and DV3005476) |
| Dvit-miR-x41 | scaffold299_16557 | 5004.6 | 9819 | 9756 | 0 | 63 | yes | ucacuggugacaauuuaugaag | cugugaauuguuuccaguugau | cugugaauuguuuccaguugauacuaguauuuaauaguuaucacuggugacaauuuaugaag | scaffold299:27851..27913:+ | Cluster6-10 | | | IGR (Between DV3005472 and DV3005476) |
| Dvit-miR-x42 | scaffold302_16701 | 3.8 | 10 | 9 | 0 | 1 | yes | cgugcauuuuaacacauuggcug | cucuauguauuaaaaugcuugu | cgugcauuuuaacacauuggcuguuguuuauuuauacuccaaucacguuauauacucacucuauguauuaaaaugcuugu | scaffold302:59904..59984:- | | | | Intron (of DV3016241) |
| Dvit-miR-x43 | scaffold316_17173 | 5480.6 | 10750 | 10631 | 0 | 119 | yes | ucccaccgugauuuacugauug | auugguaaaucacguuggcaca | ucccaccgugauuuacugauuguaauaauuguucauauauucaaauugguaaaucacguuggcaca | scaffold316:246716..246782:- | | | | Intron (of DV3014120) |
| Dvit-miR-x44 | scaffold32_3617 | 158.9 | 321 | 180 | 0 | 141 | yes | cgcucuuuuuuuggucuucuaac | uagcugccuaaaaaagaguuau | cgcucuuuuuuuggucuucuaacauucaauuauuauaguuagcugccuaaaaaagaguuau | scaffold32:126829..126890:- | | | | IGR (Between DV3006152 and DV3006154.2) |
| Dvit-miR-x45 | scaffold328_17850 | 12.6 | 26 | 16 | 0 | 10 | yes | uaccgauucauaacaugacagg | cgucauuuuaugaaucgauagc | uaccgauucauaacaugacagggugauaguaucugucgucauuuuaugaaucgauagc | scaffold328:276118..276177:+ | | | | Intron (of DV3010320) |
| Dvit-miR-x46 | scaffold35_3781 | 12.7 | 31 | 29 | 0 | 2 | yes | aauuucguuaaaauaaaggga | uccuuuauucuaaugaaaguuggu | uccuuuauucuaaugaaaguugguaugaaaaacuuacgaauuucguuaaaauaaaggga | scaffold35:269494..269554:+ | | | | IGR (Between DV3003411 and DV3003415) |
| Dvit-miR-x47 | scaffold37_3973 | 3642.6 | 7143 | 3218 | 6 | 3919 | yes | agacgaggugguuguuacauuaa | uuaauguaauaaccacucgucu | uuaauguaauaaccacucgucuuuuggaucuguuguugaagacgagguguuguuacauuaa | scaffold37:281232..281294:+ | | | | Intron (of DV3005042) |
| Dvit-miR-x48 | scaffold398_21234 | 16.7 | 53 | 50 | 0 | 3 | yes | cauaaaucguuuuuuaagaaaug | uuucuuuuaaaugauuuguuaa | cauaaaucguuuuuuaagaaaugauguuuguaaaaaauauuucuuuuaaaugauuuguuaa | scaffold398:47857..47918:+ | | | | IGR (Between DV3025926.1 and DV3016862.2) |
| Dvit-miR-x49 | scaffold4_334 | 494.7 | 969 | 964 | 0 | 5 | yes | uuuguacaucagagguaaacu | uuuaccucagguguacgguaua | uuuguacaucagagguaaacuaaguuacauuuuuugaaaauuugucuuguaaaaguuuaccucagguguacgguaua | scaffold4:1000891..1000969:+ | | | | Intron (of DV3001120) |
| Dvit-miR-x50 | scaffold40_4175 | 115.3 | 223 | 149 | 0 | 74 | yes | uaagacuaaguguuaaucggacc | uucgauuaacacuuagucuuaug | uucgauuaacacuuagucuuauguaagacuaauaggaacuuauaagacuaaguguuaaucggacc | scaffold40:354930..354995:+ | | | | IGR (Between DV3003900 and DV3003901) |
| Dvit-miR-x51 | scaffold40_4221 | 0.4 | 69 | 49 | 0 | 20 | yes | aggacaguaauauucaaauuuga | ucacuuuuaaauauuacugcca | aggacaguaauauucaaauuugacguuuggggucacuuuuaaauauuacugcca | scaffold40:597130..597184:- | | | | Intron (of DV3025931.1) |
| Dvit-miR-x52 | scaffold465_23530 | 173.3 | 338 | 316 | 0 | 22 | yes | ucuagcuaggccuuauuacc | uaauaaggccuugcuaagcuu | uaauaaggccuugcuaagcuuguauuagauuuugcauaaagucuagcuaggccuuauuacc | scaffold465:60474..60535:- | | | | IGR (Between DV3015083 and DV3015085) |
| Dvit-miR-x53 | scaffold476_23777 | 16.1 | 32 | 21 | 1 | 10 | yes | caaagguguaaugauauuuggc | caaauucaauuacaccauugac | caaagguguaaugauauuuggcauauaauaauaauugucuagcgucaaauucaauuacaccauugac | scaffold476:63725..63792:- | | | | Intron (of DV3017856) |
| Dvit-miR-x54 | scaffold48_4598 | 234.5 | 466 | 465 | 0 | 1 | yes | aaauauuguuuuaguagggucu | agcccuaguaacacaauauauc | aaauauuguuuuaguagggucuuuuaauaacuaagcccuaguaacacaauauauc | scaffold48:271712..271767:+ | | | | Intron (of DV3001386) |
| Dvit-miR-x55 | scaffold515_24882 | 22147.6 | 43441 | 40590 | 1 | 2850 | yes | ugugauguguuugugagccacu | uggcccacaaccacagaacuuc | ugugauguguuugugagccacugugauauuauauaauaguuaguggcccacaaccacagaacuuc | scaffold515:101561..101626:+ | | | | IGR (Between DV3013911 and DV3013912) |
| Dvit-miR-x56 | scaffold57_5285 | 15766.4 | 30927 | 28396 | 25 | 2506 | yes | uaaagauguuuacuacucu | ggguagaauaaacgucuuuugc | ggguagaauaaacgucuuuugcuaguuuaagcuauuuuucaauauucugcuaaagauguuuacuacucu | scaffold57:345834..345903:- | Cluster9-7 | | | IGR (Between DV3005077.2 and DV3005080.2) |
| Dvit-miR-x57 | scaffold586_28079 | 7.6 | 17 | 16 | 0 | 1 | yes | uaaucaauuacuuuuauuagcu | uaauagguggauugauuaca | uaaucaauuacuuuuauuagcuacuuuagauaacgcaacaguguuaaaaagcacagcuaauagguggauugauuaca | scaffold586:35158..35235:- | | | | Exon (of DV3017763) |

**Table S11.** List of clock-related genes searched for in the genome of *D. vitifoliae*.

| Genes | Symbol | Prediction[1] | Scaffold number | *A. pisum* homolog(s) | Comments |
|---|---|---|---|---|---|
| **Input-related** | | | | | |
| *long wavelength opsin* | Dvit*LWO* | DV3019248 | 644 | ACYPI009332 | |
| *short wavelength opsin* | Dvit*SWO* | DV3010484 | 153 | ACYPI004442 ACYPI002544 | Duplicated in *A. pisum* |
| *Arthropsin* | Dvit*arthrops* | DV3009221 | 308 | ACYPI32968 | |
| *C-opsin* | Dvit*C-ops* | DV3010731 | 104 | ACYPI009397 | |
| *Rhodopsin 7* | Dvit*Rh7* | DV3008393 | 648 | ACYPI001006 ACYPI005074 | Duplicated in *A. pisum* |
| *cryptochrome 1* | Dvit*cry1* | DV3005203 | 141 | ACYPI005757 | |
| ***Core*** | | | | | |
| *period* | Dvit*per* | DV3009458 | 378 | ACYPI47669 | |
| *timeless* | Dvit*tim* | DV3010827 [2] | 270 | ACYPI36439 | Manually annotated (absent in the gene predictions set) |
| *Clock* | Dvit*Clk* | DV3008870 | 180 | ACYPI004812 | |
| *cycle* | Dvit*cyc* | DV3002036 | 95 | ACYPI004686 | |
| *PAR-domain protein 1* | Dvit*Pdp1* | DV3000657 | 27 | ACYPI004812 | |
| *vrille* | Dvit*vri* | DV3002370 | 1 | ACYPI008851 | |
| *cryptochrome 2* | Dvit*cry2* | DV3016418 | 579 | ACYPI006584 ACYPI004197 | Duplicated in *A. pisum* |
| **Output-related** | | | | | |
| *arylalkylamine N-acetyltransferase 1* | Dvit*aanat1* | DV3007886 | 29 | ACYPI002543 | *aanat1* and *aanat2* genes in tandem in the genome, as observed in *A. pisum*. |
| *arylalkylamine N-acetyltransferase 2* | Dvit*aanat2* | DV3007886 | 29 | ACYPI000655 | *aanat1* and *aanat2* genes in tandem in the genome, as observed in *A. pisum*. |
| *arylalkylamine N-acetyltransferase 3* | Dvit*aanat3* | | | ACYPI000856 | absent in *D. vitifoliae* |
| *arylalkylamine N-acetyltransferase 4* | Dvit*aanat4* | | | ACYPI060713 | absent in *D. vitifoliae* |
| *Prothoracicotropic hormone* | Dvit*Ptth* | DV3024948 [2] | 43 | ACYPI37989 | Manually annotated (absent in the gene predictions set) |
| **Ancillary and related genes** | | | | | |
| *Photolyase 6-4* | Dvit*Ph6-4* | DV3000677 | 27 | ACYPI003810 | |
| *timeless2/timeout* | Dvit*tim2/timeout* | DV3016946 | 291 | ACYPI001257 | |
| *clockwork orange* | Dvit*cwo* | DV3001859 | 12 | ACYPI24234 | |
| *doubletime/casein kinase* | Dvit*dbt/CKI* | DV3003852 | 168 | ACYPI008162 | |
| *supernumerary limbs* | Dvit*slmb* | DV3004609 | 15 | ACYPI006874 | |
| *casein kinase II α* | Dvit*CKIIα* | DV3013255 | 327 | ACYPI002006 | |
| *casein kinase II β* | Dvit*CKIIβ* | DV3005119 | 56 | ACYPI000089 | |
| *twins* | Dvit*tws* | DV3002660 | 53 | ACYPI009741 | |
| *widerborst* | Dvit*wdb* | DV3002950 | 50 | ACYPI000666 | |
| *shaggy/glycogen synthase kinase 3* | Dvit*sgg/GSK3* | DV3017481 | 678 | ACYPI009257 | Absent both in *A. pisum* and *D. vitifoliae* |

[1] Assigned gene name in the *D. vitifoliae* database (http://bipaa.genouest.org/sp/daktulosphaira_vitifoliae/)

[2] Gene initially not annotated (no gene model in automatic predictions). This gene was identified manually.

**Table S12.** List of OR and GR annotated genes. Part1 (OR):

| Name | Symbol | Scaffold number | Complete? | Notes |
|---|---|---|---|---|
| Olfactory receptor co-receptor | ORco | 54 | complete | |
| Olfactory receptor 1 allele A | OR1_A | 109 | complete | |
| Olfactory receptor 1 allele B | OR1_B | 4219 | partial | lacks Nter |
| Olfactory receptor 2 | OR2 | 425 | complete | |
| Olfactory receptor 3 | OR3 | 523 | partial | lacks Nter |
| Olfactory receptor 4 | OR4 | 403 | partial | lacks Nter |
| Olfactory receptor 5 | OR5 | 244 | partial | Model corrected, lacks N-ter. |
| Olfactory receptor 6 | OR6 | 428 | complete | |
| Olfactory receptor 7 | OR7 | 259 | partial | lacks N-ter, beginning of scaffold |
| Olfactory receptor 8 | OR8 | 259 | complete | |
| Olfactory receptor 9 | OR9 | 259 | complete | |
| Olfactory receptor 10 | OR10 | 259 | partial | one exon/intron junction is not good but N in sequence |
| Olfactory receptor 11 | OR11 | 259 | partial | N-ter only, end of scaffold |
| Olfactory receptor 12 | OR12 | 380 | complete | |
| Olfactory receptor 13 allele A | OR13_A | 179 | complete | |
| Olfactory receptor 13 allele B | OR13_B | 8233 | partial | small fragment |
| Olfactory receptor 14 | OR14 | 289 | complete | |
| Olfactory receptor 15 | OR15 | 229 | partial | C-ter only |
| Olfactory receptor 16 | OR16 | 229 | complete | |
| Olfactory receptor 17 | OR17 | 15 | complete | abnormal intron-exon boundary |
| Olfactory receptor 18 | OR18 | 15 | complete | STOP codon in 1st exon |
| Olfactory receptor 19 | OR19 | 15 | complete | abnormal intron-exon boundary |
| Olfactory receptor 20 | OR20 | 15 | partial | lack C-ter |
| Olfactory receptor 21 | OR21 | 15 | complete | |
| Olfactory receptor 22 | OR22 | 15 | partial | readthrough stop codon, pseudogene?, lacks C-ter |
| Olfactory receptor 23 | OR23 | 186 | complete | |
| Olfactory receptor 24 | OR24 | 186 | partial | 1 exon lacking |
| Olfactory receptor 25 | OR25 | 186 | partial | 1 exon lacking |
| Olfactory receptor 26 | OR26 | 186 | complete | |
| Olfactory receptor 27 | OR27 | 186 | partial | 1 exon lacking |
| Olfactory receptor 28 | OR28 | 186 | partial | |
| Olfactory receptor 29 | OR29 | 711 | complete | |
| Olfactory receptor 30 | OR30 | 711 | partial | 1 exon lacking |
| Olfactory receptor 31 | OR31 | 711 | partial | lacks Nter |
| Olfactory receptor 32 | OR32 | 682 | complete | |
| Olfactory receptor 33 | OR33 | 66 | complete | |
| Olfactory receptor 34 | OR34 | 66 | partial | N-ter only, no clue for C-ter |
| Olfactory receptor 35 | OR35 | 973 | partial | c-ter missing |
| Olfactory receptor 36 | OR36 | 237 | complete | |
| Olfactory receptor 37 | OR37 | 679 | complete | |
| Olfactory receptor 38 | OR38 | 293 | complete | |
| Olfactory receptor 39 | OR39 | 293 | complete | |
| Olfactory receptor 40 | OR40 | 1209 | complete | |
| Olfactory receptor 41 | OR41 | 1209 | complete | |
| Olfactory receptor 42 | OR42 | 861 | complete | |
| Olfactory receptor 43 | OR43 | 460 | complete | |
| Olfactory receptor 44 | OR44 | 11 | complete | |
| Olfactory receptor 45 | OR45 | 39 | complete | readthrough stop codon |
| Olfactory receptor 46 | OR46 | 183 | complete | |
| Olfactory receptor 47 | OR47 | 933 | complete | |
| Olfactory receptor 48 | OR48 | 614 | complete | |
| Olfactory receptor 49 | OR49 | 761 | complete | |
| Olfactory receptor 50 | OR50 | 86 | complete | |
| Olfactory receptor 51 | OR51 | 16 | partial | N-ter missing, |
| Olfactory receptor 52 | OR52 | 168 | partial | |
| Olfactory receptor 53 | OR53 | 1845 | partial | N-ter only |
| Olfactory receptor 54 | OR54 | 328 | partial | N-ter only, putative pseudo-gene ? (STOP codon) |
| Olfactory receptor 55 | OR55 | 3666 | partial | |
| Olfactory receptor 56 | OR56 | 507 | partial | C-ter only |
| Olfactory receptor 57 | OR57 | 7347 | partial | C-ter only |
| Olfactory receptor 58 | OR58 | 740 | partial | small fragment |
| Olfactory receptor 59 | OR59 | 7822 | partial | small fragment |
| Olfactory receptor 60 | OR60 | 800 | partial | C-ter only, putative pseudo-gene ? (STOP codon) |

**Table S12.** Legend in the preceding page. Part2 (GR).

| Name | Symbol | Scaffold number | Complete? | Notes |
|---|---|---|---|---|
| Gustatory receptor 1 | GR1 | scaffold13 | complete | |
| Gustatory receptor 2 | GR2 | scaffold596 | partial | lack terminal exon in C-ter |
| Gustatory receptor 3 | GR3 | scaffold63 | partial | |
| Gustatory receptor 4 | GR4 | scaffold41 | partial | |
| Gustatory receptor 5 | GR5 | scaffold344 | complete | |
| Gustatory receptor 6 | GR6 | scaffold344 | complete | |
| Gustatory receptor 7 | GR7 | scaffold106 | partial | |
| Gustatory receptor 8 | GR8 | scaffold106 | partial | very end of C-ter is missing |
| Gustatory receptor 9 | GR9 | scaffold42 | complete | |
| Gustatory receptor 10 | GR10 | scaffold308 | complete | |
| Gustatory receptor 11 | GR11 | scaffold50 | complete | |
| Gustatory receptor 12 | GR12 | scaffold939 | partial | |
| Gustatory receptor 13 | GR13 | scaffold939 | partial | |
| Gustatory receptor 14 | GR14 | scaffold506 | partial | N-ter is missing |
| Gustatory receptor 15 | GR15 | scaffold1390 | partial | |
| Gustatory receptor 16 | GR16 | scaffold54 | partial | |
| Gustatory receptor 17 | GR17 | scaffold39 | partial | |
| Gustatory receptor 18 | GR18 | scaffold144 | partial | predicted partial gene model |

**Table S13, Part1.** Number of OBP (A), CSP (B) and NPC2 (C) members identified in *D. vitifoliae* and *A. pisum* (known orthologous relationships are specified). Th gene DvitCSP11 was found in the grape phylloxera genome by using exhaustive searches performed by the BITACORA pipeline ( http://www.ub.edu/softevol/bitacora/) [36]. In gene names, a and b refer to paralogs.

**A)**

| | *D. vitifoliae* | | *A. pisum* | |
|---|---|---|---|---|
| **Orthogroup** | **Number of genes** | **Gene Name** | **Number of genes** | **Gene Name** |
| **OBP2** | 1 | DvitiObp2 | 1 | ApisObp2 |
| **OBP3** | 1 | DvitiObp3 | 3 | ApisObp3, ApisObp11, ApisObp12 |
| **OBP4** | 1 | DvitiObp4 | 1 | ApisObp4 |
| **OBP5** | 1 | DvitiObp5 | 1 | ApisObp5 |
| **OBP6** | 2 | DvitiObp6a, DvitiObp6b | 1 | ApisObp6 |
| **OBP7** | 0 | | 1 | ApisObp7 |
| **OBP8** | 2 | DvitiObp8a, DvitiObp8b[1,2] | 1 | ApisObp8 |
| **OBP9** | 1 | DvitiObp9 | 1 | ApisObp9 |
| **OBP10** | 1 | DvitiObp10 | 1 | ApisObp10 |
| **OBP13** | 1 | DvitiObp13 | 1 | ApisObp13 |
| **OBP14** | 1 | DvitiObp14 | 1 | ApisObp14 |
| **Not assigned** | 1 | DvitiObpU[1,2,3] | 0 | |
| **Total** | **13** | | 13 | |

[1] partial genes, [2] not included in the phylogenetic analysis, [3] dubious model

**B)**

| | *D. vitifoliae* | | *A. pisum* | |
|---|---|---|---|---|
| **Orthogroup** | **Number of genes** | **Gene Name** | **Number of genes** | **Gene Name** |
| **CSP1** | 3 | DvitiCsp1a, DvitiCsp1b[1,2], DvitiCsp1c | 2 | ApisCsp1 |
| **CSP2-3** | 1 | DvitiCsp2 | 3 | ApisCsp2, ApisCsp3, ApisCsp3_2 |
| **CSP4** | 1 | DvitiCsp4 | 1 | ApisCsp4 |
| **CSP5** | 1 | DvitiCsp5 | 1 | ApisCsp5 |
| **CSP6** | 1 | DvitiCsp6 | 1 | ApisCsp6 |
| **CSP7** | 1 | DvitiCsp7 | 1 | ApisCsp7 |
| **CSP8** | 1 | DvitiCsp8 | 1 | ApisCsp8 |
| **CSP9** | 0 | | 1 | ApisCsp9 |
| **CSP10** | 2 | DvitiCsp10a, Csp10b | 1 | ApisCsp10 |
| **CSP11** | 1 | DvitiCsp11 [3] | 1 | ApisCsp11 |
| **Total** | **12** | | 13 | |

[1] partial gene, [2] not included in the phylogenetic analysis, [3] named DviCSP9 in the study of [37].

**Table S13, Part2.** Legend in the preceding page. Since homologous relationships among NPC2 could not be unambigously determined, we used numbered suffixes to name the different copies.

**C)**

| | *D. vitifoliae* | | *A. pisum* | |
|---|---|---|---|---|
| **Orthogroup** | **Number of genes** | **Gene Name** | **Number of genes** | **Gene Name** |
| **NPC2_1** | 1 | DvitiNpc2_1 | 1 | ApisNpc2_pre |
| | 1 | DvitiNpc2_2 | | |
| | 1 | DvitiNpc2_3 | | |
| | 1 | DvitiNpc2_4 | | |
| | 1 | DvitiNpc2_5 | | |
| | 1 | DvitiNpc2_6 | | |
| | 1 | DvitiNpc2_7 | | |
| | 1 | DvitiNpc2_8 | | |
| | | | 1 | ApisNpc2_1 |
| | | | 1 | ApisNpc2_3 |
| | | | 1 | ApisNpc2_4 |
| | | | 1 | ApisNpc2_5 |
| | | | 1 | ApisNpc2_6 |
| | | | 1 | ApisNpc2_7 |
| **Total** | **8** | | **7** | |

**Table S14.** List of genes for Cytochromes P450, UDP-glycosyl transferases, Glutathione S-transferases, Carboxylesterases and ABC transporters annotated in the genome of grape phylloxera. Scaffold= scaffold number of the gene; position or transcript name= Position of the gene on the scaffold or transcript name given by the automatic annotation; Strand= Strand on which the gene has been annotated; size (aa)= Number of amino-acids of the corresponding protein; detox family= Cytochrome P450 (CYP), UDP-glucosyl transferase (UGT), Glutathione S-transferase (GST), Carboxylesterase (CCE) and ABC transporter; name_ID= Unique ID given by the annotator for CYP and display on the genome browser; Classification=Clan 2, 3 or 4 or mitochondrial clan for CYP; Glutathione S-transferase delta, omega, sigma, or theta for GST; family number for UGT; Pheromone/hormone processing, Neuro/developmental, Detoxification/dietary class for CCE; ABC A, B, C, D, E, F ,G, and H for ABC transporter. Name= Unique ID given by the annotators for UGTs, GSTs, CCEs and ABC transporters and by D. Nelson for CYP genes. Comments= Contain additional information on gene structures or transcripts names.

**Table S14, Part1.** See above the Legend.

| scaffold number | position or transcript name | strand | size (aa) | Number of exons | detox family | name_ID | classification | name |
|---|---|---|---|---|---|---|---|---|
| 89 | DV3004986-RA-00001 | minus | 238 | 6 | GST | | Glutathione S-transferase omega | GSTo1 |
| 140 | DV3010592-RA-00001 | minus | 244 | 6 | GST | | Glutathione S-transferase omega | GSTo2 |
| 421 | DV3016774-RA | plus | 203 | 4 | GST | | Glutathione S-transferase sigma | GSTs1 |
| 421 | DV3016773-RA | plus | 205 | 4 | GST | | Glutathione S-transferase sigma | GSTs2 |
| 44 | DV3004805-RA-00001 | minus | 201 | 4 | GST | | Glutathione S-transferase sigma | GSTs3 |
| 77 | DV3003239-RA-00001 | plus | 214 | 5 | GST | | Glutathione S-transferase delta | GSTd1 |
| 77 | DV3003238-RA-00001 | plus | 217 | 5 | GST | | Glutathione S-transferase delta | GSTd2 |
| 416 | DV3008706-RA-00001 | minus | 233 | 5 | GST | | Glutathione S-transferase theta | GSTt1 |
| 283 | DV3007510-RA-00001 | minus | 149 | 3 | GST | | Glutathione S-transferase microsomal | MGST1 |
| 283 | DV3007514-RA-00001 | minus | 161 | 3 | GST | | Glutathione S-transferase microsomal | MGST2 |
| 35 | DV3003399-RA-00001 | minus | 158 | 3 | GST | | Glutathione S-transferase microsomal | MGST3 |
| 6 | DV3000285-RA-00001 | plus | 529 | 6 | UGT | | 350C1 | UGT_1 |
| 19 | DV3006825-RA-00001 | minus | 509 | 5 | UGT | | 343B | UGT_2 |
| 30 | DV3002826-RA-00001 | plus | 514 | 4 | UGT | | 329A | UGT_3 |
| 61 | DV3002241-RA-00001 | minus | 531 | 5 | UGT | | 350C3 | UGT_4 |
| 61 | DV3002242-RA-00001 | minus | 530 | 6 | UGT | | 350C2 | UGT_5 |
| 101 | DV3005994-RA-00001 | minus | 523 | 4 | UGT | | 344N1 | UGT_6 |
| 137 | DV3003766-RAa-00001 | plus | 489 | 4 | UGT | | 329B | UGT_7 |
| 198 | DV3008018-RA-00001 | minus | 513 | 4 | UGT | | 344N5 | UGT_8 |
| 294 | DV3017396-RA-00001 | minus | 517 | 6 | UGT | | 330A | UGT_9 |
| 344 | DV301262+B258-RA-00001 | plus | 508 | 4 | UGT | | 348A1 | UGT_10 |
| 354 | DV3007831-RA-00001 | plus | 423 | 2 | UGT | | 349A1 | UGT_11 |
| 398 | DV3016865-RA-00001 | plus | 509 | 4 | UGT | | 339A | UGT_12 |
| 424 | DV3008618-RA-00001 | minus | 517 | 5 | UGT | | 349A3 | UGT_13 |
| 424 | DV3008619-RAaa-00001 | minus | 512 | 5 | UGT | | 349A4 | UGT_14 |
| 424 | DV3008619-RAa-00002 | minus | 510 | 5 | UGT | | 349A5 | UGT_15 |
| 424 | DV3008619-RA-00002 | minus | 515 | 5 | UGT | | 342A | UGT_16 |
| 424 | DV3008628-RA-00001 | plus | 517 | 5 | UGT | | 349A2 | UGT_17 |
| 431 | blast_result_XP_003245050_scaffold431_-00001 | plus | 502 | 4 | UGT | | 348A2 | UGT_18 |
| 443 | DV3014656-RAa-00001 | minus | 517 | 5 | UGT | | 343C4 | UGT_19 |
| 510 | DV3009069-RA-00001 | plus | 525 | 8 | UGT | | 341A | UGT_20 |
| 519 | DV3019637-RA-00001 | plus | 244 | 4 | UGT | | 344M1 | UGT_21 |
| 565 | DV3015063-RA-00001 | minus | 547 | 5 | UGT | | 351A | UGT_22 |
| 572 | DV3016285-RAaaa-00001 | minus | 525 | 4 | UGT | | 344N2 | UGT_23 |
| 572 | DV3016285-RAaa-00002 | minus | 507 | 6 | UGT | | 344N3 | UGT_24 |
| 572 | DV3016285-RAa-00002 | minus | 512 | 4 | UGT | | 344N9 | UGT_25 |
| 572 | DV3016285-RA-00001 | minus | 538 | 5 | UGT | | 344N10 | UGT_26 |
| 572 | DV3016286-RA-00001 | minus | 489 | 5 | UGT | | 344N7 | UGT_27 |
| 572 | DV3016290-RAa-00001 | minus | 505 | 4 | UGT | | 344N8 | UGT_28 |
| 572 | DV3016290-RA-00001 | minus | 520 | 4 | UGT | | 344N6 | UGT_29 |
| 572 | DV3016291-RAa-00001 | minus | 278 | 1 | UGT | | 344N4 | UGT_30 |
| 572 | DV3016291-RA-00001 | minus | 472 | 4 | UGT | | 344M2 | UGT_31 |
| 766 | DV3015648-RA-00001 | plus | 517 | 5 | UGT | | 343C1 | UGT_32 |
| 766 | DV3015649-RA-00001 | plus | 517 | 5 | UGT | | 343C3 | UGT_33 |
| 1238 | DV3021594-RA-00001 | plus | 520 | 5 | UGT | | 343C2 | UGT_34 |
| 4315 | blast_result_XP_016661510_scaffold4315_-00001 | minus | 231 | 1 | UGT | | 344F | UGT_35 |
| 212 | 11667-20901 | minus | *562* | 7 | CCE | | Pheromone/hormone processing | CCE-1 |
| 212 | 31598-38026 | minus | *558* | 7 | CCE | | Pheromone/hormone processing | CCE-2 |
| 212 | 46944-50145 | minus | 560 | 7 | CCE | | Pheromone/hormone processing | CCE-3 |
| 212 | 60220-74779 | minus | 576 | 7 | CCE | | Pheromone/hormone processing | CCE-4 |
| 114 | 260409-273625 | minus | 603 | 9 | CCE | | Pheromone/hormone processing | CCE-5 |
| 114 | 220014-226309 | minus | 579 | 8 | CCE | | Pheromone/hormone processing | CCE-6 |
| 114 | 278979-295821 | minus | 604 | 11 | CCE | | Pheromone/hormone processing | CCE-7 |
| 1086 | 27800-31467 | plus | 687 | 6 | CCE | | Neuro/developmental (clade J) | CCE-8 |
| 118 | 274871-303671 | plus | 654 | 11 | CCE | | Neuro/developmental (clade J) | CCE-9 |
| 27 | 1062697-1067673 | minus | 559 | 8 | CCE | | Pheromone/hormone processing | CCE-10 |
| 195 | 144240-148535 | minus | 584 | 10 | CCE | | Pheromone/hormone processing | CCE-11 |
| 195 | 150860-157187 | plus | 572 | 8 | CCE | | Pheromone/hormone processing | CCE-12 |
| 13 | 333949-356626 | minus | 757 | 9 | CCE | | Neuro/developmental (clade L) | CCE-13 |
| 20 | 391435-589634 | minus | 968 | 10 | CCE | | Neuro/developmental (clade L) | CCE-14 |
| 134 | 191123-197284 | minus | 828 | 13 | CCE | | Neuro/developmental (clade K) | CCE-15 |
| 146 | 545984-560565 | plus | 755 | 12 | CCE | | Neuro/developmental (clade H) | CCE-16 |
| 191 | 59224-78875 | minus | 553 | 11 | CCE | | Detoxification/dietary class | CCE-17 |
| 191 | 80077-88986 | minus | 568 | 11 | CCE | | Detoxification/dietary class | CCE-18 |
| 334 | 35419-56136 | plus | 1153 | 11 | CCE | | Neuro/developmental (clade L) | CCE-19 |
| 775 | 11760-34482 | plus | 649 | 3 | CCE | | Neuro/developmental (unknown) | CCE-20 |

## **Table S14, Part2.** See above for Legend.

| scaffold number | position or transcript name | strand | size (aa) | Number of exons | detox family | classification | name | comments |
|---|---|---|---|---|---|---|---|---|
| 447 | 35973-26849 | minus | 1633 | 24 | ABC transporter | ABCA | ABCA1 | DV3016087-RA |
| 391 | 15026-0 | minus | 1744 | 21 | ABC transporter | ABCA | ABCA2 | DV3013382-RA |
| 2301 | 10497-6026 | minus | 820 | 12 | ABC transporter | ABCA | ABCA3partial | DV3022439-RA |
| 502 | 129486-160967 | plus | 1556 | 19 | ABC transporter | ABCA | ABCA4 | DV3011141-RA |
| 249 | 32974-50780 | plus | 1307 | 27 | ABC transporter | ABCB | ABCB1 | DV3010127-RA |
| 126 | 191664-172277 | minus | 1432 | 27 | ABC transporter | ABCB | ABCB2 | DV3007299-RA |
| 3 | 586320-591820 | plus | 670 | 14 | ABC transporter | ABCB | ABCB3 | DV3008235-RA |
| 330 | 11089-6571 | minus | 644 | 10 | ABC transporter | ABCB | ABCB4 | DV3003320-RA |
| 168 | 730217-737766 | plus | 1048 | 12 | ABC transporter | ABCB | ABCB5 | DV3003862-RA |
| 288 | 187905-174604 | minus | 839 | 14 | ABC transporter | ABCB | ABCB6 | DV3014308-RA |
| 27 | 639228-605373 | minus | 1514 | 29 | ABC transporter | ABCC | ABCC1 | DV3000687-RA |
| 82 | 365312-326212 | minus | 1344 | 27 | ABC transporter | ABCC | ABCC2 | DV3006329-RA |
| 82 | 286282-256234 | minus | 1361 | 25 | ABC transporter | ABCC | ABCC3 | DV3006327-RA<br>DV3006328-RA |
| 159 | 25553-1974 | minus | 1493 | 20 | ABC transporter | ABCC | ABCC4 | DV3008821-RA<br>DV3008819-RA |
| 8 | 1078397-1070560 | minus | 1657 | 26 | ABC transporter | ABCC | ABCC5 | DV3000399-RA |
| 106 | 284926-273707 | minus | 664 | 11 | ABC transporter | ABCD | ABCD1 | DV3007820-RA |
| 294 | 20415-10029 | minus | 721 | 11 | ABC transporter | ABCD | ABCD2 | DV3017393-RA |
| 1331 | 19105-17036 | minus | 333 | 7 | ABC transporter | ABCD | ABCD3partial | DV3022459-RA |
| 93 | 29750-33773 | plus | 608 | 7 | ABC transporter | ABCE | ABCE1 | DV3002710-RA |
| 500 | 40965-55584 | plus | 870 | 17 | ABC transporter | ABCF | ABCF1 | DV3014627-RA |
| 188 | 52100-56606 | plus | 618 | 13 | ABC transporter | ABCF | ABCF2 | DV3007322-RA |
| 144 | 121311-127307 | plus | 716 | 13 | ABC transporter | ABCF | ABCF3 | DV3011964-RA |
| 88 | 798381-788310 | minus | 695 | 13 | ABC transporter | ABCG | ABCG1 | DV3001032-RA |
| 329 | 172411-185492 | plus | 619 | 12 | ABC transporter | ABCG | ABCG2 | DV3014885-RA |
| 40 | 189294-204850 | plus | 615 | 11 | ABC transporter | ABCG | ABCG3 | DV3003887-RA |
| 88 | 759119-752052 | minus | 639 | 10 | ABC transporter | ABCG | ABCG4 | DV3001031-RA |
| 88 | 727736-743252 | plus | 610 | 11 | ABC transporter | ABCG | ABCG5 | DV3001029-RA |
| 200 | 30922-24199 | minus | 570 | 10 | ABC transporter | ABCG | ABCG6 | DV3005271-RA |
| 215 | 164774-181479 | plus | 693 | 11 | ABC transporter | ABCG | ABCG7 | DV3008090-RA |
| 763 | 83551-73607 | minus | 617 | 9 | ABC transporter | ABCG | ABCG8 | DV3019657-RA |
| 40 | 228657-245631 | plus | 915 | 11 | ABC transporter | ABCG | ABCG9 | DV3003890-RA |
| 40 | 248917-259465 | plus | 599 | 12 | ABC transporter | ABCG | ABCG10 | DV3003891-RA |
| 101 | 157821-150312 | minus | 564 | 11 | ABC transporter | ABCG | ABCG11 | DV3005975-RA |
| 213 | 592317-579236 | minus | 657 | 13 | ABC transporter | ABCG | ABCG12 | DV3007427-RA |
| 213 | 577596-569977 | minus | 594 | 12 | ABC transporter | ABCG | ABCG13 | DV3007427-RA |
| 213 | 562729-556488 | minus | 601 | 12 | ABC transporter | ABCG | ABCG14 | DV3007426-RA |
| 427 | 317312-310519 | minus | 595 | 13 | ABC transporter | ABCG | ABCG15 | DV3013094-RA |
| 289 | 299381-308640 | plus | 677 | 14 | ABC transporter | ABCG | ABCG16 | DV3013706-RA |
| 101 | 273872-264989 | minus | 649 | 13 | ABC transporter | ABCG | ABCG17 white | DV3005986-RA |
| 20 | 729099-737489 | plus | 623 | 14 | ABC transporter | ABCG | ABCG18 scarlet | DV3007978-RA |
| 358 | 142642-148216 | plus | 613 | 15 | ABC transporter | ABCG | ABCG19 scarlet-like | DV3015962-RA |
| 206 | 127698-115445 | minus | 688 | 18 | ABC transporter | ABCG | ABCG20 scarlet-like | DV3013197-RA |
| 31 | 347058-356851 | plus | 638 | 9 | ABC transporter | ABCG | ABCG21 | DV3006727-RA |
| 31 | 335858-331537 | minus | 915 | 14 | ABC transporter | ABCG | ABCG22 | DV3006726-RA |
| 607 | 18050-4589 | minus | 760 | 14 | ABC transporter | ABCH | ABCH1 | DV3019319-RA |
| 231 | 63052-40478 | minus | 778 | 16 | ABC transporter | ABCH | ABCH2 | DV3018892-RA |
| 1067 | 16890-29348 | plus | 443 | 10 | ABC transporter | ABCH | ABCH3partial | DV3022536-RA |
| 1882 | 12035-5705 | minus | 406 | 9 | ABC transporter | ABCH | ABCH4partial | DV3022675-RA |
| 581 | 69870-61307 | minus | 692 | 14 | ABC transporter | ABCH | ABCH5 | DV3015611-RA |
| 97 | 283470-265140 | minus | 711 | 15 | ABC transporter | ABCH | ABCH6 | DV3008435-RA |
| 184 | 285728-267463 | minus | 700 | 16 | ABC transporter | ABCH | ABCH7 | DV3003665-RA |
| 184 | 338036-326250 | minus | 645 | 15 | ABC transporter | ABCH | ABCH8 | DV3003667-RA<br>DV3003668-RA<br>DV3003669-RA |
| 246 | 89338-96515 | plus | 681 | 15 | ABC transporter | ABCH | ABCH9 | DV3013023-RA |
| 454 | 192106-196822 | plus | 541 | 11 | ABC transporter | ABCH | ABCH10 | DV3013584-RA |
| 36 | 300462-281066 | minus | 676 | 15 | ABC transporter | ABCH | ABCH11 | DV3002992-RA |
| 36 | 317234-303419 | minus | 675 | 15 | ABC transporter | ABCH | ABCH12 | DV3002993-RA |
| 36 | 279955-272244 | minus | 687 | 15 | ABC transporter | ABCH | ABCH13 | DV3002991-RA |
| 994 | 32446-12015 | minus | 678 | 15 | ABC transporter | ABCH | ABCH14 | DV3021404-RA |
| 489 | 4240-37038 | plus | 528 | 11 | ABC transporter | ABCH | ABCH15 | DV3020532-RA |
| 129 | 456698-481297 | plus | 832 | 17 | ABC transporter | ABCH | ABCH16 | DV3004573-RA |
| 129 | 484158-504969 | plus | 675 | 15 | ABC transporter | ABCH | ABCH17 | DV3004574-RA |
| 129 | 514068-538944 | plus | 677 | 15 | ABC transporter | ABCH | ABCH18 | DV3004576-RA |
| 129 | 547700-560694 | plus | 677 | 15 | ABC transporter | ABCH | ABCH19 | DV3004577-RA |
| 36 | 336129-324463 | minus | 678 | 15 | ABC transporter | ABCH | ABCH20 | DV3002994-RA |
| 184 | 301929-295340 | minus | 680 | 15 | ABC transporter | ABCH | ABCH21 | DV3003666-RA |
| 184 | 312226-306759 | minus | 679 | 15 | ABC transporter | ABCH | ABCH22 | DV3003666-RA |
| 184 | 324319-315890 | minus | 691 | 15 | ABC transporter | ABCH | ABCH23 | DV3003666-RA |

**Table S14, Part3.** See above for Legend.

| scaffold number | position or transcript name | strand | size (aa) | Number of exons | detox family | name_ID | classification | name |
|---|---|---|---|---|---|---|---|---|
| 1012 | 20126-42066 | minus | 466 | 8 | P450 | CYP041 | CLAN4 | CYP4LS2 |
| 1034 | 9378-19878 | minus | 454 | 7 PARTIAL | P450 | CYP044 | CLAN4 | CYP4CH4 |
| 107 | 293519-310023 | plus | 533 | 11 | P450 | CYP052 | CLAN4 | CYP380C36 |
| 109 | 329252-335722 | plus | 499 | 8 | P450 | CYP017 | CLAN2 | CYP15A1 |
| 11 | 255781-279372 | plus | 520 | 12 | P450 | CYP078 | CLAN4 | CYP380A1 |
| 11 | 208753-214325 | plus | 528 | 13 | P450 | CYP077 | CLAN4 | CYP380B1 |
| 1161 | 24968-25683 | plus | 109 | 2 PARTIAL | P450 | CYP051 | CLAN4 | CYP4CH4-fragment1 |
| 1233 | 5936-14055 | minus | 455 | 11 | P450 | CYP060 | CLAN4 | CYP380C25 |
| 1376 | 15259-16173 | minus | 233 | 3 PARTIAL | P450 | CYP073 | CLAN4 | CYP4CH-fragment1 |
| 146 | 427199-432200 | minus | 474 | 7 PARTIAL | P450 | CYP081 | CLANM | CYP302A1 |
| 156 | 377098-383366 | plus | 512 | 4 | P450 | CYP036 | CLAN3 | CYP6CY25 |
| 156 | 384366-387202 | plus | 513 | 5 | P450 | CYP035 | CLAN3 | CYP6CZ2 |
| 156 | 392333-392932 | plus | 297 | 2 PARTIAL | P450 | CYP088 | CLAN3 | CYP6CZ-fragment2 |
| 156 | 373687-375534 | plus | 515 | 5 | P450 | CYP037 | CLAN3 | CYP6NZ1 |
| 1566 | 12225-13139 | plus | 304 | 1 PARTIAL | P450 | CYP089 | CLAN3 | CYP6CY28-fragment |
| 1566 | 2887-9927 | plus | 483 | 5 | P450 | CYP033 | CLAN3 | CYP6PC2 |
| 158 | 232454-247451 | minus | 536 | 7 | P450 | CYP031 | CLAN3 | CYP6DD2 |
| 16 | 586876-598356 | minus | 512 | 11 | P450 | CYP032 | CLAN4 | CYP4CJ9 |
| 168 | 65191-85724 | minus | 564 | 13 | P450 | CYP054 | CLAN4 | CYP380C20 |
| 168 | 93228-115677 | minus | 537 | 12 | P450 | CYP055 | CLAN4 | CYP380C21 |
| 172 | 292652-296571 | minus | 500 | 7 | P450 | CYP034 | CLAN2 | CYP303A1 |
| 1808 | 5015-5602 | minus | 170 | 2 PARTIAL | P450 | CYP050 | CLAN4 | CYP4CH4-fragment2 |
| 183 | 286-1245 | plus | 234 | 5 PARTIAL | P450 | CYP053 | CLAN4 | CYP380C-fragment3 |
| 184 | 99346-103193 | plus | 499 | 5 | P450 | CYP038 | CLAN3 | CYP6CZ3 |
| 184 | 96231-97503 | plus | 296 | 2 PARTIAL | P450 | CYP039 | CLAN3 | CYP6CZ3-fragment |
| 184 | 28545-35027 | plus | 507 | 5 | P450 | CYP040 | CLAN3 | CYP6CZ4 |
| 184 | 367-5588 | plus | 477 | 10 | P450 | CYP004 | CLAN3 | CYP6CZ-fragment1 |
| 188 | 183722-196207 | minus | 524 | 7 PARTIAL | P450 | CYP049 | CLAN4 | CYP4LM2 |
| 19 | 255459-265708 | minus | 495 | 7 | P450 | CYP075 | CLAN4 | CYP4LS1 |
| 190 | 108980-118256 | plus | 444 | 8 PARTIAL | P450 | CYP048 | CLAN4 | CYP4CK1 |
| 204 | 367140-392225 | plus | 458 | 5 PARTIAL | P450 | CYP082 | CLAN3 | CYP3323B1 |
| 214 | 189191 .. 198508 | plus | 517 | 7 | P450 | CYP007 | CLAN4 | CYP4LP1 |
| 218 | 142145-148270 | plus | 514 | 7 | P450 | CYP071 | CLAN4 | CYP4LM1 |
| 218 | 149202-171616 | plus | 517 | 7 | P450 | CYP072 | CLAN4 | CYP4LN1 |
| 2347 | 497-2152 | plus | 250 | 4 PARTIAL | P450 | CYP080 | CLANM | CYP315A1 |
| 26 | 22157-24425 | minus | 512 | 4 | P450 | CYP020 | CLAN3 | CYP6CY27 |
| 26 | 2450-5282 | minus | 508 | 4 | P450 | CYP019 | CLAN3 | CYP6CY29 |
| 26 | 15981-19024 | minus | 498 | 5 | P450 | CYP021 | CLAN3 | CYP6CY30 |
| 26 | 540670-544976 | minus | 498 | 4 | P450 | CYP023 | CLAN3 | CYP6PD1 |
| 26 | 617688-618155 | minus | 118 | 2 PARTIAL | P450 | CYP026 | CLAN3 | CYP6PD-fragment1 |
| 26 | 673887-673976 | minus | 74 | 1 PARTIAL | P450 | CYP024 | CLAN3 | CYP6PD-fragment2 |
| 26 | 695303-695530 | minus | 76 | 1 PARTIAL | P450 | CYP025 | CLAN3 | CYP6PD-fragment3 |
| 26 | 713826-718215 | minus | 324 | 5 PARTIAL | P450 | CYP022 | CLAN3 | CYP6PD-fragment4 |
| 28 | 1045765-1056041 | plus | 524 | 3 | P450 | CYP027 | CLAN2 | CYP307A2 |
| 28 | 905594-907642 | minus | 510 | 3 | P450 | CYP042 | CLAN2 | CYP307C1 |
| 2952 | 1-1805 | plus | 381 | 4 PARTIAL | P450 | CYP029 | CLAN3 | CYP6PA1-partial |
| 308 | 327774-331448 | plus | 476 | 9 PARTIAL | P450 | CYP083 | CLANM | CYP314A1 |
| 315 | 498486-514286 | minus | 532 | 12 | P450 | CYP062 | CLAN4 | CYP380C23 |
| 315 | 425673-443577 | minus | 507 | 11 | P450 | CYP006 | CLAN4 | CYP380C26 |
| 315 | 473863-489932 | minus | 538 | 12 | P450 | CYP061 | CLAN4 | CYP380C28 |
| 315 | 528653-529786 | minus | 267 | 6 PARTIAL | P450 | CYP063 | CLAN4 | CYP380C-fragment1 |
| 327 | 135796-139332 | minus | 211 | 4 PARTIAL | P450 | CYP079 | CLAN4 | CYP4LS-fragment1 |
| 3321 | 79-838 | minus | 196 | 3 PARTIAL | P450 | CYP070 | CLAN4 | CYP4G196-fragment |
| 3560 | 114-686 | plus | 139 | 2 PARTIAL | P450 | CYP028 | CLAN3 | CYP6PB1-fragment |
| 36 | 710865-711305 | minus | 118 | 2 PARTIAL | P450 | CYP018 | CLAN2 | CYP15A1-fragment |
| 375 | 12670-14928 | plus | 505 | 4 | P450 | CYP086 | CLAN3 | CYP6CY26 |
| 375 | 37240-40488 | plus | 508 | 4 | P450 | CYP010 | CLAN3 | CYP6CY28 |
| 375 | 48361-50517 | plus | 525 | 4 | P450 | CYP069 | CLAN3 | CYP6PA1 |
| 375 | 18841-22424 | plus | 510 | 4 | P450 | CYP009 | CLAN3 | CYP6PB1 |
| 375 | 1961-5015 | plus | 374 | 3 PARTIAL | P450 | CYP087 | CLAN3 | CYP6PB2 |
| 375 | 25442-32804 | plus | 511 | 4 | P450 | CYP008 | CLAN3 | CYP6PC1 |
| 376 | 177366-187275 | minus | 553 | 9 | P450 | CYP074 | CLAN4 | CYP3373A1 |
| 379 | 222828-234500 | plus | 566 | 11 | P450 | CYP066 | CLAN4 | CYP4G194 |
| 379 | 250528-256115 | plus | 567 | 11 | P450 | CYP068 | CLAN4 | CYP4G195 |
| 379 | 239779-247927 | plus | 507 | 10 | P450 | CYP067 | CLAN4 | CYP4G196 |
| 386 | 105783-131499 | plus | 537 | 13 | P450 | CYP059 | CLAN4 | CYP380C22 |
| 386 | 45513-83895 | minus | 525 | 13 | P450 | CYP056 | CLAN4 | CYP380C24 |
| 386 | 2621-12905 | minus | 462 | 11 | P450 | CYP058 | CLAN4 | CYP380C27 |
| 386 | 27230-27907 | minus | 183 | 3 PARTIAL | P450 | CYP057 | CLAN4 | CYP380C-fragment4 |
| 39 | 142340-146225 | plus | 541 | 7 | P450 | CYP005 | CLAN3 | CYP6DB6 |
| 400 | 29114-42861 | minus | 463 | 10 | P450 | CYP065 | CLAN4 | CYP380C29 |
| 400 | 4589-7415 | minus | 129 | 3 PARTIAL | P450 | CYP090 | CLAN4 | CYP380C-fragment6 |
| 4382 | 36-615 | plus | 195 | 1 PARTIAL | P450 | CYP043 | CLAN3 | CYP6CZ4-fragment |
| 52 | 348966-353080 | plus | 511 | 5 | P450 | CYP012 | CLAN2 | CYP18A1 |
| 52 | 357022-361531 | minus | 496 | 6 | P450 | CYP011 | CLAN2 | CYP306A1 |
| 5421 | 36-859 | plus | 235 | 3 PARTIAL | P450 | CYP045 | CLAN3 | CYP6NZ1-fragment |
| 55 | 16484-18043 | minus | 293 | 7 PARTIAL | P450 | CYP064 | CLAN4 | CYP380C-fragment2 |
| 64 | 129440-134687 | plus | 448 | 6 PARTIAL | P450 | CYP076 | CLAN4 | CYP4LR1 |
| 717 | 47078..50042 | plus | 447 | 8 PARTIAL | P450 | CYP001 | CLANM | CYP353B1 |
| 72 | 282286..285221 | plus | 522 | 9 | P450 | CYP002 | CLANM | CYP301A1 |
| 72 | 289352..293786 | plus | 515 | 9 | P450 | CYP003 | CLANM | CYP301B1 |
| 742 | 59376-67317 | minus | 183 | 5 PARTIAL | P450 | CYP084 | CLAN4 | CYP380C-fragment5 |
| 82 | 166802-181391 | plus | 488 | 8 | P450 | CYP047 | CLAN2 | CYP305E1 |
| 82 | 154525-162736 | minus | 549 | 7 | P450 | CYP046 | CLAN4 | CYP4CH5 |
| 89 | 108086-113372 | minus | 522 | 11 | P450 | CYP085 | CLAN4 | CYP4CJ6 |
| 89 | 39494-51729 | minus | 518 | 11 | P450 | CYP013 | CLAN4 | CYP4CJ7 |
| 89 | 58439-69871 | minus | 383 | 9 PARTIAL | P450 | CYP016 | CLAN4 | CYP4CJ8 |
| 89 | 72157-86667 | minus | 412 | 10 PARTIAL | P450 | CYP015 | CLAN4 | CYP4LQ1 |
| 89 | 29291-38034 | minus | 513 | 11 | P450 | CYP014 | CLAN4 | CYP4LQ2 |
| 9715 | 252-530 | plus | 93 | 1 PARTIAL | P450 | CYP030 | CLAN3 | CYP6PC-fragment |

**Table S15.** Number of genes in detoxification families found in different insects and arthropods genomes. ND: not determined, **=ABC transporter numbers from *A. pisum* were obtained by automatic analysis. *= numbers based on EST data from [38]; *A. pisum* UGT sequences were reanalysed based on the latest genome release (AphidBase). Numbers in brackets are the numbers of previously described UGTs in [38,39]. CYP numbers for CYP from *Tetranychus urticae* were obtained from [40]. CYP data from *B. mori*, *T. castaneum*, *A. mellifera*, *N. vitripenis*, *D. melanogaster*, *A. gambiae*, and *A. pisum* from [41]. Data for *Bemisia tabaci* were obtained from [42].

## Table S15, continued.

| **Order** | | | **Hemiptera** | | | **Diptera** | | **Lepidoptera** | **Hymenoptera** | | **Coleoptera** | **Phthiraptera** | **Arachnid** |
|---|---|---|---|---|---|---|---|---|---|---|---|---|---|
| **Species** | *Daktulosphaira vitifoliae* | *Acyrthosiphon pisum* | *Rhodnius prolixus* | *Bemisia tabaci* | *Myzus persicae** | *Drosophila melanogaster* | *Anopheles gambiae* | *Bomby mori* | *Apis mellifera* | *Nasonia vitripennis* | *Tribolium castaneum* | *Pediculus humanus* | *Tetranychus urticae* |
| **Glutathione S-transferase** | | | | | | | | | | | | | |
| Glutathione S-transferase omega | 2 | 2 | 1 | ND | 1 (0) | 4 | 1 | 4 | 2 | 2 | 3 | ND | ND |
| Glutathione S-transferase epsilon | 0 | 0 | 0 | ND | 0 | 14 | 8 | 8 | 0 | 0 | 19 | ND | ND |
| Glutathione S-transferase sigma | 3 | 5 | 6(7) | ND | 7 (8) | 1 | 1 | 2 | 4 | 8 | 7 | ND | ND |
| Glutathione S-transferase delta | 2 | 11 | 1 | ND | 3 (2) | 11 | 17 | 5 | 2 | 5 | 3 | ND | ND |
| Glutathione S-transferase theta | 1 | 2 | 2(4) | ND | 2 (0) | 4 | 2 | 1 | 1 | 3 | 1 | ND | ND |
| Glutathione S-transferase Zeta | 0 | 2(0) | 1 | ND | 0 | 2 | 1 | 2 | 1 | 1 | 1 | ND | ND |
| Glutathione S-transferase unknown | 0 | 1 (3) | 0 | ND | 0 | 1 | 2 | 1 | 1 | 0 | 2 | ND | ND |
| Glutathione S-transferase microsomal | 3 | 2 | 1 | ND | 2 | 3 | 3 | 0 | 2 | 0 | 5 | ND | ND |
| **Total** | **11** | **25** | **13** | **ND** | **14** | **40** | **35** | **23** | **13** | **19** | **41** | ND | ND |
| **Uridine diphosphate-GlycosylTransferase (UGT)** | | | | | | | | | | | | | |
| UGTs | **35** | **55** | **ND** | **ND** | **38** | **34** | **26** | **45** | **12** | **22** | **43** | **ND** | **ND** |
| **Carboxyl/Cholinesterase (CCE)** | | | | | | | | | | | | | |
| Detoxification/dietary class | 2 | 5 | 22 | ND | 5 | 13 | 16 | 55 | 8 | 13 | 26 | 3 | 0 |
| Pheromone/hormone processing | 10 | 18 | 9 | ND | 12 | 8 | 14 | 8 | 5 | 17 | 11 | 2 | 2 |
| Neuro/developmental (total) | 8 | 8 | 12 | ND | 5 | 14 | 20 | 13 | 11 | 11 | 12 | 12 | 10 |
| clade H - Glutactin | 1 | 1 | 2 | ND | 0 | 5 | 10 | 0 | 1 | 1 | 1 | 0 | 2 |
| clade J - Acetylcholinesterase | 2 | 2 | 2 | ND | 3 | 1 | 2 | 2 | 2 | 2 | 2 | 2 | 1 |
| clade K - Gliotactin | 1 | 1 | 1 | ND | 1 | 1 | 1 | 1 | 1 | 1 | 1 | 1 | 1 |
| clade L - neuroligins | 3 | 3 | 4 | ND | 0 | 4 | 5 | 6 | 5 | 5 | 5 | 5 | 5 |
| clade M - neurotactine | 0 | 0 | 1 | ND | 0 | 2 | 2 | 2 | 1 | 1 | 2 | 3 | 1 |
| Unknown function | 1 | 1 | 2 | ND | 1 | 1 | 1 | 2 | 1 | 1 | 1 | 1 | 0 |
| Acari specific-class | | | | | | | | | | | | | 59 |
| **Total CCEs** | **20** | **31** | **43** | **51** | **22** | **35** | **51** | **76** | **24** | **41** | **49** | **17** | **71** |
| **ABC sub-family** | | | | | | | | | | | | | |
| A | 4 | 11 | ND | 8 | ND | 10 | 9 | 9 | 3 | ND | 10 | 2 | 9 |
| B | 6 | 9 | ND | 3 | ND | 8 | 5 | 9 | 5 | ND | 6 | 6 | 4 |
| C | 5 | 16 | ND | 6 | ND | 14 | 13 | 15 | 9 | ND | 35 | 5 | 39 |
| D | 3 | 2 | ND | 2 | ND | 2 | 2 | 2 | 2 | ND | 2 | 2 | 2 |
| E | 1 | 1 | ND | 1 | ND | 1 | 1 | 1 | 1 | ND | 1 | 1 | 1 |
| F | 3 | 4 | ND | 3 | ND | 3 | 3 | 3 | 3 | ND | 3 | 3 | 3 |
| G | 22 | 19 | ND | 23 | ND | 15 | 16 | 13 | 15 | ND | 13 | 13 | 23 |
| H | 23 | 9 | ND | 9 | ND | 3 | 3 | 3 | 3 | ND | 3 | 2 | 22 |
| **Total** | **67** | **71**** | **ND** | **55** | | **56** | **52** | **55** | **41** | | **73** | **40** | **103** |
| **Cytochrome P450 (CYP)** | | | | | | | | | | | | | |
| clan2 | 7 | 10 | 5 | 10 | 9 | 7 | 10 | 7 | 8 | 7 | 8 | 8 | 48 |
| clanmito | 6 | 8 | 6 | 7 | 7 | 11 | 9 | 12 | 6 | 7 | 9 | 8 | 5 |
| clan3 | 27 | 23 | 50 | 57 | 25 | 36 | 40 | 30 | 28 | 48 | 72 | 11 | 10 |
| clan4 | 41 | 23 | 23 | 56 | 19 | 32 | 46 | 34 | 4 | 30 | 45 | 9 | 23 |
| **Total** | **86** | **64** | **84** | **130** | **60** | **88** | **105** | **83** | **46** | **92** | **134** | **36** | **86** |

**Table S16.** Effector genes with predicted domains and their corresponding functions. The number of proteins with InterPro hits belonging to each cluster are indicated in parentheses.

| InterPro entry | Name | Function | Genes with IPR hits | Cluster number (IPR hits) |
|---|---|---|---|---|
| IPR001841 /IPR013083 | Zinc finger, RING-type | Mediating protein-protein interactions | 144 | 1(2), 3(37), 5(19), 15(45), 17(1), 18(13), 19(18), 38(2), 50(4), 67(1), 69(1), 78(1) |
| IPR020683 /IPR036770 | Ankyrin repeat-containing domain | Mediating protein-protein interactions | 133 | 49(133) |
| IPR002048 /IPR011992 | EF-hand domain | Calcium binding | 56 | 2(2), 7(11), 16(17), 29(22), 36(3), 44(1) |
| IPR019080 /IPR011335 | YqaJ viral recombinase | Processive alkaline exonuclease activity | 27 | 33(27) |
| IPR003172 /IPR036846 | MD-2-related lipid-recnition domain | Lipid recognition | 15 | 14(15) |
| IPR003323 /IPR038765 | OTU domain | Cysteine protease activity | 15 | 19(15) |
| IPR014044 /IPR035940 | CAP domain | Regulation extracellular matrix and branching morphogenesis, ion channel regulation, tumor suppressor, cell-cell adhesion | 14 | 13(14) |
| IPR001254 /IPR009003 | Serine proteases, trypsin domain | Serine protease activity | 6 | 23(6) |
| IPR032675 | Leucine-rich repeat domain | Mediating protein-protein interactions | 3 | 49(3) |
| IPR002350 /IPR036058 | Kazal domain | Proteinase inhibitor | 2 | 11(2) |
| IPR000971 | Globin | Binding and/or transporting oxygen | 1 | 3(1) |
| IPR007099 | RNA-directed RNA polymerase, negative-strand RNA virus | Catalyzing RNA synthesis | 1 | 3(1) |
| IPR009056 | Cytochrome c-like domain | Electron transfer activity, heme binding | 1 | 3(1) |
| IPR016024 | Armadillo-type fold | Protein/nucleic acid binding | 1 | 29(1) |

**Table S17.** Statistics on raw sequences used for assembling the complete genome of *D. vitifoliae*, with SRA accessions. Details are given for each type of library. Calculation of depth based on a genome size of 294 Mb. For PacBio, mean read length.

| Experiment (SRA Id) | type | Insert size | Read length | # bases sequenced | Depth |
|---|---|---|---|---|---|
| SRX7110322 | Illumina Paired Ends | 250 | 148 | 17.3G | 57.1 |
| SRX7110323 | Illumina Paired Ends | 500 | 150 | 7.7G | 25.7 |
| SRX7110324 | Illumina Paired Ends | 800 | 100 | 15.6G | 53.1 |
| SRX7110325 | Illumina Mate Pairs | 2000 | 100 | 5.4G | 18.3 |
| SRX7110326 | Illumina Mate Pairs | 5000 | 100 | 5.3G | 18 |
| SRX7110327 | PacBio | - | 6792 | 17.1G | 58 |

**Table S18.** List of taxa included in the study of gene expansions. Genome source (NCBI accessions or other databases) and number of predicted proteins are indicated for each taxon.

| Taxon | Source | No. predicted proteins |
|---|---|---|
| *Diaphorina citri* | PRJNA29447 | 19681 |
| *Myzus persicae* | PRJEB11304 (clone O), PRJNA319804 (clone G006) | 18378 |
| *Diuraphis noxia* | PRJNA296778 | 15072 |
| *Aphis glycines* | AphidBase | 18984 |
| *Rhopalosiphum padi* | AphidBase | 27554 |
| *Cinara cedri* | ongoing sequencing project | 16933 |
| *Daktulosphaira vitifoliae* | this study | 25567 |
| *Acyrthosiphon pisum* | PRJNA13657 | 23886 |
| *Bemisia tabaci* | PRJNA352527 | 19320 |

**Table S19.** Details on the sampling of phylloxera populations used for population genomics analyses. Populations corresponds to pools of insects collected in geographically close sites or to 'lineages' maintained in laboratory conditions as precised (comment column). SRA accession of each sequenced library in the population genomics study.

| Continent of origin | Country | Population (Pool) Name | SRA Accession | region/town | Native/introduced | nb of ind. | nb of sites/lineages pooled | GA/RA | Host plant | comment | cv. |
|---|---|---|---|---|---|---|---|---|---|---|---|
| North America | California | CA | SRX7116434 | - | native | 30 | 2 | Radicicole | *rootstock* | lineages maintained on 1103P | - |
| North America | Washington | WA | SRX7116435 | - | native | 50 | 2 | Radicicole | *Vitis labrusca* | | Concord |
| North America | Arizona | AR | SRX7116444 | - | native | 80 | 5 | Gallicole | *Vitis arizonica* | | - |
| North America | Wisconsin | WI | SRX7116445 | - | native | 60 | 3 | Gallicole | *Vitis riparia* | | - |
| North America | Ilinois | IL | SRX7116446 | - | native | 85 | 3 | Gallicole | *Vitis riparia* | | - |
| North America | New York | NY | SRX7116447 | - | native | 100 | 7 | Gallicole | *Vitis riparia* | | - |
| North America | Pennsylvania | PA | SRX7116448 | - | native | 37 | 1 | Gallicole | *Vitis vulpina* | | - |
| North America | Virginia | VA1 | SRX7116449 | - | native | 100 | 5 | Gallicole | *Vitis vulpina* | | - |
| North America | Virgnia | VA2 | SRX7116450 | - | native | 35 | 1 | Gallicole | interspecific hybrid | | Chambourcin |
| North America | West Virginia | WV | SRX7116451 | - | native | 31 | 2 | Gallicole | *Vitis aestivalis* | | - |
| North America | Massachusetts | MA | SRX7116436 | - | native | 20 | 1 | Gallicole | *Vitis labrusca* | | - |
| Europe | France | France | SRX7116437 | South-west | introduced | 100 | 6 | Gallicole | savaged rootstock | | - |
| Europe | Germany | Germany | SRX7116438 | Palatinat | introduced | 70 | 3 | Gallicole | savaged rootstock, *V. vinifera* | insects maintained on SO4 | Muscat Ottonel, Sylvaner |
| Europe | Austria | Austria | SRX7116439 | Vienna | introduced | 32 | 3 | Gallicole | *V. vinifera* | | Zweigelt, Cabernet |
| Europe | Romania | Romania | SRX7116440 | na | introduced | 100 | 4 | Gallicole | *V. vinifera* | | na |
| Europe | Armenia | Armenia | SRX7116441 | na | introduced | 40 | 1 | Gallicole | *V. vinifera* | | Rkatsiteli |
| South America | Uruguay | Uruguay | SRX7116442 | Montevideo | introduced | 100 | 2 | Gallicole | *V. vinifera* | | Marselan, Chardonnay |
| Australia | Australia | Australia | SRX7116443 | Victoria | introduced | 20 | 1 (G4) | Gallicole | *V. vinifera* | lineage maintained on 3309C | - |

**Table S20.** Prior distribution of parameters used for modelling the different scenarios of invasion routes (ABC methods). A) List of parameters and their characteristics. FR, IL and NY are the French, Illinois and New-York populations, respectively. B) Summary statistics: for all statistics, Mean and variance non-zero values and Mean of complete distribution were computed.

| **A. Parameter** | **Interpretation** | **Distribution** | **Range** | **Conditions** |
|---|---|---|---|---|
| **Introduction in France** | | | | |
| N0 | Effective size of the FR founder population | Uniform | [10-10000] | N0<N2,N0<N3 |
| N1 | Effective size of the FR population | Uniform | [10-10000] | |
| N2 | Effective size of the IL population | Uniform | [10-10000] | |
| N3 | Effective size of the NY population | Uniform | [10-10000] | |
| N4 | Effective size of the original US population | Uniform | [10-10000] | |
| N5 | Effective size of founder from IL population | Uniform | [10-10000] | N5<N2 |
| N6 | Effective size of founder from NY population | Uniform | [10-10000] | N6<N3 |
| t0 | Time to invasion | Uniform | [10-10000] | |
| tn | Time to merge event between original population and NY population | Uniform | [10-10000] | tn>t5,tn>tf |
| ti | Time to merge event between original population and IL population | Uniform | [10-10000] | ti>t6 |
| tf | Time to merge event between the FR population and the invasive population | Uniform | [10-10000] | tf<ti,tf>t0 |
| t5,t6 | Time to merge between the invasive population coming founder population and their respective origin populations | Uniform | [10-10000] | t5>tf,t6>tf |
| ra | ratio of admixture | Uniform | [0.001-0.999] | |
| **Introduction in Germany (after France)** | | | | |
| N1 | Effective size of the FR population | Uniform | [10-10000] | |
| N2 | Effective size of the German population | Uniform | [10-10000] | |
| N3 | Effective size of the IL population | Uniform | [10-10000] | |
| N3b | Effective size of the German founder population from IL | Uniform | [10-10000] | N3b<N2,N3b<N3 |
| N4 | Effective size of the original NY population | Uniform | [10-10000] | N4b<N4,N4b<N2 |
| N4b | Effective size of German founder population from NY | Uniform | [10-10000] | |
| N5 | Effective size of FR founder population from IL | Uniform | [10-10000] | N5<N3,N5<N1 |
| N6 | Effective size of FR founder population from NY | Uniform | [10-10000] | N6<N4,N6<N1 |
| N7 | Effective size of the original US population | Uniform | [10-10000] | |
| N8 | Effective size of German founder population from IL | Uniform | [10-10000] | N8<N3,N8<N2 |
| N9 | Effective size of German founder population from NY | Uniform | [10-10000] | N9<N4,N9<N2 |
| tg | Time to merge event between the German founder and its ancestral population | Uniform | [10-10000] | tg>tg1 |
| tg1 | Time to merge event between the German opulation and its founder population | Uniform | [10-10000] | |
| tf | Time to split event between the FR population and the invasive population | Uniform | [10-10000] | tf<tg |
| t5,t6, t8, t9 | Time to merge between the invasive populations coming founder population and their respective origin populations | Uniform | [10-10000] | t5>tf,t6>tf,t8>tg,t9>tg, |
| tn | Time to merge event between original population and NY population | Uniform | [10-10000] | tn>t5,tn>t6,tn>t8,tn>tg |
| ti | Time to merge event between original population and IL population | Uniform | [10-10000] | ti>t9,ti>t6,ti>tg,ti>t5,ti>t8 |
| ra, rb | ratio of admixture | Uniform | [0.001-0.999] | |

| **B. Summary statistics used for all scenarios** | |
|---|---|
| One sample statistics | Genetic Diversities |
| Pairwise statistics | FST distances |
| | Nei's distances |
| Admixture statistics | All estimates |

# Supplementary Methods and Results

## microRNAs

**Methods:** Total RNA was isolated from a whole colony of the isofemale INRA-Pcf7 clone using the MirVana kit (Ambion) that allows to enrich the preparation for small RNAs shorter than 200 nucleotides. Small RNAs were sequenced at the IGBMC laboratory (Illkirch, France) by 50 bp single reads, with the Illumina TruSeq technology on a HiSeq4000,. The 188 millions reads, after clipping adapters (with clip_adapters.pl from mirDeep2 TGGAATTCTCGGGTGCCAAGG) were mapped to the genome with and collapsed with the mapper.pl script from mirDeep2 with the options “-j -l 18 -m”. Finally, the resulting arf file was processed with miRDeep2.pl with the *A. pisum* mature sequences from miRBase as reference. The structures of precursor sequences returned from miRDeep2 were manually inspected. Any predicted miRNAs that lacked significant support for a hairpin structure, or candidate star and loop sequences were excluded. The predicted precursors and mature sequences of miRNAs were blasted against miRBase v21 using miRBase implemented blastn to assign known miRNAs and novel miRNAs [43]. miRNA duplications were analyzed with CodonCode Aligner v.5.1.5 (CodonCode Corporation: http://www.codoncode.com/aligner/index.htm), while miRNA clusters were manually annotated.

**Results:** Using small RNA-Seq data, and after automatic prediction and manual curations, 150 miRNA precursors encoding 142 unique mature miRNAs were identified (Figure S12); of these 69 (49%) were homologous to miRNAs previously reported in other species, while 73 (51%) were novel (Table S10). 31% (*i.e.* 47/150) of miRNA precursors were arranged in 14 genomic clusters and 42% percent (*i.e.* 63/150) were derived from 23 miRNA duplications, with one group containing 12 duplicates (Table S10). With respect to genomic localization, 63% (*i.e.* 95/150) of miRNA precursors were found in intergenic regions (IGR), 31% (*i.e.* 46/150) were in introns, and 6% (*i.e.* 9/150) were in exons, UTRs, or UTR/exon junctions. Lastly, eight genomic sites expressed miRNAs from both +/- DNA strands.

## Aquaporins

**Methods:** Aquaporins (AQPs) function as a channel for the passage of water and other small molecules across cell membranes [44]. These genes vary considerably in numbers and sequence identity among phloem sap-feeding insect groups [45]. The amino acid sequences of aquaporins were obtained from AphidBase for *A. pisum* and from Genbank for other species. Aquaporins of phylloxera were identified by blastp against the predicted phylloxera proteome, with *A. pisum* and *D. melanogaster* aquaporins as queries, and gene models were manually curated. Amino acid sequences were aligned using MAFFT [46] and trimmed using TrimAl [47]. A Bayesian analysis was conducted with MrBayes 3.2.6 using the model LG+I+G [48]. The Bayesian Inference (BI) model was determined using ProtTest 3.4.1 [49]. In total, 10,000 trees were obtained (ngen= 1,000,000; samplefreq=100) and the first 25% were discarded as burn-in. The average split frequencies were < 0.01. The consensus phylogenetic tree was visualized with FigTree.

**Results:** We found three *AQP* genes in the genome of grape phylloxera, with one in each of three clades: *Drosophila* integral protein (*DRIP*), entomoglyceroporin (*EGLP*) and big brain (*BIB*). These sequences had the expected canonical amino acid residues, indicating that the proteins are functional. We found these three genes to have 1:1 orthologs in *A. pisum* (Figure S13), suggesting that they have not been duplicated. By contrast, Aleyrodoidea (whiteflies) and Psylloidea have multiple genes in the different aquaporin gene families, including PRIP-aquaporins [45] which are absent from both phylloxera and *A. pisum*.

## Methylation

**Methods:** Putative DNA methylation-related genes were discovered using tblastn queries from pea aphid and *Apis mellifera* sequences. Pea aphid sequences used were Dnmt1 (ACYPI073296), Dnmt2 (ACYPI00794), Dnmt3 (ACYPI29975), methyl-CpG-binding protein (ACYPI004592), and Dnmt1-associated protein (ACYPI004738). Corresponding *Apis mellifera* sequences used were XP_026298868, XP_006563008, XP_026302148, XP_003250633, and XP_392117, respectively. Hits above 1e-50 were reverse blasted back to the pea aphid genome for verification. Identification of homologs was followed by manual curation of gene models.

**Results:** The DNA methylation machinery consists of proteins such as Dnmt1, Dnmt2, Dnmt3, methyl-CpG-binding protein, and Dnmt1-associated protein. Genes coding for these proteins were found in the grape phylloxera genome, each with only a single copy. The grape phylloxera thus has genes necessary for DNA methylation.

## Circadian clock and related genes

**Methods:** Annotation of circadian clock genes was performed using the corresponding protein sequences from *Acyrthosiphon pisum* (Table S11) as blastp or tblastn queries against *Phylloxera* predicted transcripts or genome (e-value threshold, 1e-6). This was followed by manual curation of gene models for hits.

**Results:** The circadian clock mechanism which is conserved among different taxa, consists of two interlocked feedback loops of transcription factors [50], organised into three groups of elements: the input, core and output of the clock. Based on the sequences of the *A. pisum* circadian clock genes [51-52] (Table S11), we found that these three groups are conserved in the phylloxera in terms of presence or absence of their members. The circadian clock of grape phylloxera belongs to the insect ancestral type [53], which is mainly characterized by the presence of two different types of cryptochromes (CRY): a *Drosophila*-like CRY (d-CRY or CRY1) and a vertebrate-like CRY (v-CRY or CRY2). Phylloxeran circadian clock genes are highly similar to those found in *A. pisum*. As in *A. pisum*, some of them seem to have experienced accelerated evolutionary rates especially Dvit*per* (Figure S14), Dvit*tim* and Dvit*cry2 (not shown)*. A gene encoding the prothoracicotropic hormone (PTTH) was also identified which, as in aphids [54] was characterized by the presence of six cysteine residues rather than the more typical seven (Figure S15). While several elements of the circadian clock appear to be duplicated in the *A. pisum* genome, we found fewer or no duplications in phylloxera (e.g. no duplication of *cry2*, or *Rhodopsin 7*, and two instead of four copies of *iAANAT* and *SWO*).

## Adaptation to biotic and abiotic environment

**Methods:**

i) Odorant and gustatory receptors: OR and GR sequences (ORs: 599 sequences, GRs: 190 sequences) from other hemipteran species were retrieved from the nr database (July 2017) and used against the grape phylloxera genome using tblastn. Respectively 46 and 13 scaffolds presented a hit with OR or GR query sequences. Hemipteran chemoreceptor amino acid sequences (ORs and GRs) were aligned on these scaffolds using Scipio [55], Exonerate [56] and Genewise [57]. These alignments were used to generate gene models using Apollo [58] which were manually curated based on sequence similarity with other species and on RNAseq data. To build a phylogeny, amino acid sequences of candidate ORs and GRs of phylloxera were added to sequences from other Hemiptera: *A. pisum* (Apis, ORs and GRs) [35], *R. prolixus* (Rpro, ORs and GRs) [34] and *A. gossypii* (Agos, ORs only) [59]. Sequences with less than 300 amino acids were removed from the data sets. Sequences were aligned using MAFFT [60] and a phylogeny was built using PhyML 3.0 [61] . The best-fit model of protein evolution was determined using SMS [62]. Node support was assessed by carrying out an approximate likelihood-ratio test aLRT [63]

ii) Odorant, gustatory ligand-binding and extracellular-binding families:  We first examined preliminary annotations of the phylloxera genome to assign genes to odorant, gustatory ligand-binding and extracellular-binding families based on the detection of a Pfam domain for OBPs, CSPs and NPC2s [64]. Then we built a protein database containing all protein sequences of this first set completed by members of these families previously characterized in the genome of *A. pisum* [65] . Using this collection as query sequences, we searched the genome sequence using tblastn (*E*-value < $10^{-3}$) to find additional members. All new annotations were visually checked using RNA-Seq data and SNP coverage density curves. We then conducted a phylogenetic analysis of the three principal families (OBP, CSP and NPC2 families) including only full-length proteins for phylloxera, and a set of representative sequences from aphid species - a different set of species was used in each of the three analyses. Overall this list of species comprised *A. pisum* (Apis), *A. gossypii*

(Agos), *D. noxia* (Dnox), *M. persicae* (Mper), *Sitobion avenae* (Save), *A. glycines* (Agly), *Nasonovia ribisnigri* (Nrib) *and Metopolophium dirhodum* (Mdir). Protein sequences were aligned using MAFFT v7.273 [66] and RAxMLHPC-AVX v8.2.4 [67] was used to obtain the maximum likelihood phylogenetic relationships within each family. Node support was calculated using 500 bootstrap replicates. An analysis of gene conversion across paralogues was carried out using GeneConv [68] . The resulting trees were edited using iTOLv3.5.4 [69].
iii) Detoxification genes: We first analyzed Cytochrome P450 (CYP) proteins, a group in which sequence identity can be as low as 25 percent, but where conserved motifs allows assignment to CYP sequences [70]. The phylloxera genome was searched for P450 genes by tblastn against the whole genome sequence, using annotated CYP protein sequences from *A. pisum* and *M. persicae* as queries. Candidate genomic regions containing CYPs were manually annotated to confirm or correct gene models with the help of RNA-Seq data when available (these curation steps were also done in the next groups of detoxification genes). Protein CYP sequences were sent to D. Nelson for name attribution. For GSTs and UGTs, we used sets of Hemiptera proteins to search the phylloxera genome by tblastn using Galaxy [71], and then Exonerate and Scipio to align protein sequences on the genome and to define intron/exon boundaries [55]. The classification of deduced proteins and their integrity were verified using blastp against the non-redundant (nr) GenBank database. When genes were suspected to split in different scaffolds, protein sequences were merged for further analyses. All active sites were confirmed using the NCBI CD-search program. CCE genes were searched by tblastn against the whole genome assembly using CCE proteins sequences from *N. vitripennis*, *R. prolixus*, *A. pisum* and *M. persicae* collected from the latest NCBI release. Then CCE sequences were analyzed as described above for UGTs/GSTs. ABC transporters genes were searched by tblastn against the genome sequence using ABC transporter proteins sequences from *D. melanogaster* and *A. mellifera*.

**Results:** Sensing and reacting to the biotic and abiotic environment involves chemical senses, as well as detoxification and secretion of effectors. Genes associated with these functions often evolve rapidly, allowing adaptation to new

environments. Although little is known about the chemical ecology of grape phylloxera, it has been shown that this species exhibits selectivity to grape rootstocks and cultivars that produce different volatile organic compounds [72]. This suggests that phylloxera uses chemical cues, as suggested above by the GO term enrichment seen in the phylloxera lineage-specific set of genes. We annotated odorant (ORs) and gustatory (GRs) receptors in the phylloxera genome, this being the first time for a root-feeding herbivore. We annotated 60 OR genes (Dvit_OR), plus one gene encoding the co-receptor ORco (Table 12). While *ORco* sequences are highly conserved, other hemipteran ORs are very divergent (Figure 16). Our phylogenetic analysis suggests that evolution of the ORs is mostly characterized by lineage-specific expansions. In particular, almost half of Dvit_ORs (29 out of 60) belong to a well supported clade containing no aphid OR. We found no ortholog of *Apis_OR5*, underlying production of the alarm pheromone of *A. pisum* [73]. This fits with the absence in phylloxera of both alarm pheromones and cornicles, the organs that emit pheromones in aphids. A total of 18 GRs (Dvit_GRs) were annotated, less than in other Hemiptera species (*A. pisum*: n=70, *R. prolixus*: n=26). Three Dvit_GRs belonged to a clade including putative *A. pisum* sugar receptors [35] (Figure 17). While $CO_2$ may be one of the volatile cues for root detection, no candidate $CO_2$ receptor could be found, just as in the *A. pisum* and *R. prolixus* genomes.
We also identified a total of 13 odorant-binding (*Obp*), 12 chemosensory (*Csp*) and 8 Niemann-Pick (*Npc2*) genes, encoding soluble proteins regarded as possible carriers of sensory cues in insect chemoreception [74] (Table 13). These numbers are very similar to those found in *A. pisum* (13, 12 and 7, respectively) and in other aphids [37,65,75]. We did not find members of the CheA/B superfamily in the phylloxera genome. Our phylogenetic analysis of the OBP gene family (Figure 18) identified 11 orthogroups. Grape phylloxera was represented in every orthogroup except OBP7, and aphid species were represented in all of them, suggesting that the diversification of this gene family preceded the divergence between phylloxera and aphids. Some orthogroups (e.g. OBP14) were missing from one or a few species, indicating gene turnover during the evolution of this family. The CSP tree (Figure 19) shows a similar picture to that of the OBP tree, although, in this case, the lower number of species available for the analysis precluded clear conclusions about gene

turnover dynamics. The phylogenetic tree of the NPC2 proteins presented a different aspect, with multiple copies from the same species clustering together, with no clear orthogroups (Figure 20). As we did not detect evidence of gene conversion between paralogs, we suggest that the NPC2 family is highly dynamic both in aphids and phylloxera, with multiple lineage-specific duplications in each group. Concerning the detoxification function, the genome of grape phylloxera contained 86 cytochrome P450s (CYPs), 35 UDP-glycosyltransferases (UGTs), 11 glutathione S-transferases (GSTs), 20 carboxylesterases (CCEs) and 67 ABC transporters (Table 14, Table 15). We found that these five classes of genes have seen duplication events both preceding and following the divergence between phylloxera and aphids. Compared to aphids, phylloxera generally had a reduced number of copies though it did show expansion of particular clusters. In the case of the CYPome, a very dynamic gene family [41], two CYP families, *CYP6CZ* and *CYP380C*, were expanded in phylloxera while the CYP6CY family comprised only six members compared to 16 and 12 in *A. pisum* and *M. persicae*, respectively. Finally, a new *CYP* family, *CYP3373*, was found in the phylloxera genome.
